# Supplementary material for: Evaluation and Assessment of the ABATE Framework to Enhance Implicit Bias Training for Virtual Interviews in Medical Schools
Source: MedEdPORTAL. 2024 Jun 28;20:11416. doi: 10.15766/mep_2374-8265.11416 (PMC11219124; doi:10.15766/mep_2374-8265.11416)
Supplement: Supplementary file 1 — ABATE Framework.docxLevels of Implementation.docxPreworkshop Evaluation Questionnaire.docxPostworkshop Evaluation Questionnaire.docxABATE Slide Deck.pptxABATE Speaker Notes.docx [file mep_2374-8265.11416-s001.zip › D. Postworkshop Evaluation Questionnaire.docx]

Following the debrief discussion, direct the attendees to complete this Post-workshop Questionnaire. Digitize the questionnaire using Google Forms or Microsoft Forms and provide a link through Zoom chat. *(Activity duration = 5 minutes)*

**Post-workshop Questionnaire**

1. My prior training has adequately prepared me to minimize implicit bias during virtual interviews.

- Strongly Disagree
- Disagree
- Neutral
- Agree
- Strongly Agree

1. I am confident in my abilities to conduct an unbiased virtual interview.

- Strongly Disagree
- Disagree
- Neutral
- Agree
- Strongly Agree

1. I am comfortable with saying that I am aware of any potential sources of implicit bias that may occur during virtual interviews.

- Strongly Disagree
- Disagree
- Neutral
- Agree
- Strongly Agree

1. I have experience with applying and identifying solutions that reduce implicit bias during my virtual interviews.

- Strongly Disagree
- Disagree
- Neutral
- Agree
- Strongly Agree

1. Based on your knowledge of the ABATE model for identifying bias please assess this statement: Generally, an interviewer's perception of “likeness,” or membership to an “ingroup” puts candidates at an advantage.

- Strongly Disagree
- Disagree
- Neutral
- Agree
- Strongly Agree

1. Based on your knowledge of the ABATE model for identifying bias please assess this statement: To address the potential undue influence of interview backgrounds on affinity bias and cultural matching, admissions offices and interview teams can identify strategies to neutralize the ways in which the background contributes to implicit bias.

- Strongly Disagree
- Disagree
- Neutral
- Agree
- Strongly Agree

1. Based on your knowledge of the ABATE model for identifying bias please assess this statement: A candidate that is overweight doesn't understand what it means to be a leader and will not be as successful in medical school than other candidates who are not overweight.

- Strongly Disagree
- Disagree
- Neutral
- Agree
- Strongly Agree

1. Based on your knowledge of the ABATE model for identifying bias please assess this statement: Since we’ve all had to adjust virtually during the pandemic, there is no bias surrounding an interviewee's ability to make eye contact during virtual interviews.

- Strongly Disagree
- Disagree
- Neutral
- Agree
- Strongly Agree

1. Based on your knowledge of the ABATE model for identifying bias please assess this statement: The way one enunciates and pauses in their speaking has nothing to do with how you assess them.

- Strongly Disagree
- Disagree
- Neutral
- Agree
- Strongly Agree

1. Please let us know what your big takeaways from this training are and if you have any additional comments.
