# Supplementary material for: Evaluation and Assessment of the ABATE Framework to Enhance Implicit Bias Training for Virtual Interviews in Medical Schools
Source: MedEdPORTAL. 2024 Jun 28;20:11416. doi: 10.15766/mep_2374-8265.11416 (PMC11219124; doi:10.15766/mep_2374-8265.11416)
Supplement: Supplementary file 1 — ABATE Framework.docxLevels of Implementation.docxPreworkshop Evaluation Questionnaire.docxPostworkshop Evaluation Questionnaire.docxABATE Slide Deck.pptxABATE Speaker Notes.docx [file mep_2374-8265.11416-s001.zip › E. ABATE Slide Deck.pptx]

## Slide 1
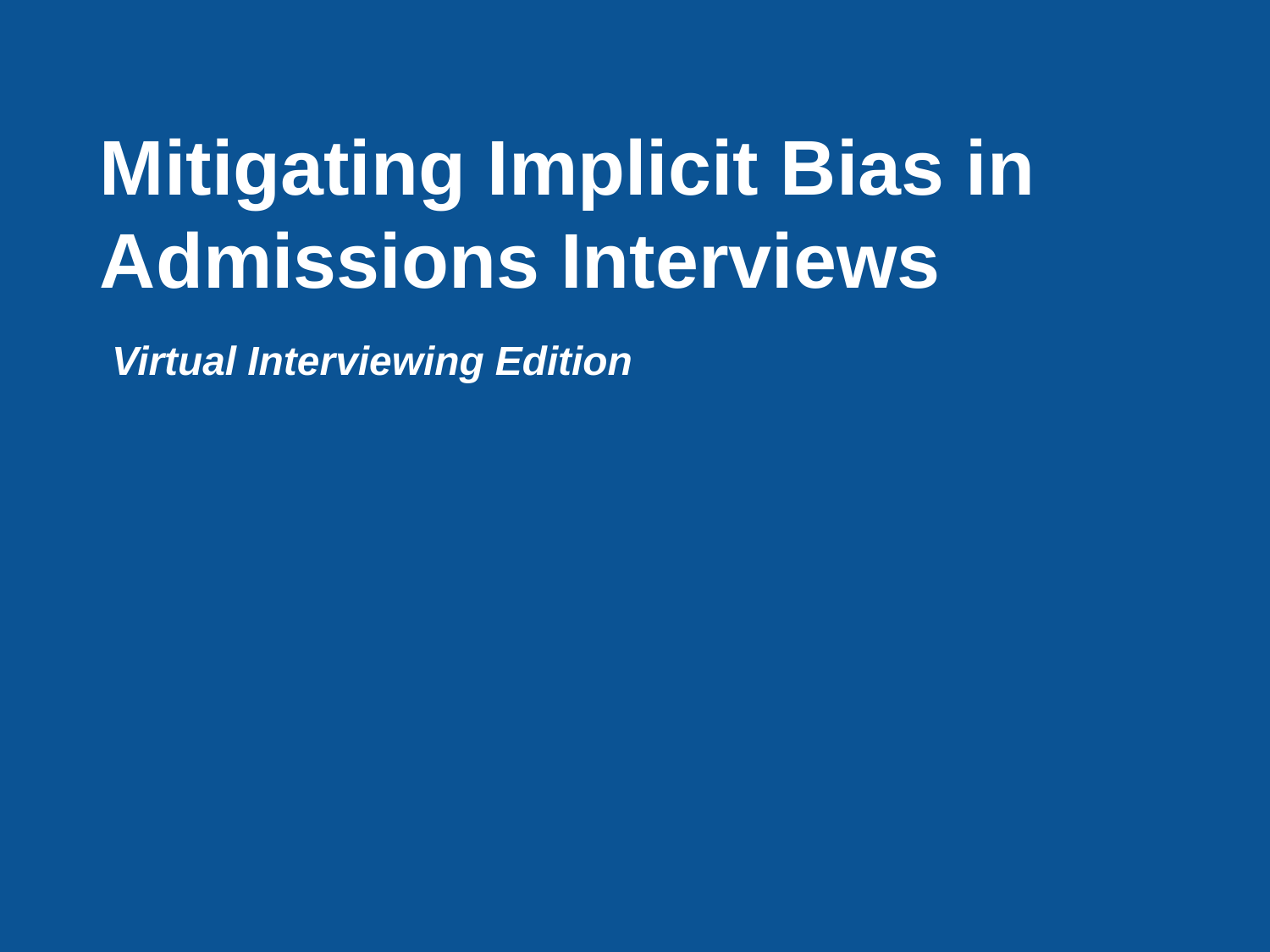

# Mitigating Implicit Bias in Admissions Interviews
Virtual Interviewing Edition

## Slide 2
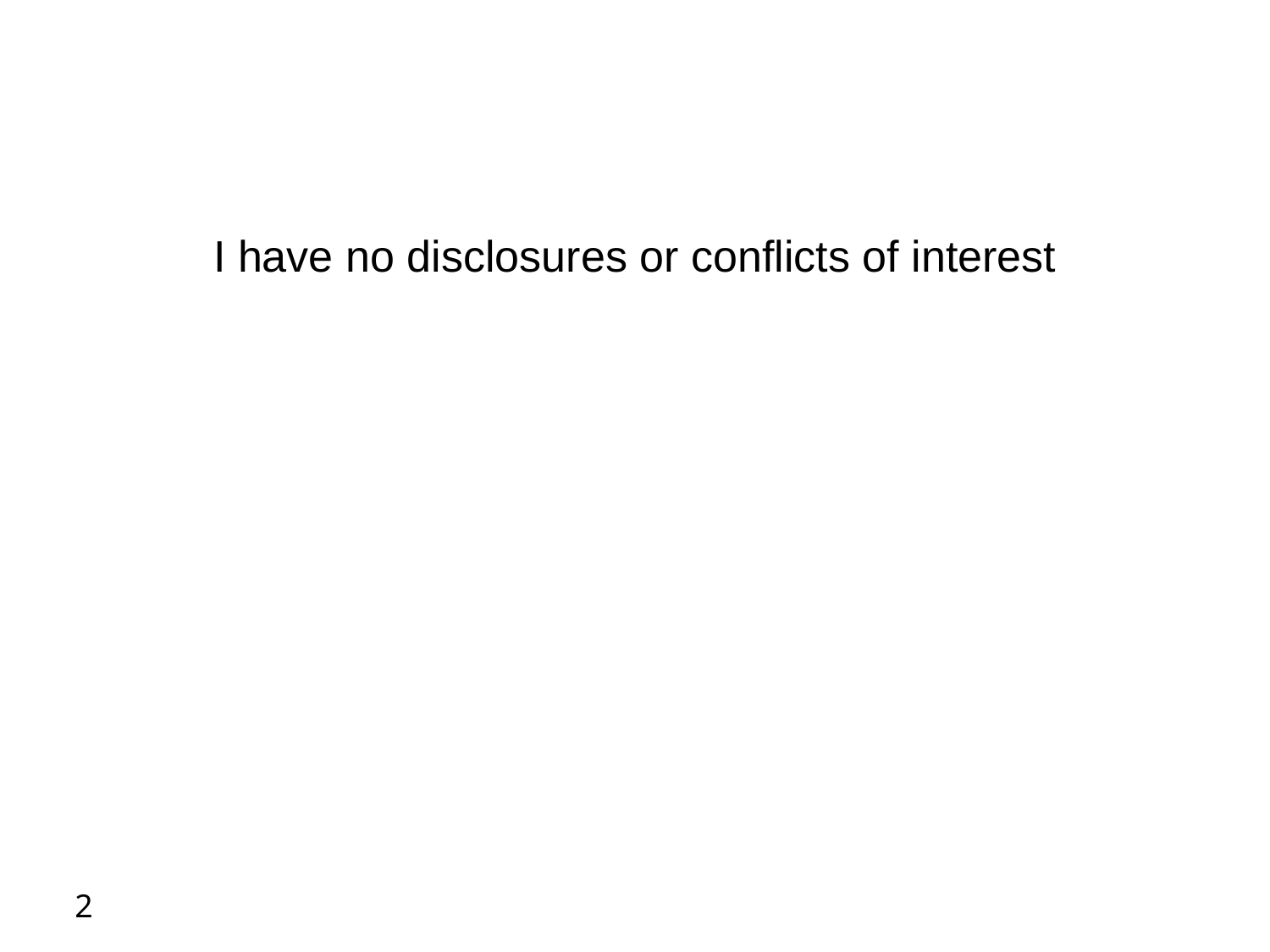

I have no disclosures or conflicts of interest
2

## Slide 3
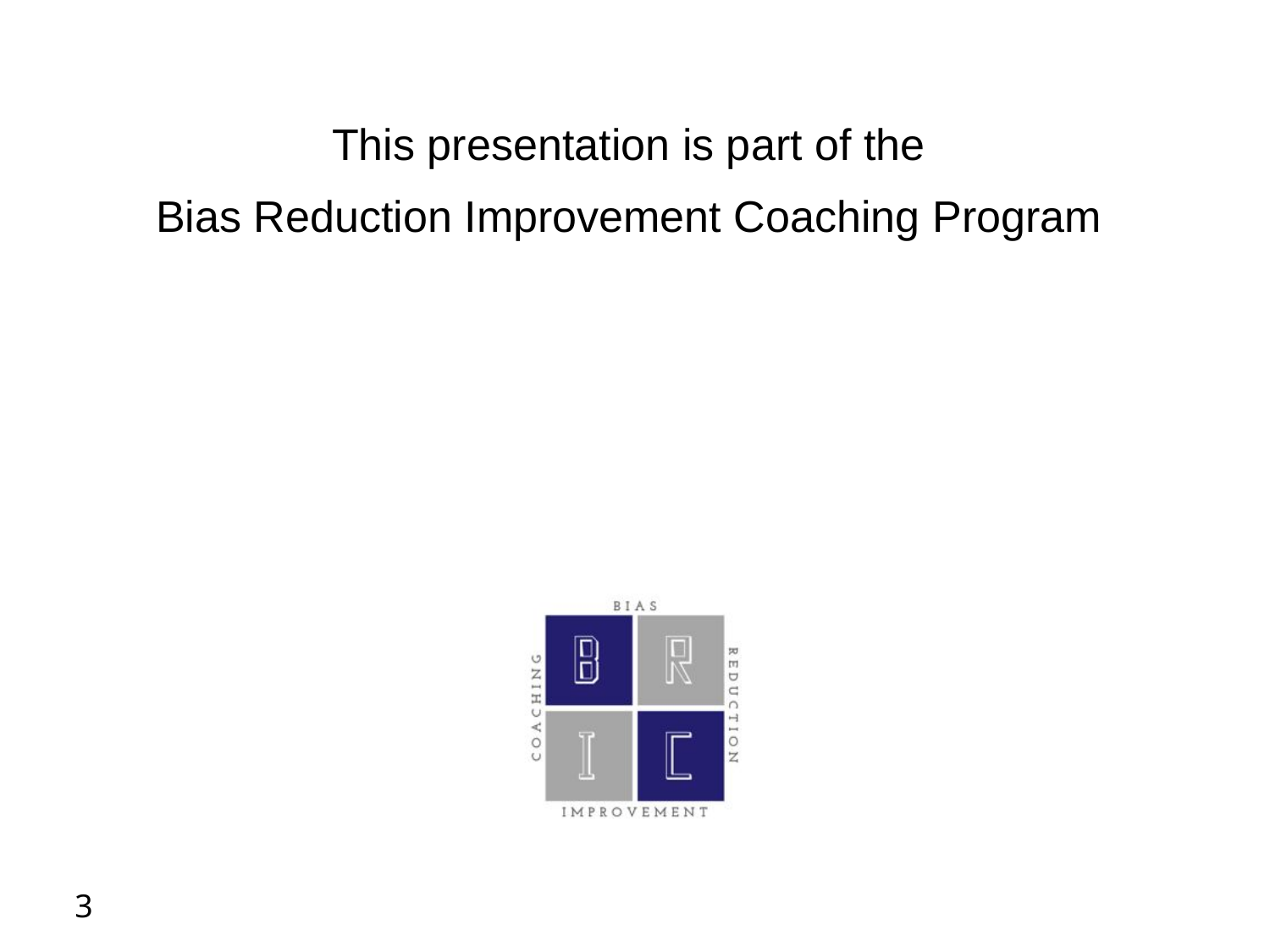

This presentation is part of the
Bias Reduction Improvement Coaching Program
3

## Slide 4
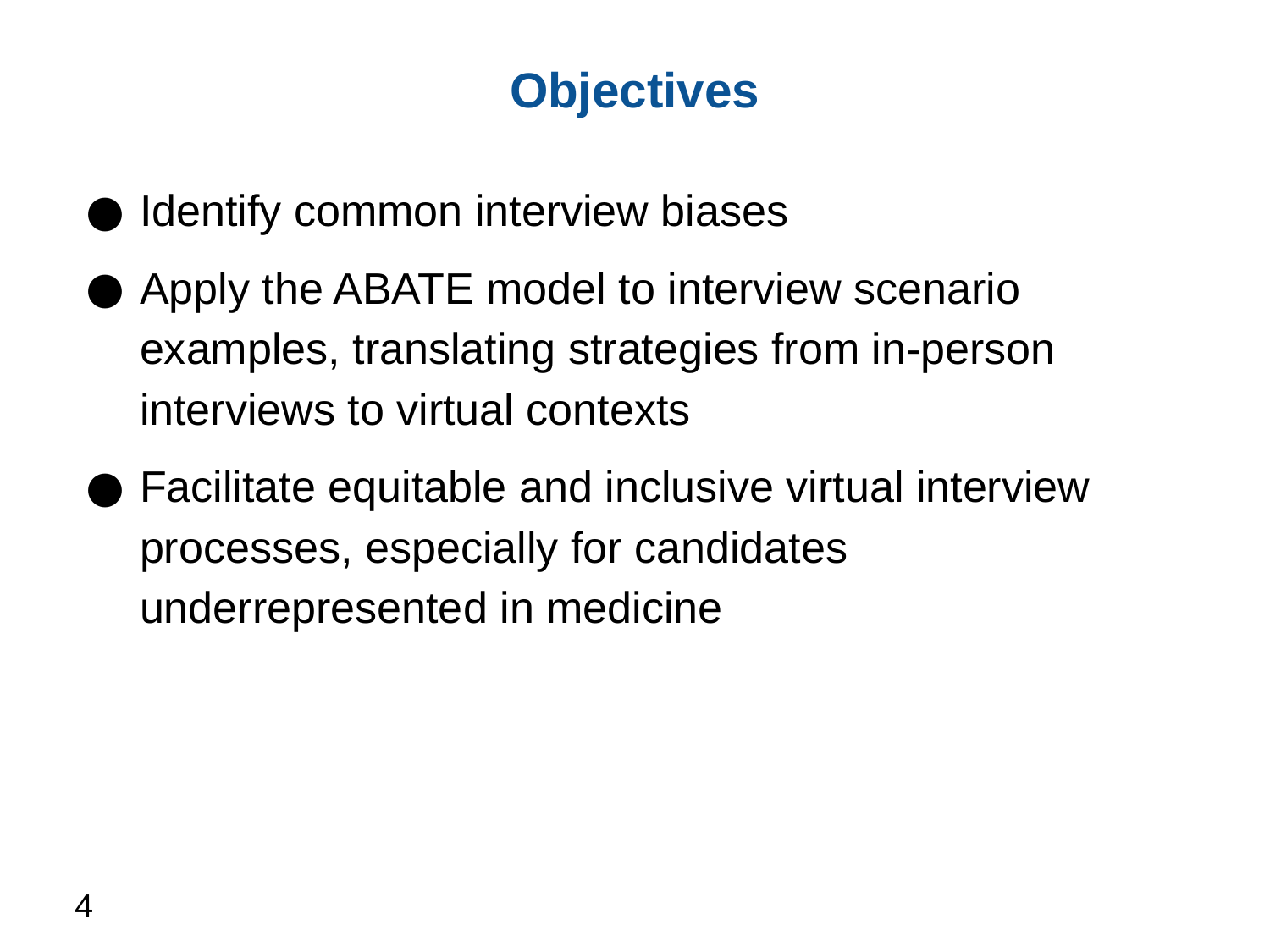

# Objectives
Identify common interview biases
Apply the ABATE model to interview scenario examples, translating strategies from in-person interviews to virtual contexts
Facilitate equitable and inclusive virtual interview processes, especially for candidates underrepresented in medicine
4

## Slide 5
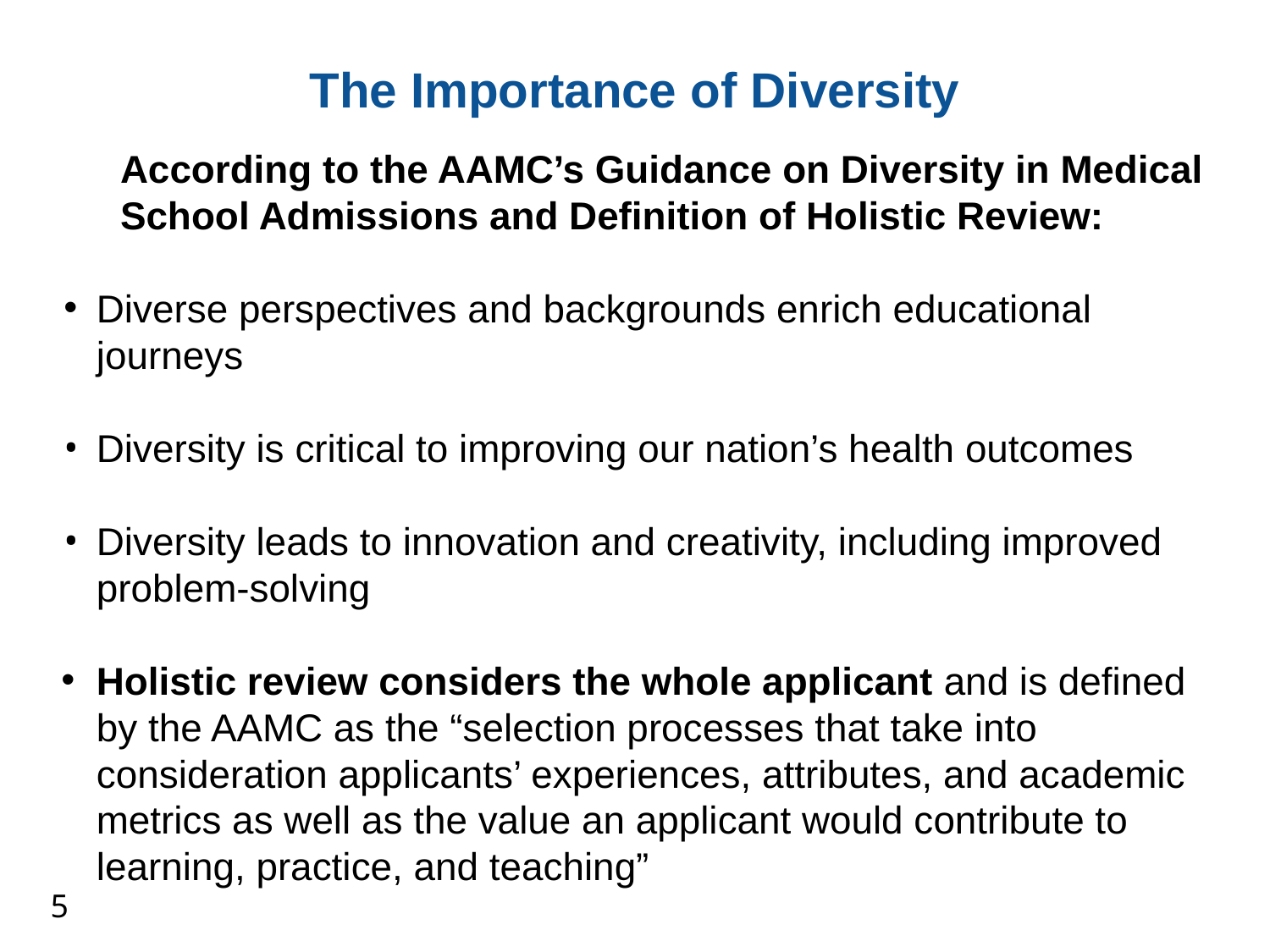

# The Importance of Diversity
According to the AAMC’s Guidance on Diversity in Medical School Admissions and Definition of Holistic Review:
Diverse perspectives and backgrounds enrich educational journeys
Diversity is critical to improving our nation’s health outcomes
Diversity leads to innovation and creativity, including improved problem-solving
Holistic review considers the whole applicant and is defined by the AAMC as the “selection processes that take into consideration applicants’ experiences, attributes, and academic metrics as well as the value an applicant would contribute to learning, practice, and teaching”
5

## Slide 6
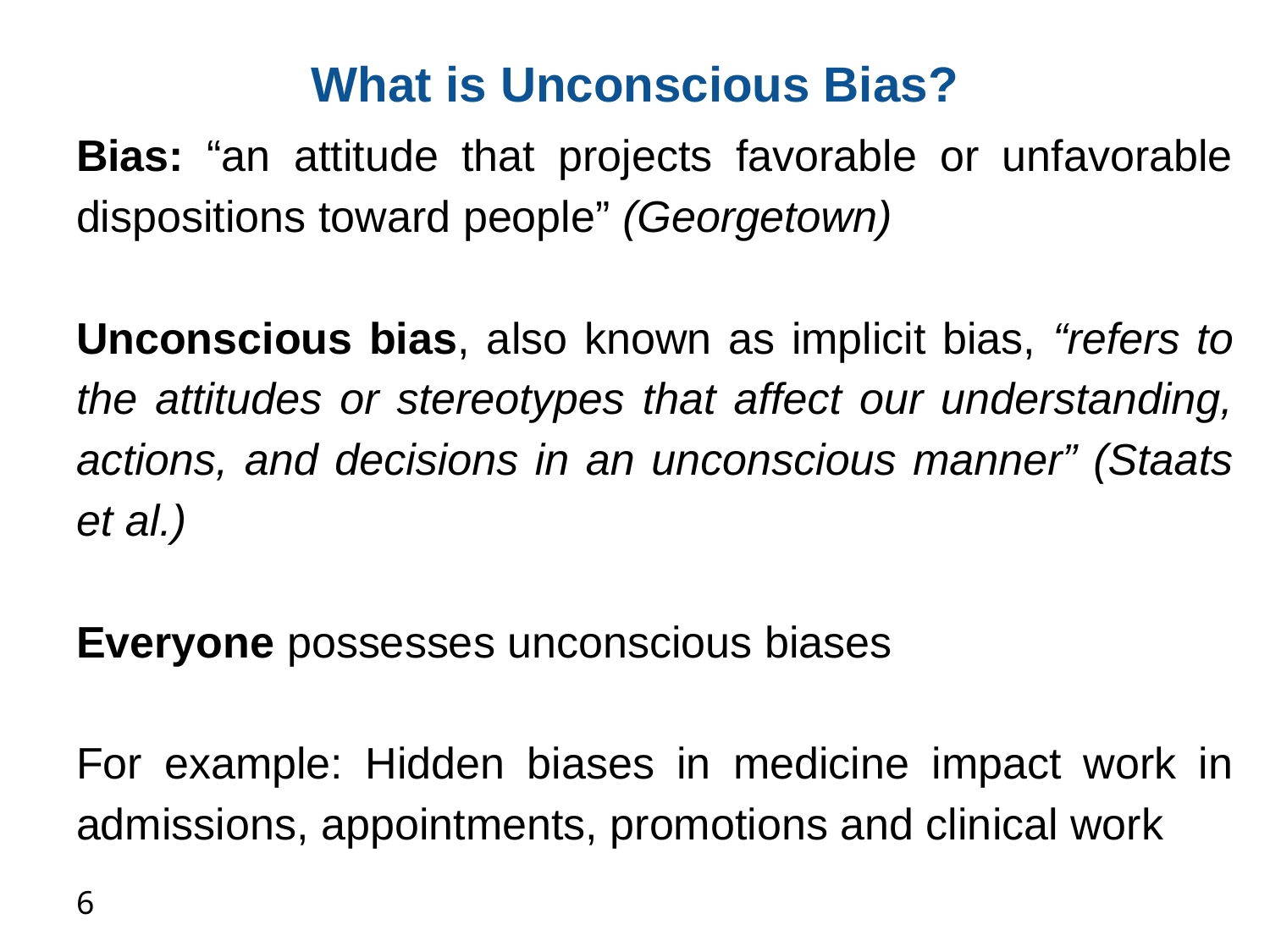

# What is Unconscious Bias?
Bias: “an attitude that projects favorable or unfavorable dispositions toward people” (Georgetown)
Unconscious bias, also known as implicit bias, “refers to the attitudes or stereotypes that affect our understanding, actions, and decisions in an unconscious manner” (Staats et al.)
Everyone possesses unconscious biases
For example: Hidden biases in medicine impact work in admissions, appointments, promotions and clinical work
6

## Slide 7
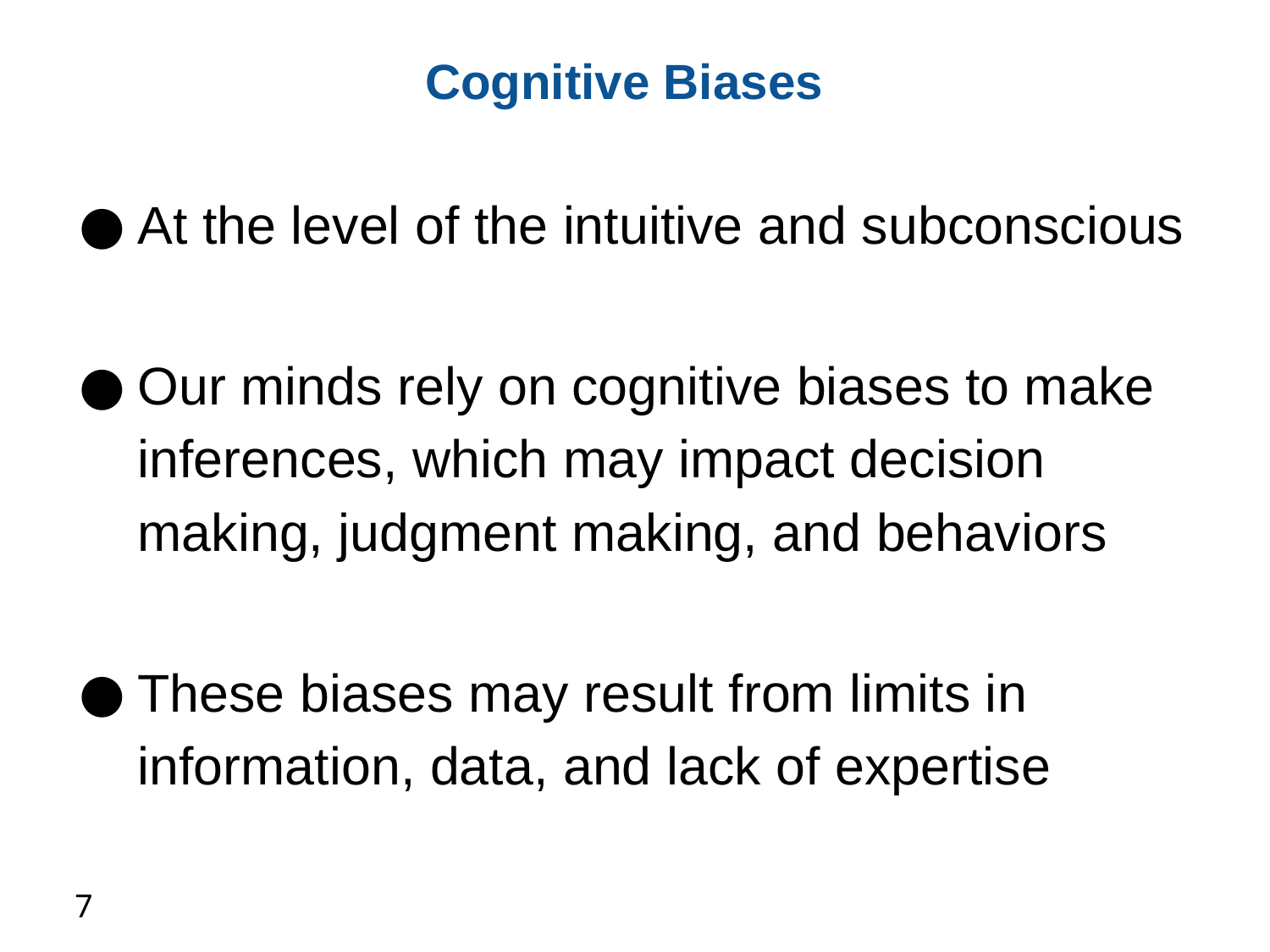

# Cognitive Biases
At the level of the intuitive and subconscious
Our minds rely on cognitive biases to make inferences, which may impact decision making, judgment making, and behaviors
These biases may result from limits in information, data, and lack of expertise
7

## Slide 8
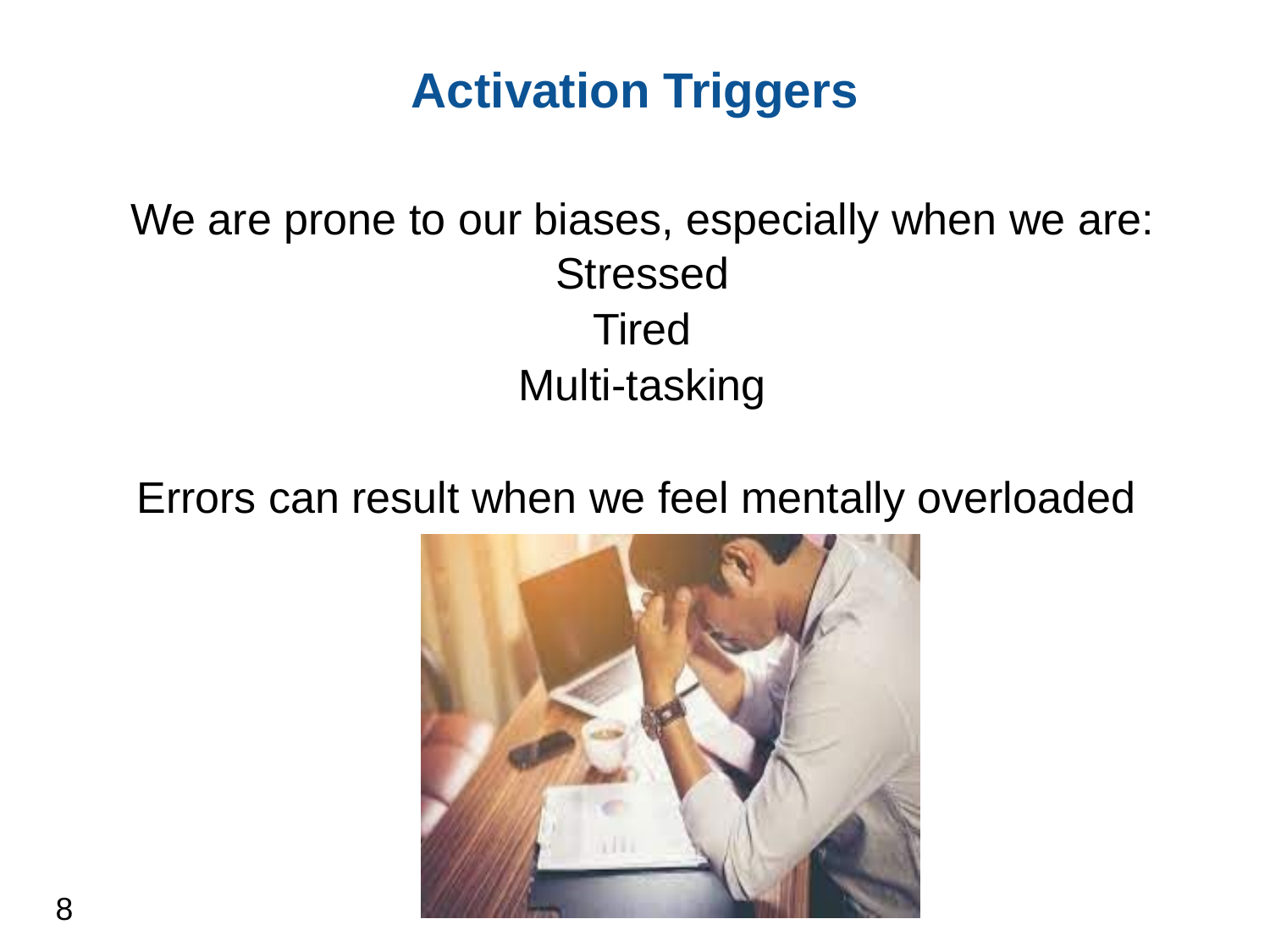

Activation Triggers
We are prone to our biases, especially when we are:
Stressed
Tired
Multi-tasking
Errors can result when we feel mentally overloaded
8

## Slide 9
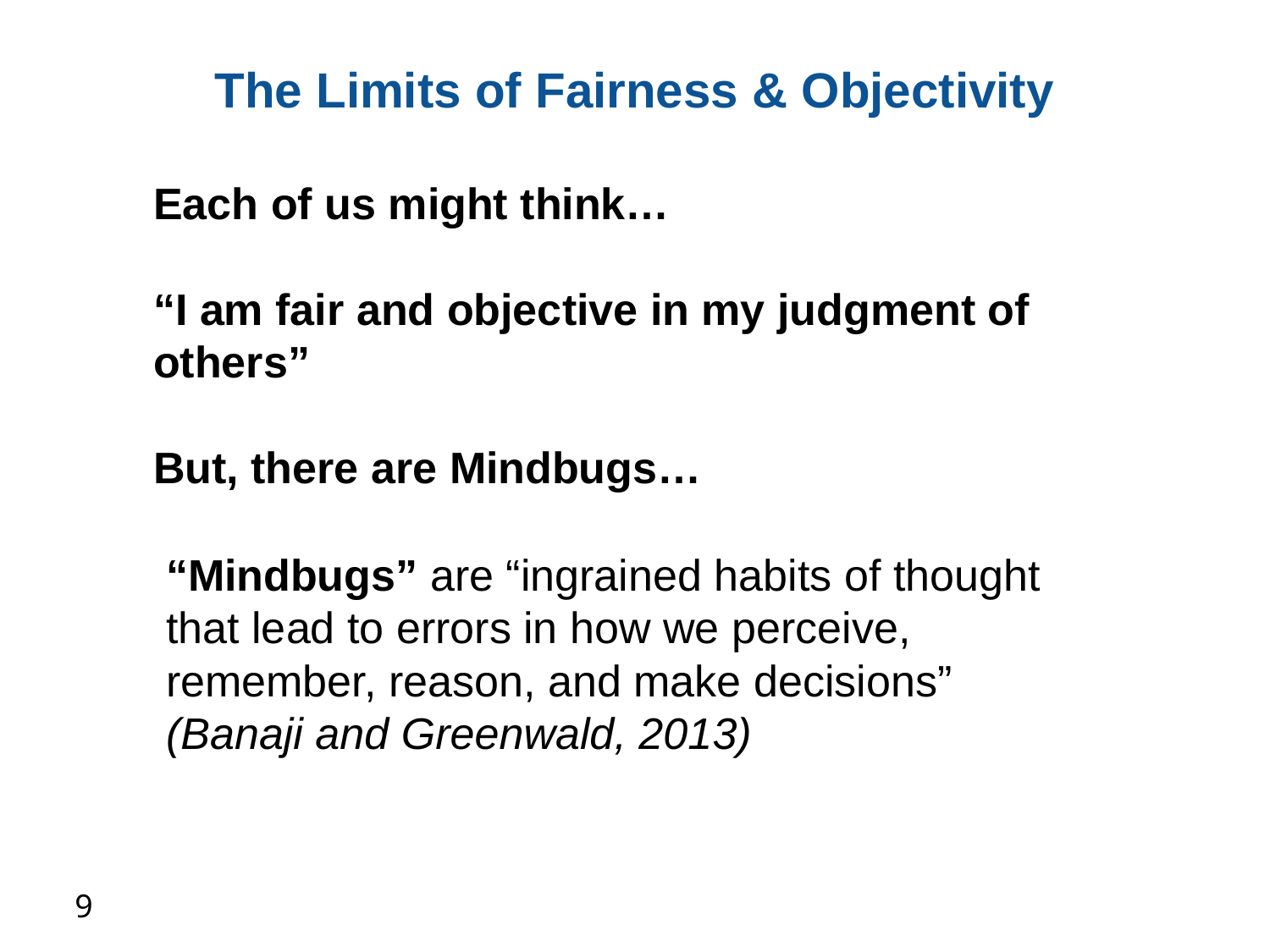

# The Limits of Fairness & Objectivity
Each of us might think…
“I am fair and objective in my judgment of others”
But, there are Mindbugs…
“Mindbugs” are “ingrained habits of thought that lead to errors in how we perceive, remember, reason, and make decisions” (Banaji and Greenwald, 2013)
9

## Slide 10
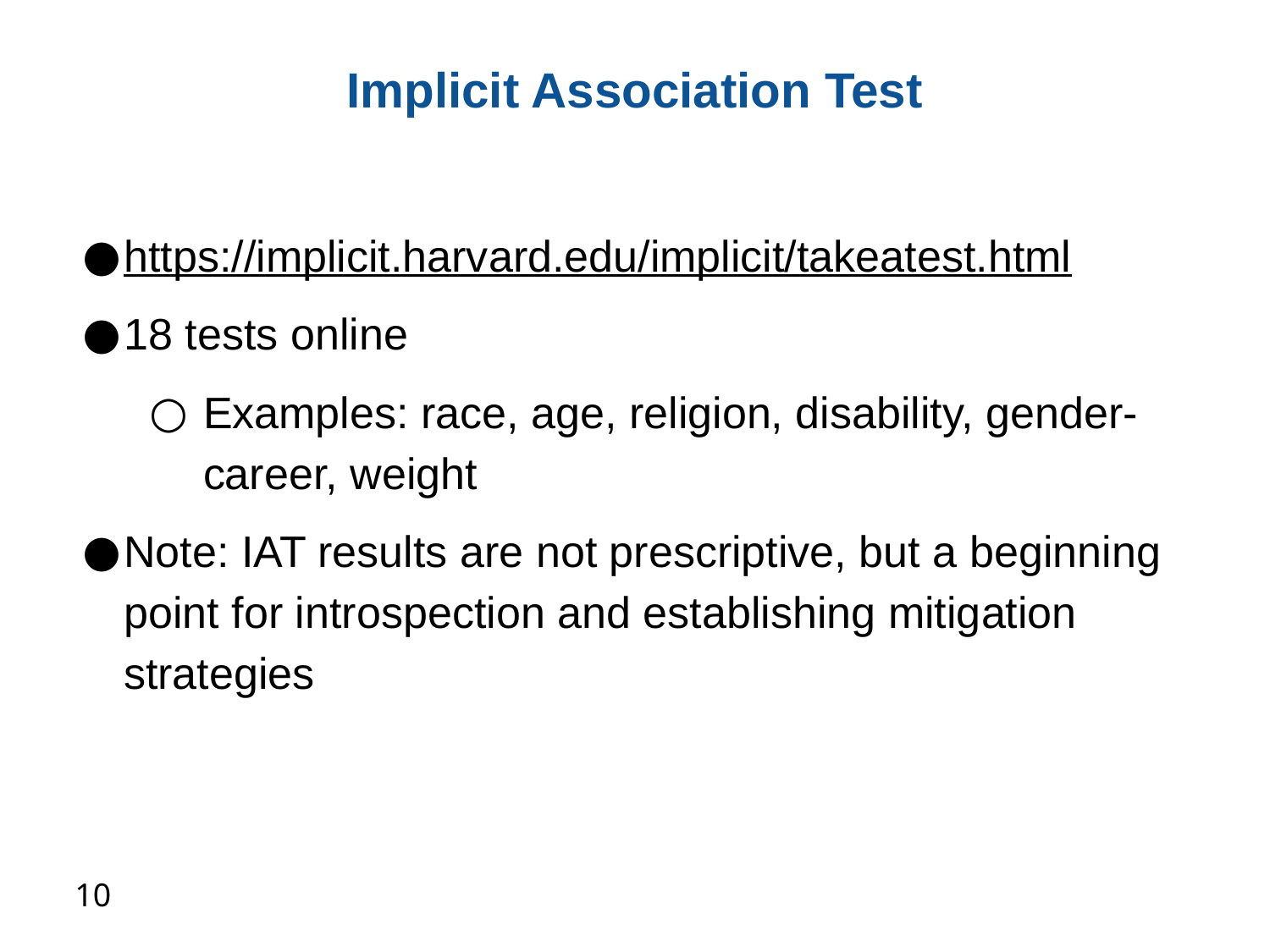

# Implicit Association Test
https://implicit.harvard.edu/implicit/takeatest.html
18 tests online
Examples: race, age, religion, disability, gender-career, weight
Note: IAT results are not prescriptive, but a beginning point for introspection and establishing mitigation strategies
10

## Slide 11
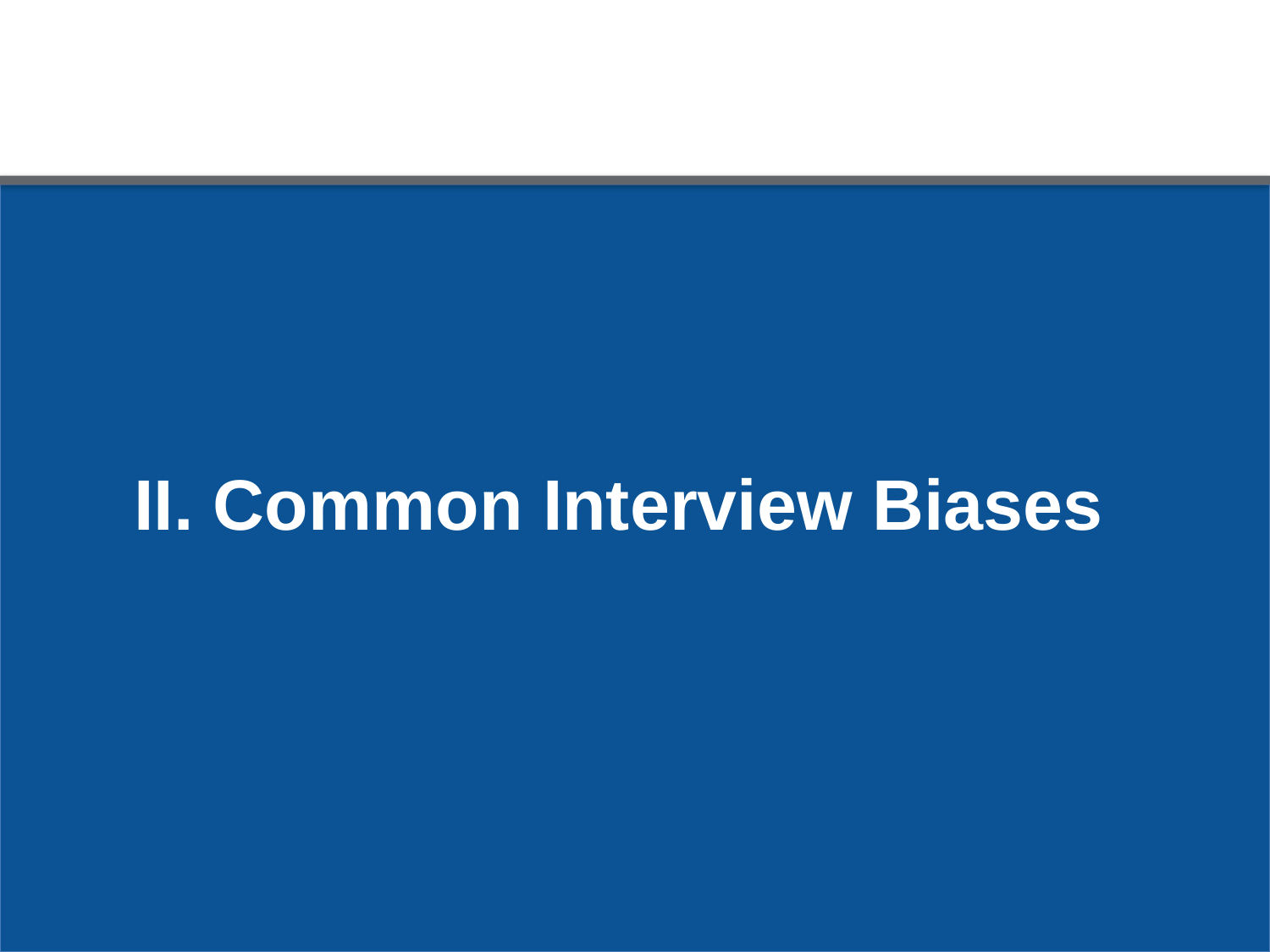

II. Common Interview Biases

## Slide 12
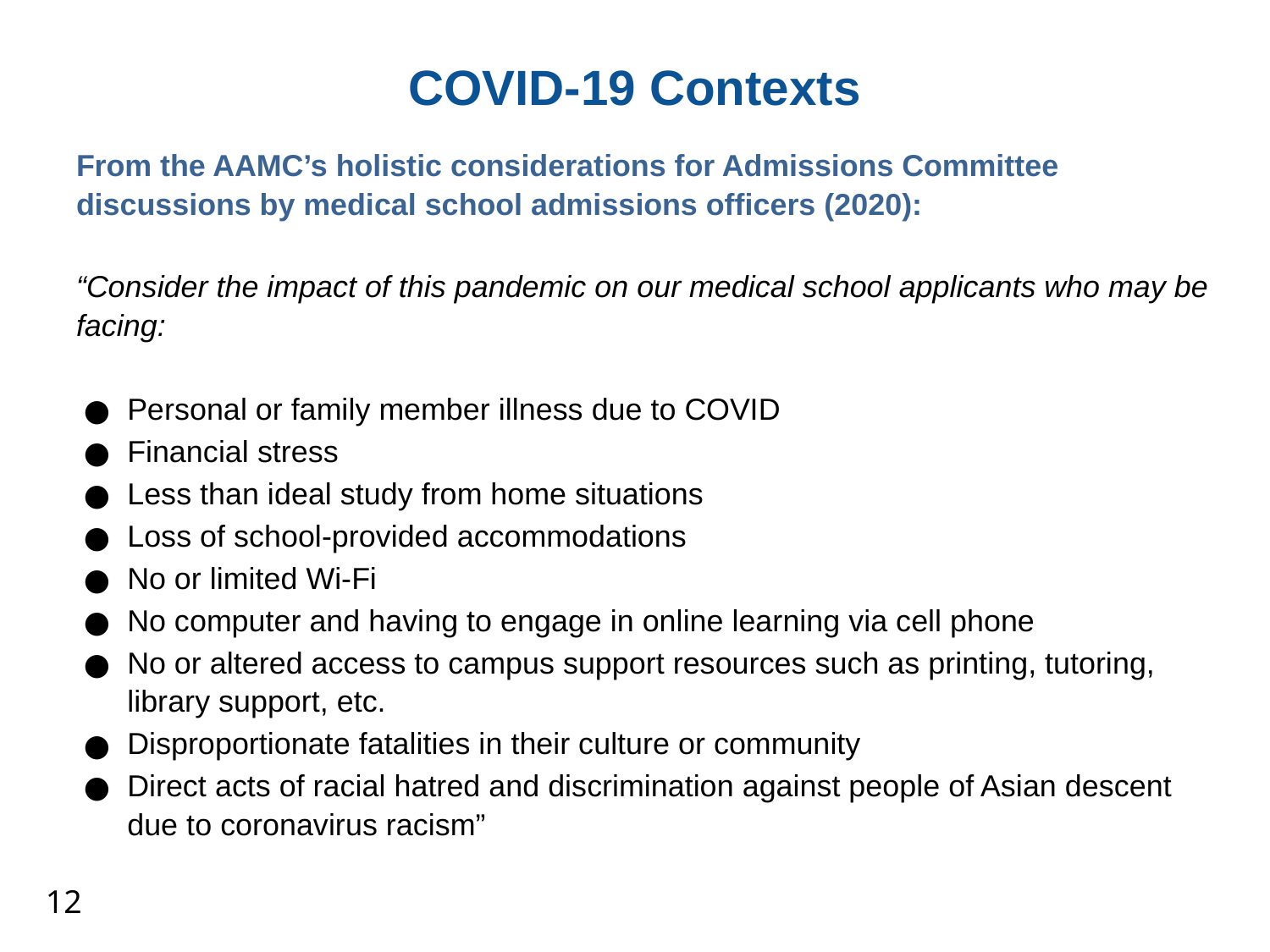

# COVID-19 Contexts
From the AAMC’s holistic considerations for Admissions Committee discussions by medical school admissions officers (2020):
“Consider the impact of this pandemic on our medical school applicants who may be facing:
Personal or family member illness due to COVID
Financial stress
Less than ideal study from home situations
Loss of school-provided accommodations
No or limited Wi-Fi
No computer and having to engage in online learning via cell phone
No or altered access to campus support resources such as printing, tutoring, library support, etc.
Disproportionate fatalities in their culture or community
Direct acts of racial hatred and discrimination against people of Asian descent due to coronavirus racism”
12

## Slide 13
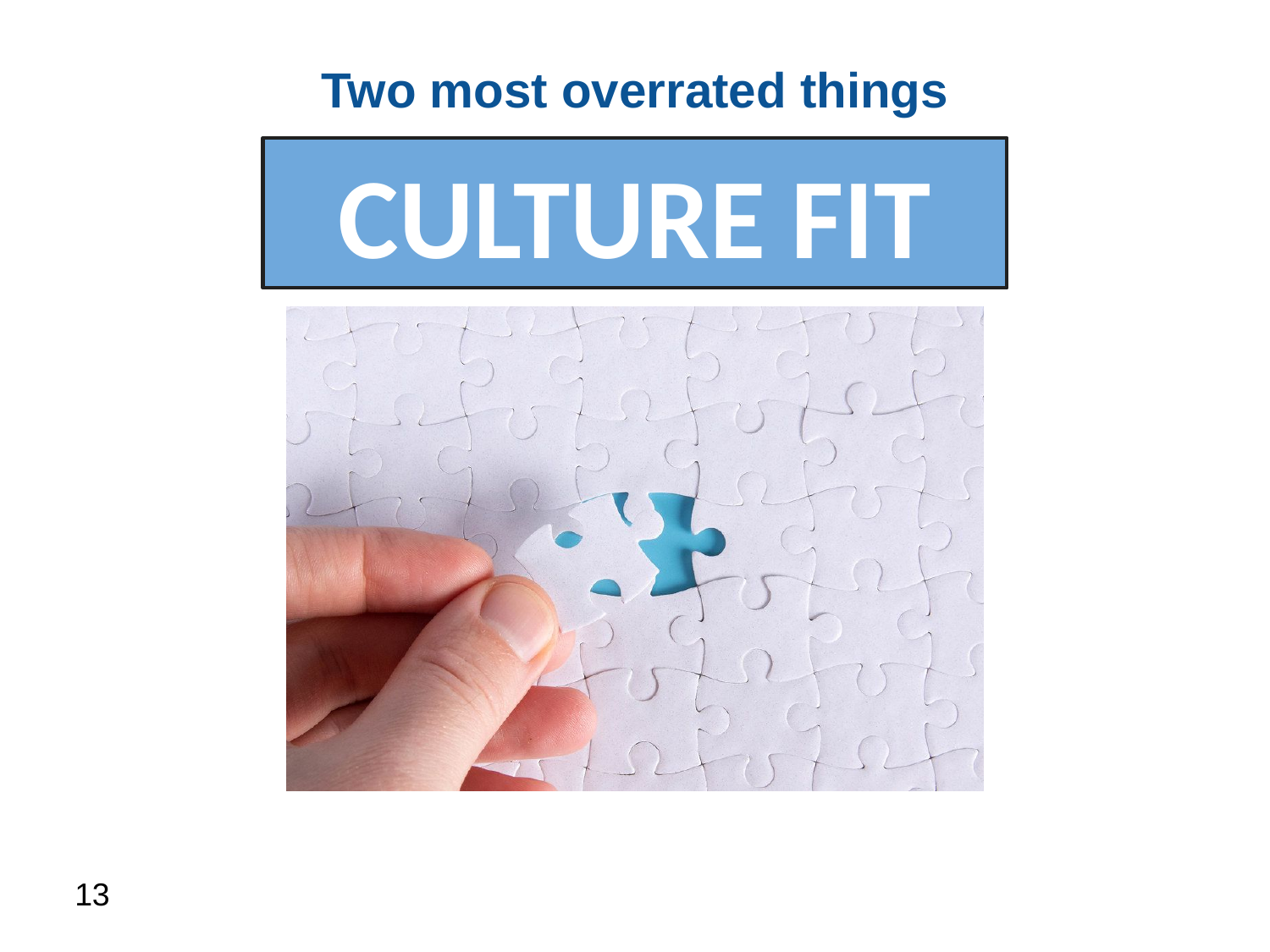

# Two most overrated things
CULTURE FIT
13

## Slide 14
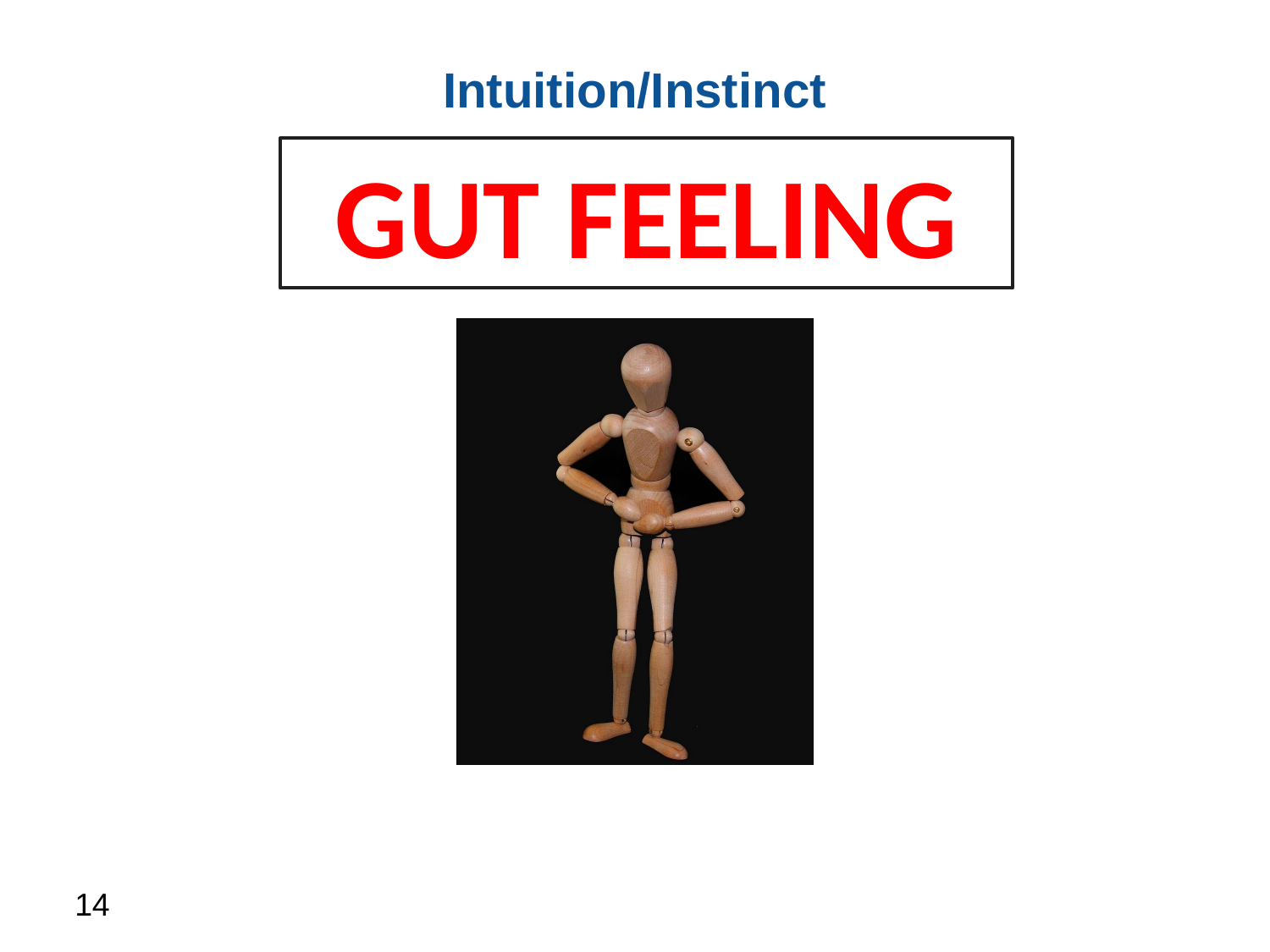

# Intuition/Instinct
GUT FEELING
14

## Slide 15
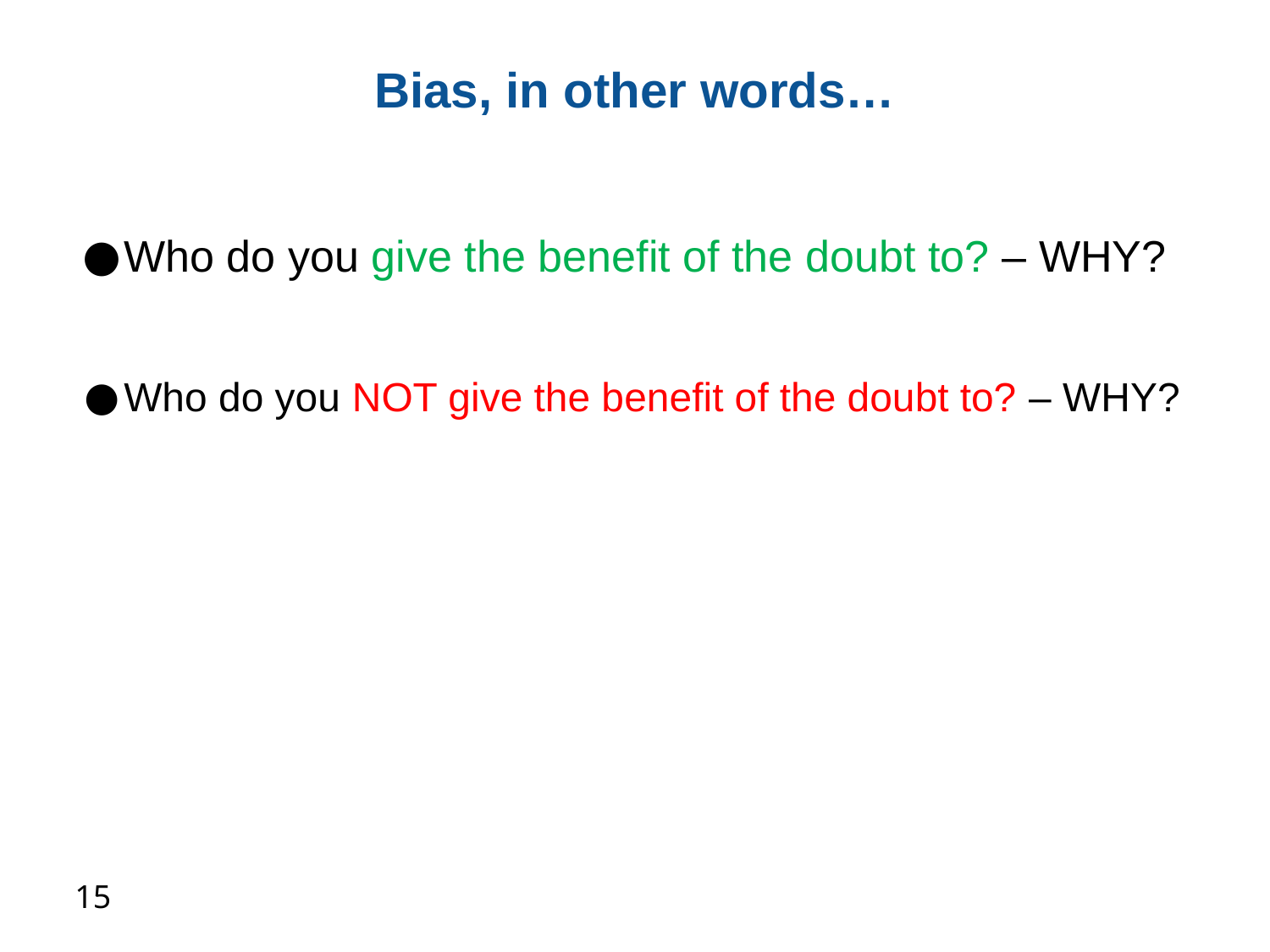

# Bias, in other words…
Who do you give the benefit of the doubt to? – WHY?
Who do you NOT give the benefit of the doubt to? – WHY?
15

## Slide 16
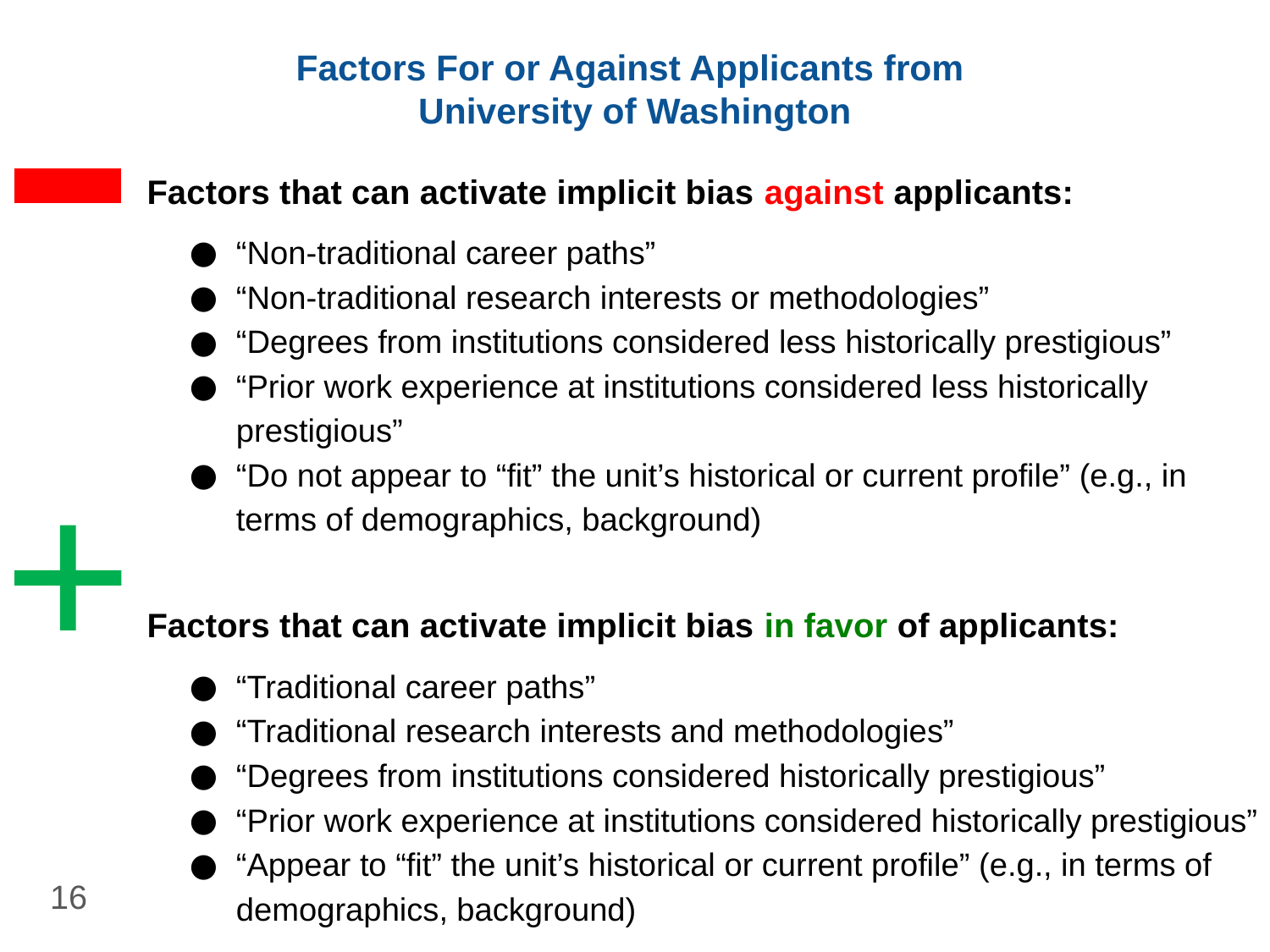

# Factors For or Against Applicants from
University of Washington
Factors that can activate implicit bias against applicants:
“Non-traditional career paths”
“Non-traditional research interests or methodologies”
“Degrees from institutions considered less historically prestigious”
“Prior work experience at institutions considered less historically prestigious”
“Do not appear to “fit” the unit’s historical or current profile” (e.g., in terms of demographics, background)
Factors that can activate implicit bias in favor of applicants:
“Traditional career paths”
“Traditional research interests and methodologies”
“Degrees from institutions considered historically prestigious”
“Prior work experience at institutions considered historically prestigious”
“Appear to “fit” the unit’s historical or current profile” (e.g., in terms of demographics, background)
-
+
16

## Slide 17
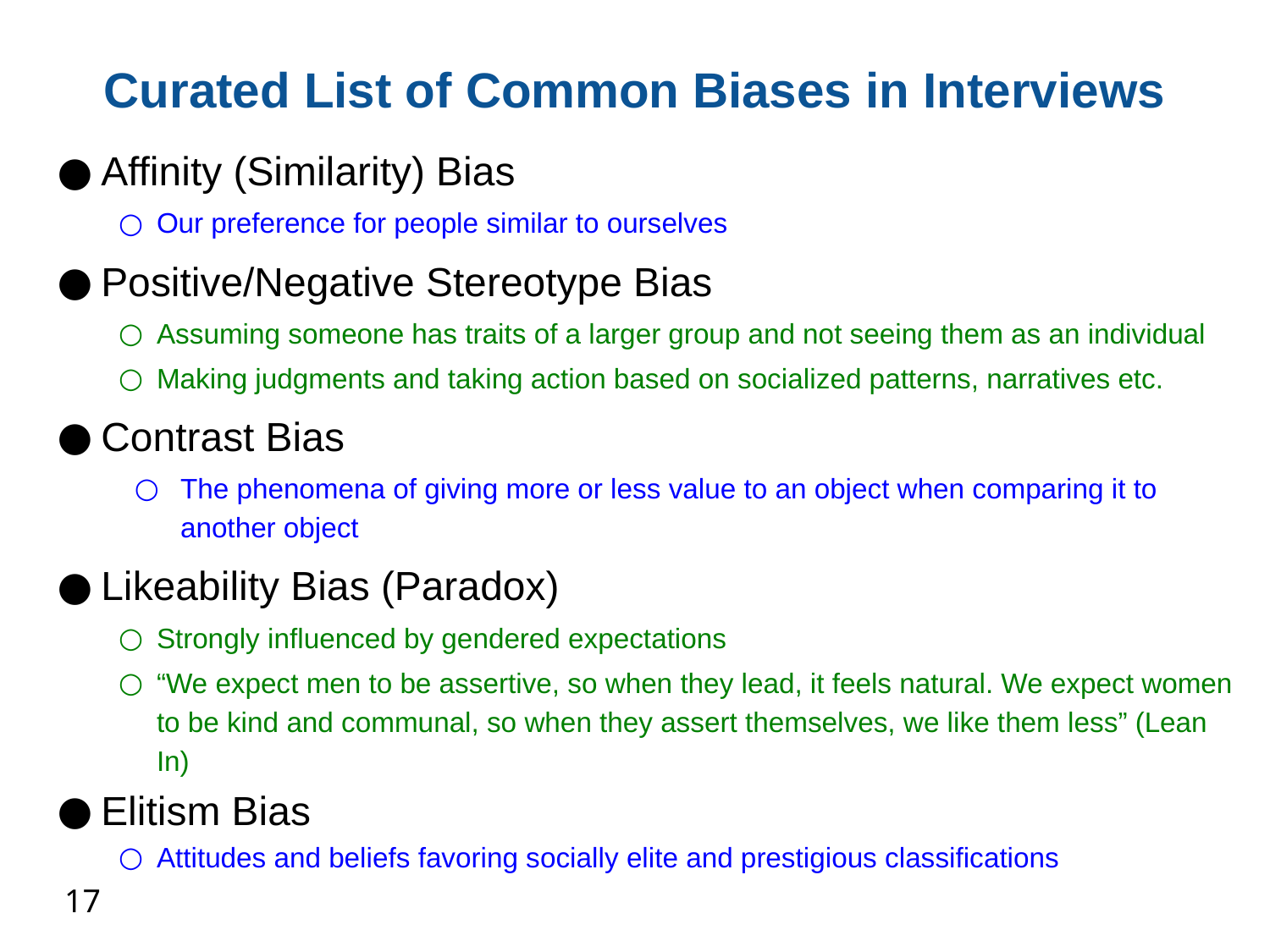

# Curated List of Common Biases in Interviews
Affinity (Similarity) Bias
Our preference for people similar to ourselves
Positive/Negative Stereotype Bias
Assuming someone has traits of a larger group and not seeing them as an individual
Making judgments and taking action based on socialized patterns, narratives etc.
Contrast Bias
The phenomena of giving more or less value to an object when comparing it to another object
Likeability Bias (Paradox)
Strongly influenced by gendered expectations
“We expect men to be assertive, so when they lead, it feels natural. We expect women to be kind and communal, so when they assert themselves, we like them less” (Lean In)
Elitism Bias
Attitudes and beliefs favoring socially elite and prestigious classifications
17

## Slide 18
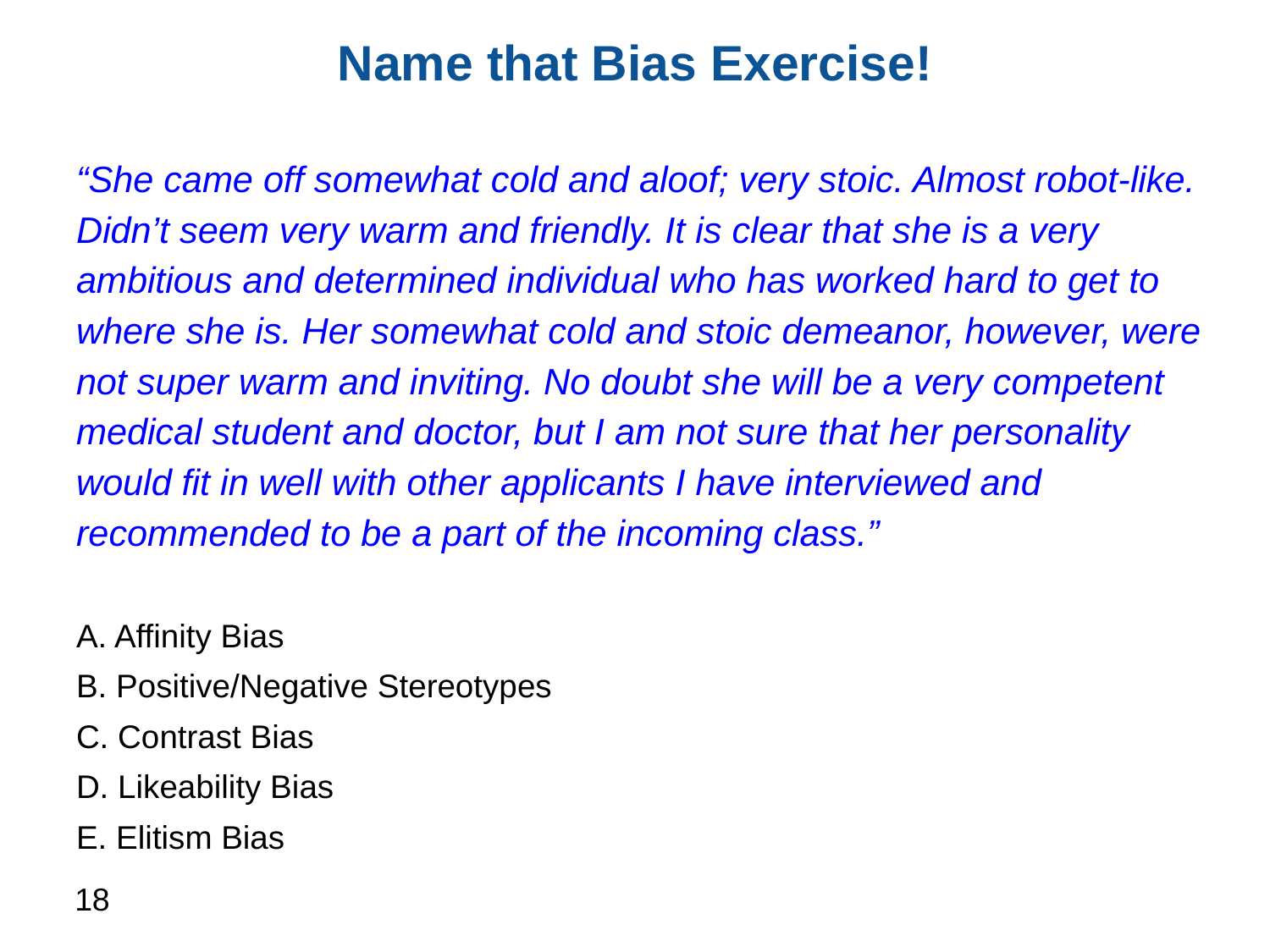

# Name that Bias Exercise!
“She came off somewhat cold and aloof; very stoic. Almost robot-like. Didn’t seem very warm and friendly. It is clear that she is a very ambitious and determined individual who has worked hard to get to where she is. Her somewhat cold and stoic demeanor, however, were not super warm and inviting. No doubt she will be a very competent medical student and doctor, but I am not sure that her personality would fit in well with other applicants I have interviewed and recommended to be a part of the incoming class.”
A. Affinity Bias
B. Positive/Negative Stereotypes
C. Contrast Bias
D. Likeability Bias
E. Elitism Bias
18

## Slide 19
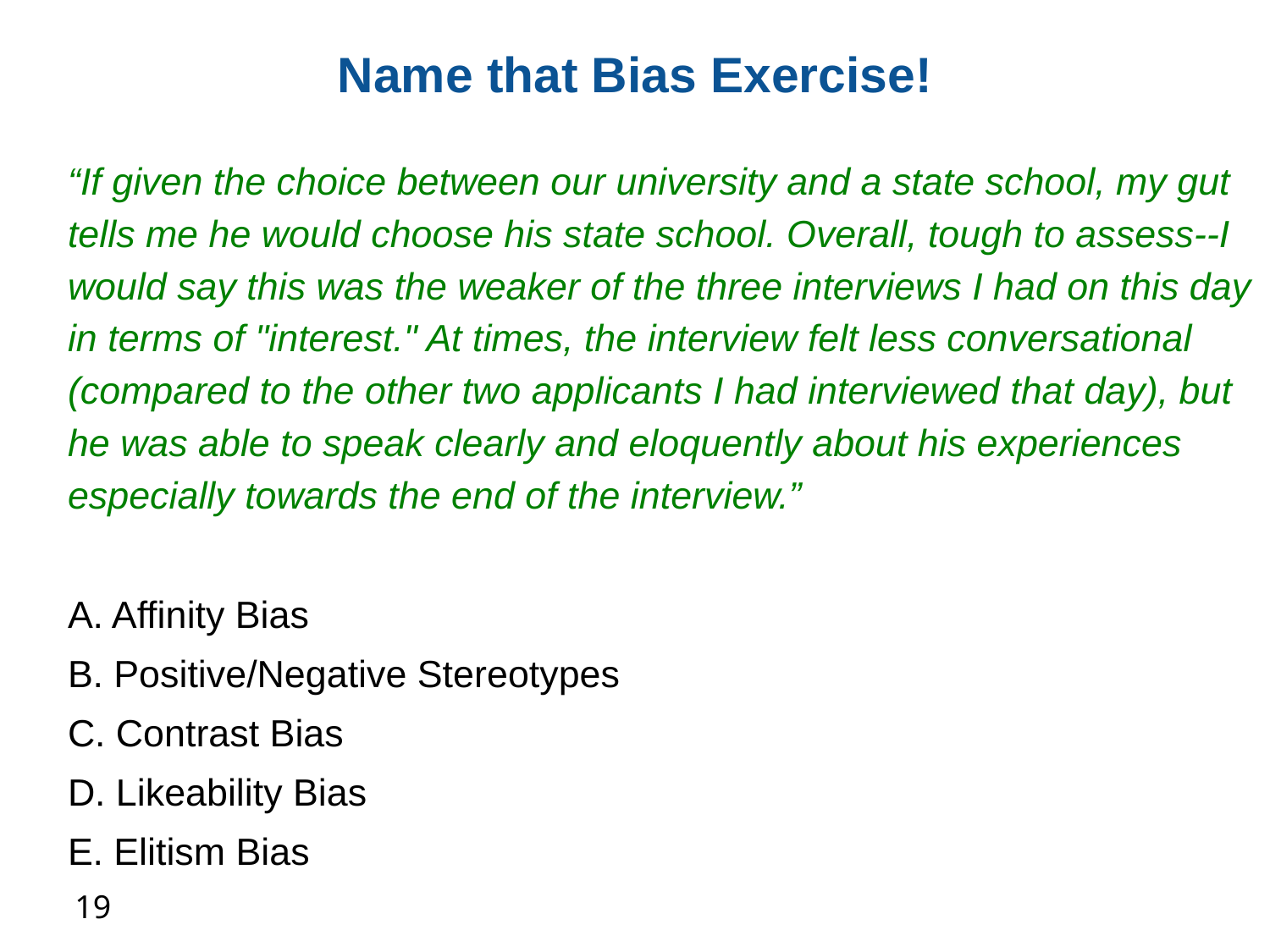

# Name that Bias Exercise!
“If given the choice between our university and a state school, my gut tells me he would choose his state school. Overall, tough to assess--I would say this was the weaker of the three interviews I had on this day in terms of "interest." At times, the interview felt less conversational (compared to the other two applicants I had interviewed that day), but he was able to speak clearly and eloquently about his experiences especially towards the end of the interview.”
A. Affinity Bias
B. Positive/Negative Stereotypes
C. Contrast Bias
D. Likeability Bias
E. Elitism Bias
19

## Slide 20
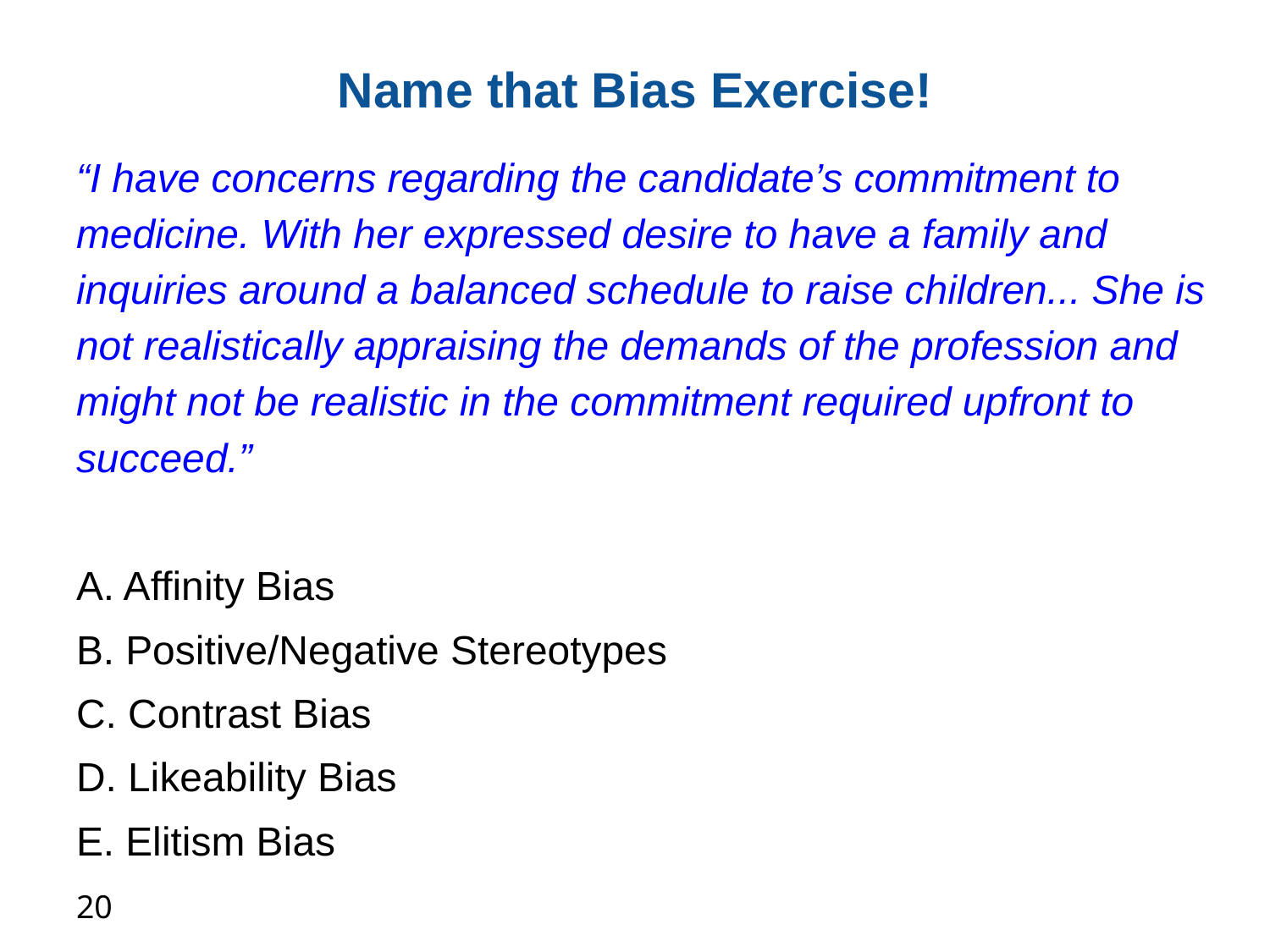

# Name that Bias Exercise!
“I have concerns regarding the candidate’s commitment to medicine. With her expressed desire to have a family and inquiries around a balanced schedule to raise children... She is not realistically appraising the demands of the profession and might not be realistic in the commitment required upfront to succeed.”
A. Affinity Bias
B. Positive/Negative Stereotypes
C. Contrast Bias
D. Likeability Bias
E. Elitism Bias
20

## Slide 21
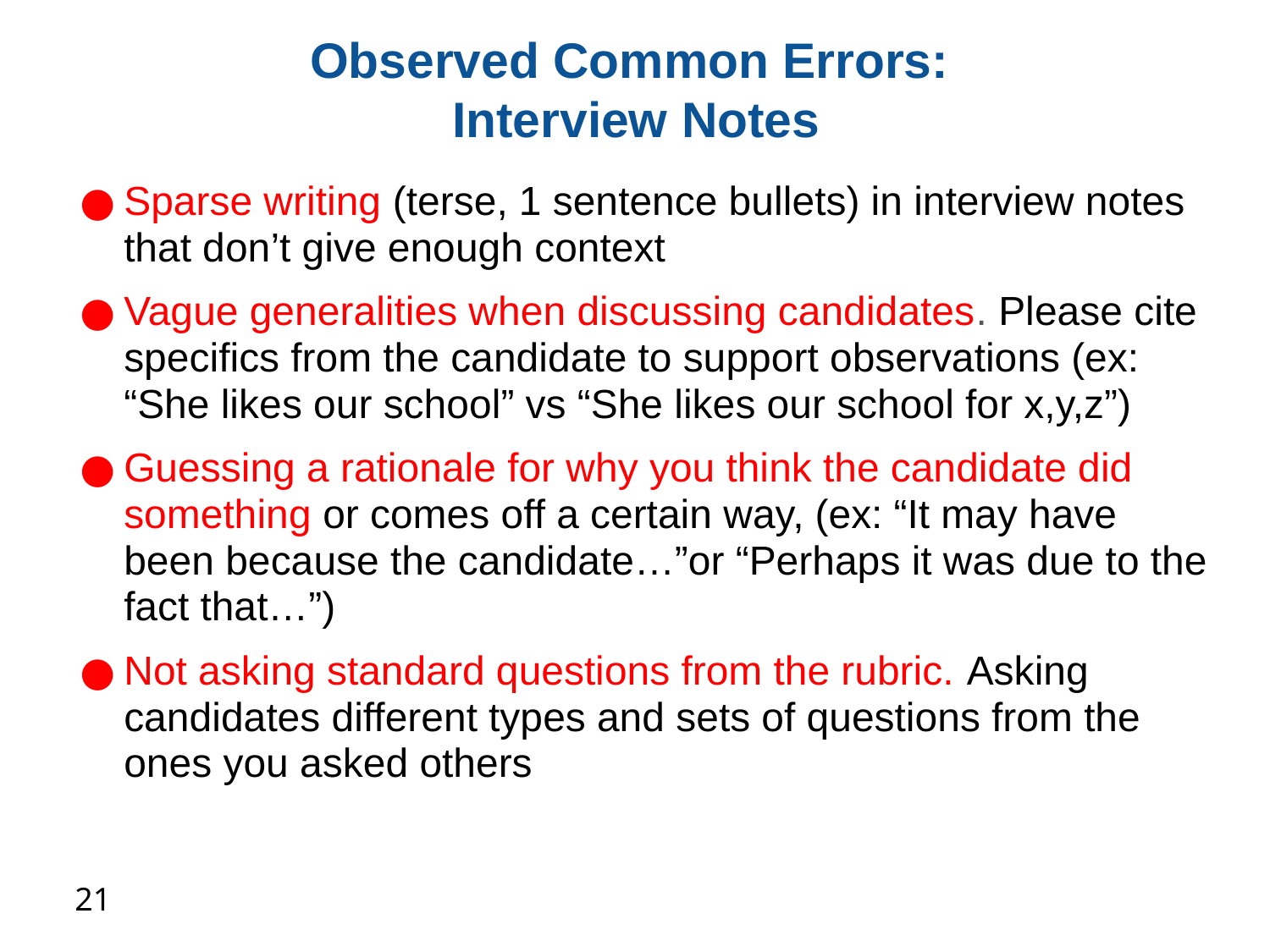

# Observed Common Errors: Interview Notes
Sparse writing (terse, 1 sentence bullets) in interview notes that don’t give enough context
Vague generalities when discussing candidates. Please cite specifics from the candidate to support observations (ex: “She likes our school” vs “She likes our school for x,y,z”)
Guessing a rationale for why you think the candidate did something or comes off a certain way, (ex: “It may have been because the candidate…”or “Perhaps it was due to the fact that…”)
Not asking standard questions from the rubric. Asking candidates different types and sets of questions from the ones you asked others
21

## Slide 22
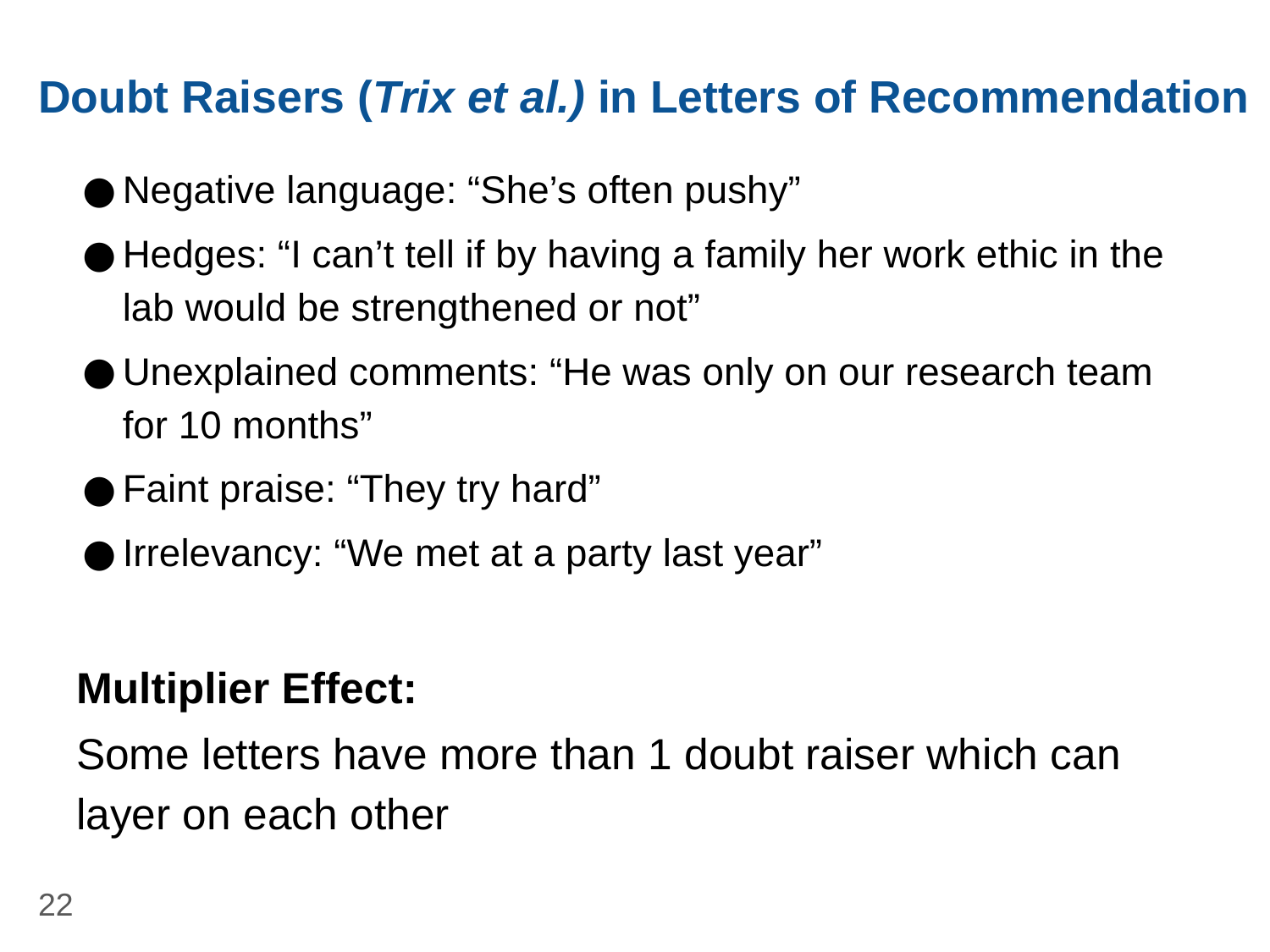

# Doubt Raisers (Trix et al.) in Letters of Recommendation
Negative language: “She’s often pushy”
Hedges: “I can’t tell if by having a family her work ethic in the lab would be strengthened or not”
Unexplained comments: “He was only on our research team for 10 months”
Faint praise: “They try hard”
Irrelevancy: “We met at a party last year”
Multiplier Effect:
Some letters have more than 1 doubt raiser which can layer on each other
22

## Slide 23
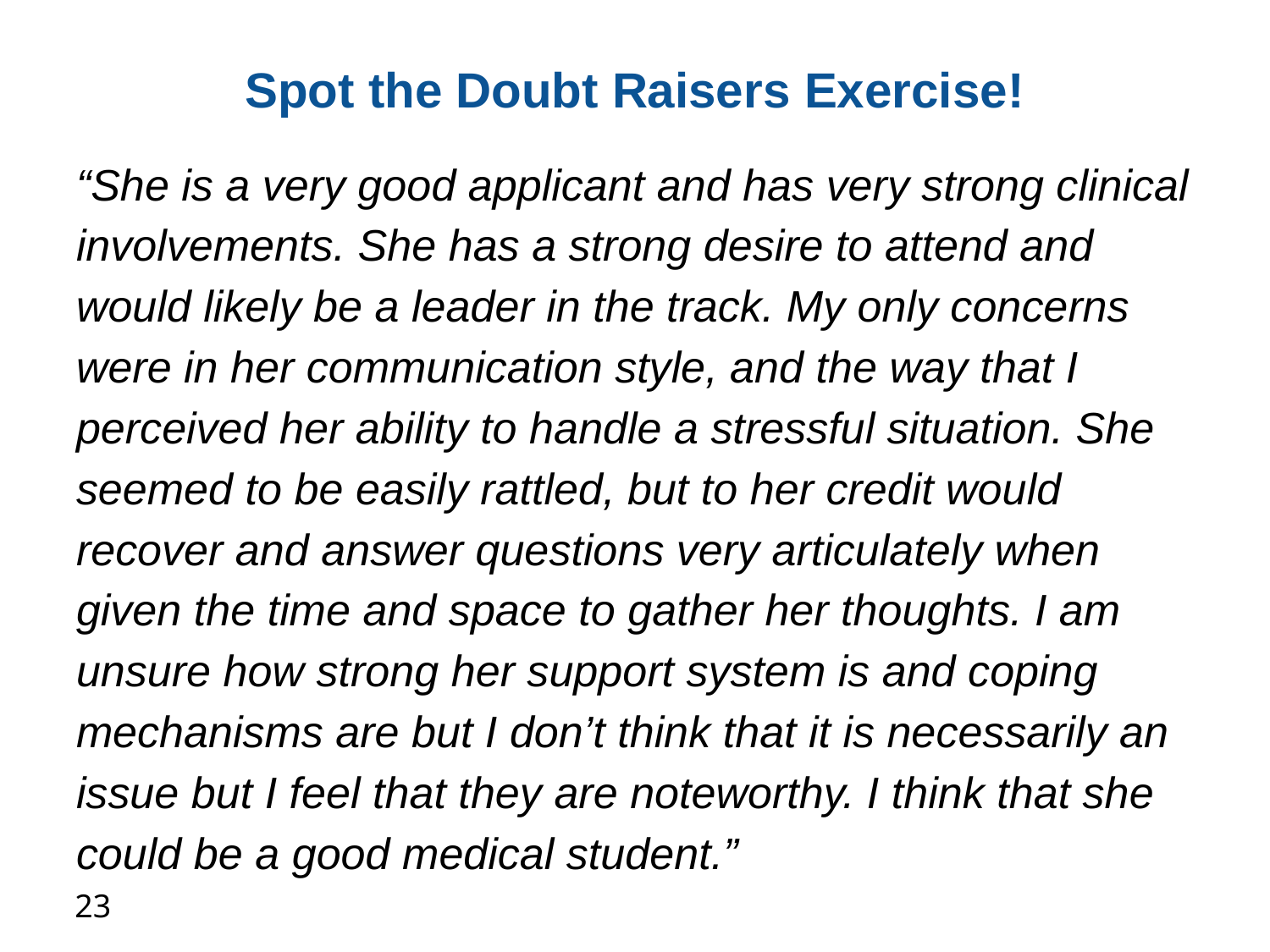

# Spot the Doubt Raisers Exercise!
“She is a very good applicant and has very strong clinical involvements. She has a strong desire to attend and would likely be a leader in the track. My only concerns were in her communication style, and the way that I perceived her ability to handle a stressful situation. She seemed to be easily rattled, but to her credit would recover and answer questions very articulately when given the time and space to gather her thoughts. I am unsure how strong her support system is and coping mechanisms are but I don’t think that it is necessarily an issue but I feel that they are noteworthy. I think that she could be a good medical student.”
23

## Slide 24
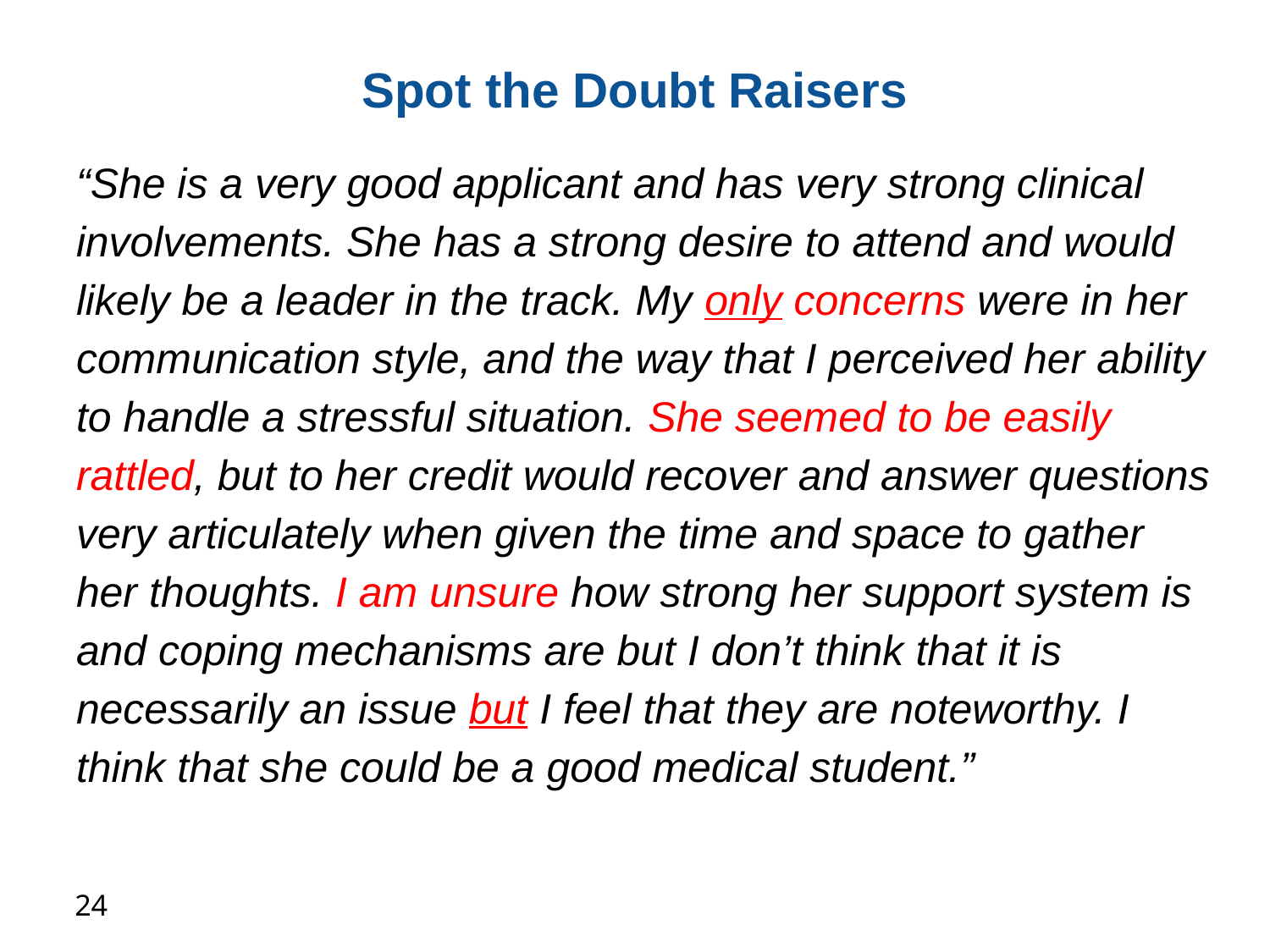

# Spot the Doubt Raisers
“She is a very good applicant and has very strong clinical involvements. She has a strong desire to attend and would likely be a leader in the track. My only concerns were in her communication style, and the way that I perceived her ability to handle a stressful situation. She seemed to be easily rattled, but to her credit would recover and answer questions very articulately when given the time and space to gather her thoughts. I am unsure how strong her support system is and coping mechanisms are but I don’t think that it is necessarily an issue but I feel that they are noteworthy. I think that she could be a good medical student.”
24

## Slide 25
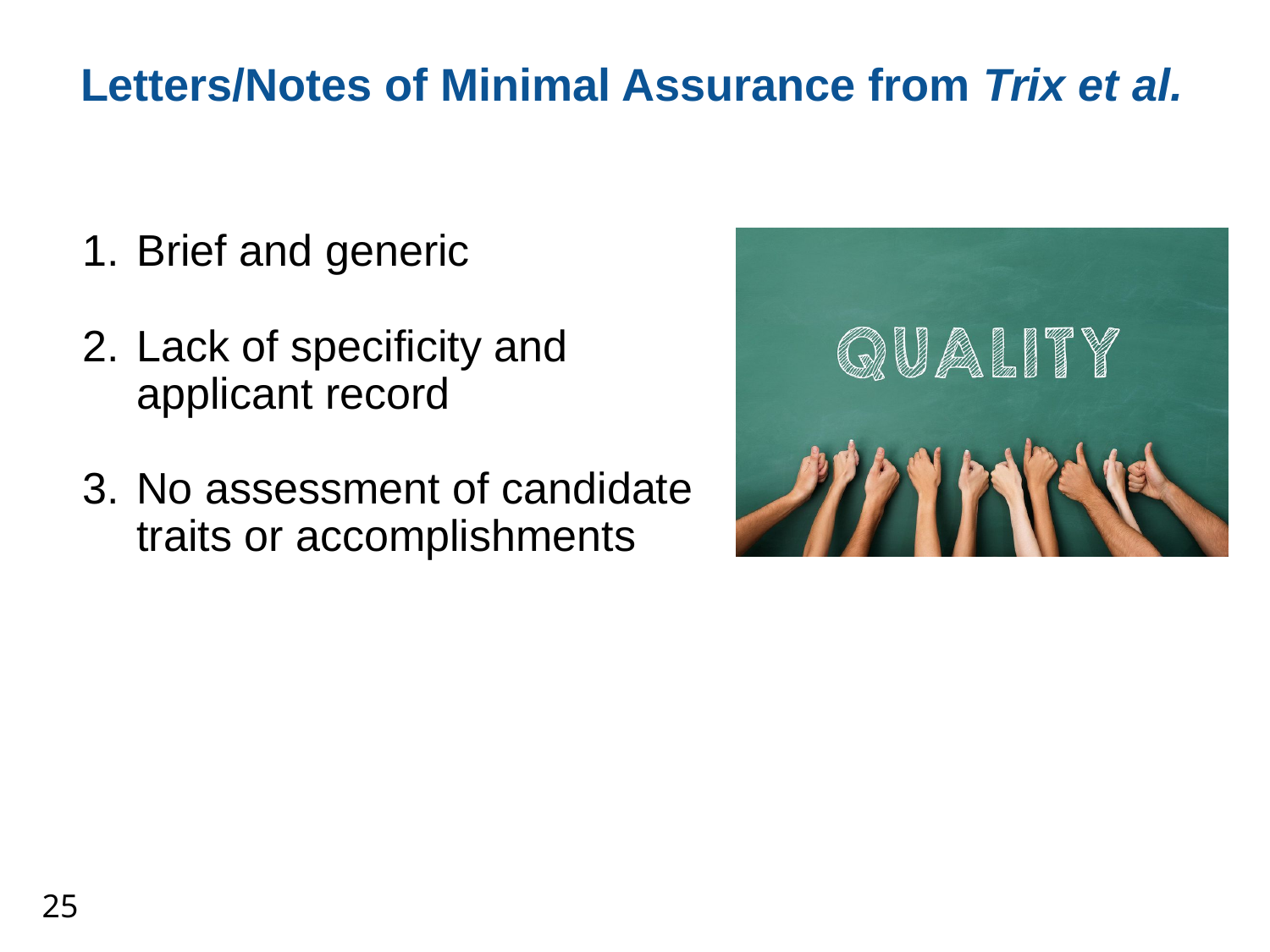

Letters/Notes of Minimal Assurance from Trix et al.
Brief and generic
Lack of specificity and applicant record
No assessment of candidate traits or accomplishments
25

## Slide 26
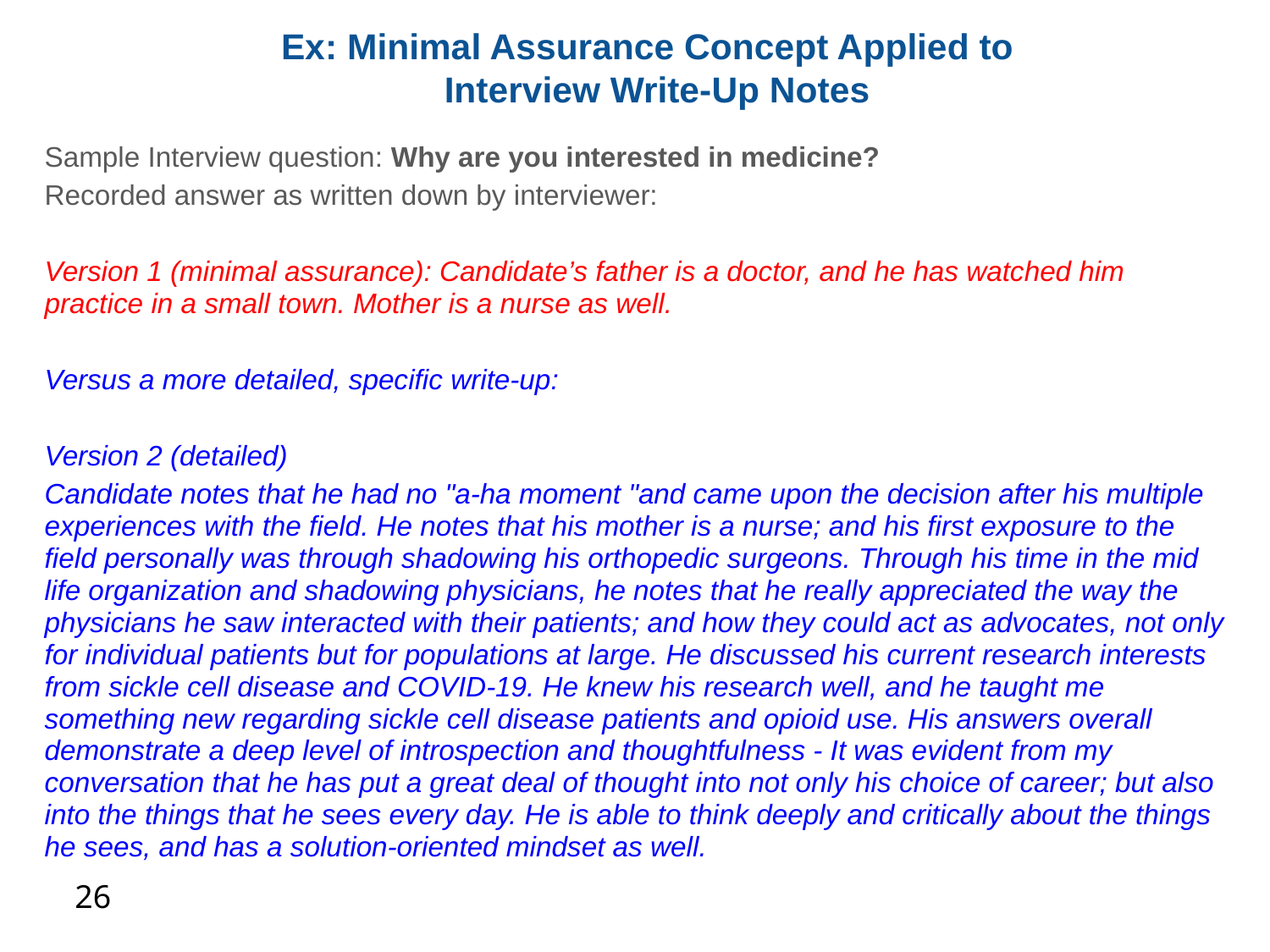

# Ex: Minimal Assurance Concept Applied to
 Interview Write-Up Notes
Sample Interview question: Why are you interested in medicine?
Recorded answer as written down by interviewer:
Version 1 (minimal assurance): Candidate’s father is a doctor, and he has watched him practice in a small town. Mother is a nurse as well.
Versus a more detailed, specific write-up:
Version 2 (detailed)
Candidate notes that he had no "a-ha moment "and came upon the decision after his multiple experiences with the field. He notes that his mother is a nurse; and his first exposure to the field personally was through shadowing his orthopedic surgeons. Through his time in the mid life organization and shadowing physicians, he notes that he really appreciated the way the physicians he saw interacted with their patients; and how they could act as advocates, not only for individual patients but for populations at large. He discussed his current research interests from sickle cell disease and COVID-19. He knew his research well, and he taught me something new regarding sickle cell disease patients and opioid use. His answers overall demonstrate a deep level of introspection and thoughtfulness - It was evident from my conversation that he has put a great deal of thought into not only his choice of career; but also into the things that he sees every day. He is able to think deeply and critically about the things he sees, and has a solution-oriented mindset as well.
26

## Slide 27
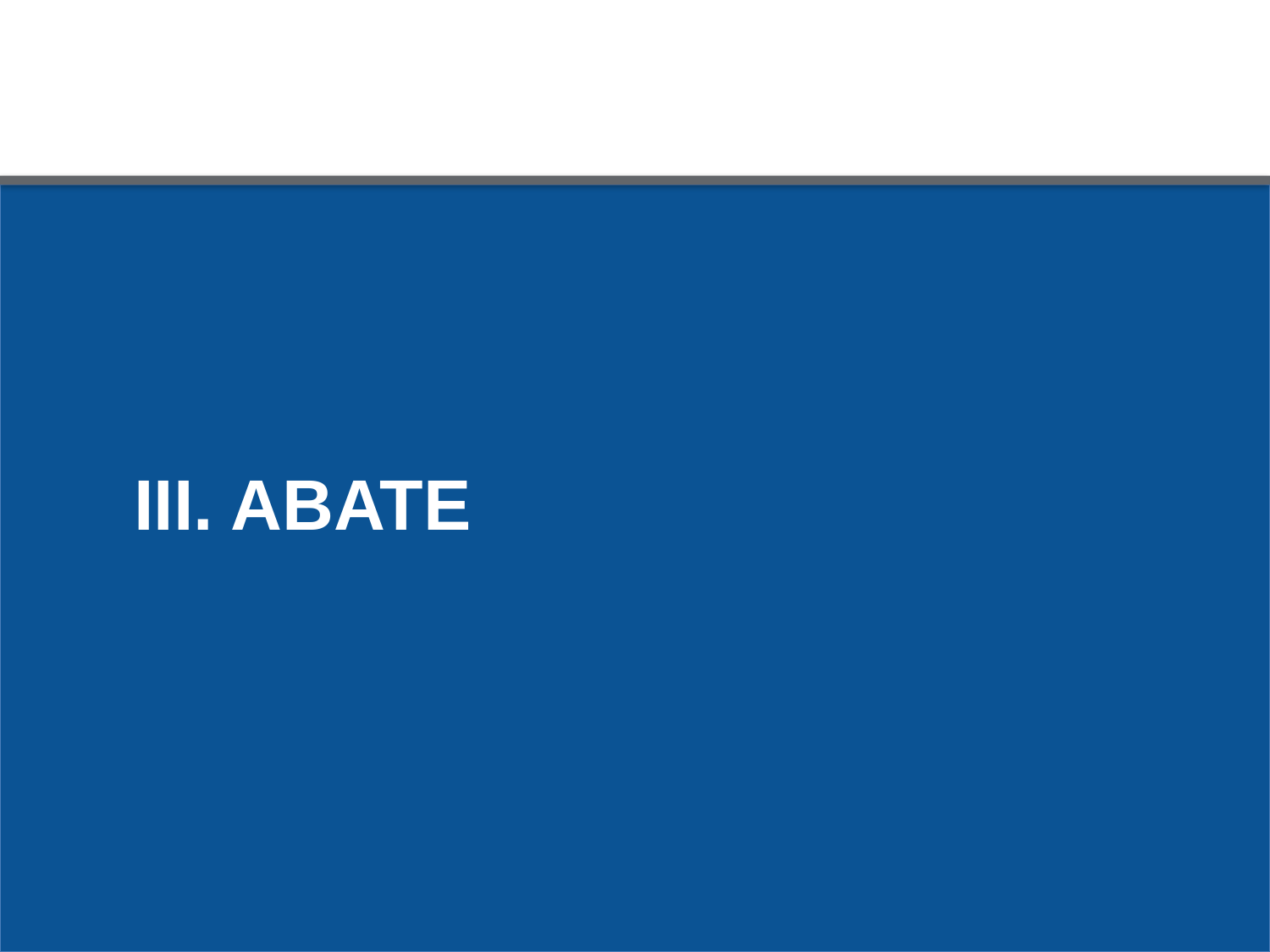

III. ABATE

## Slide 28
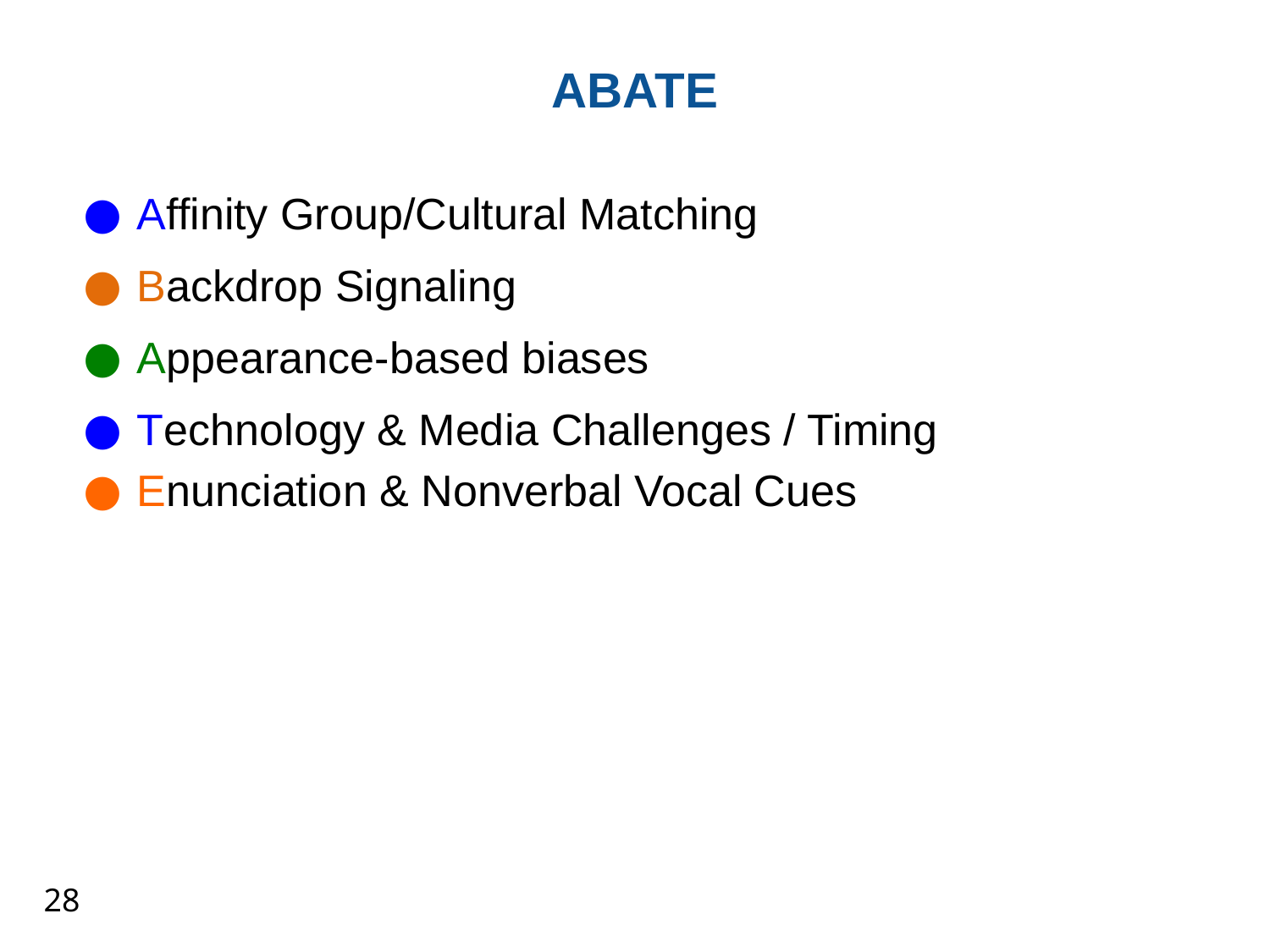

# ABATE
Affinity Group/Cultural Matching
Backdrop Signaling
Appearance-based biases
Technology & Media Challenges / Timing
Enunciation & Nonverbal Vocal Cues
28

## Slide 29
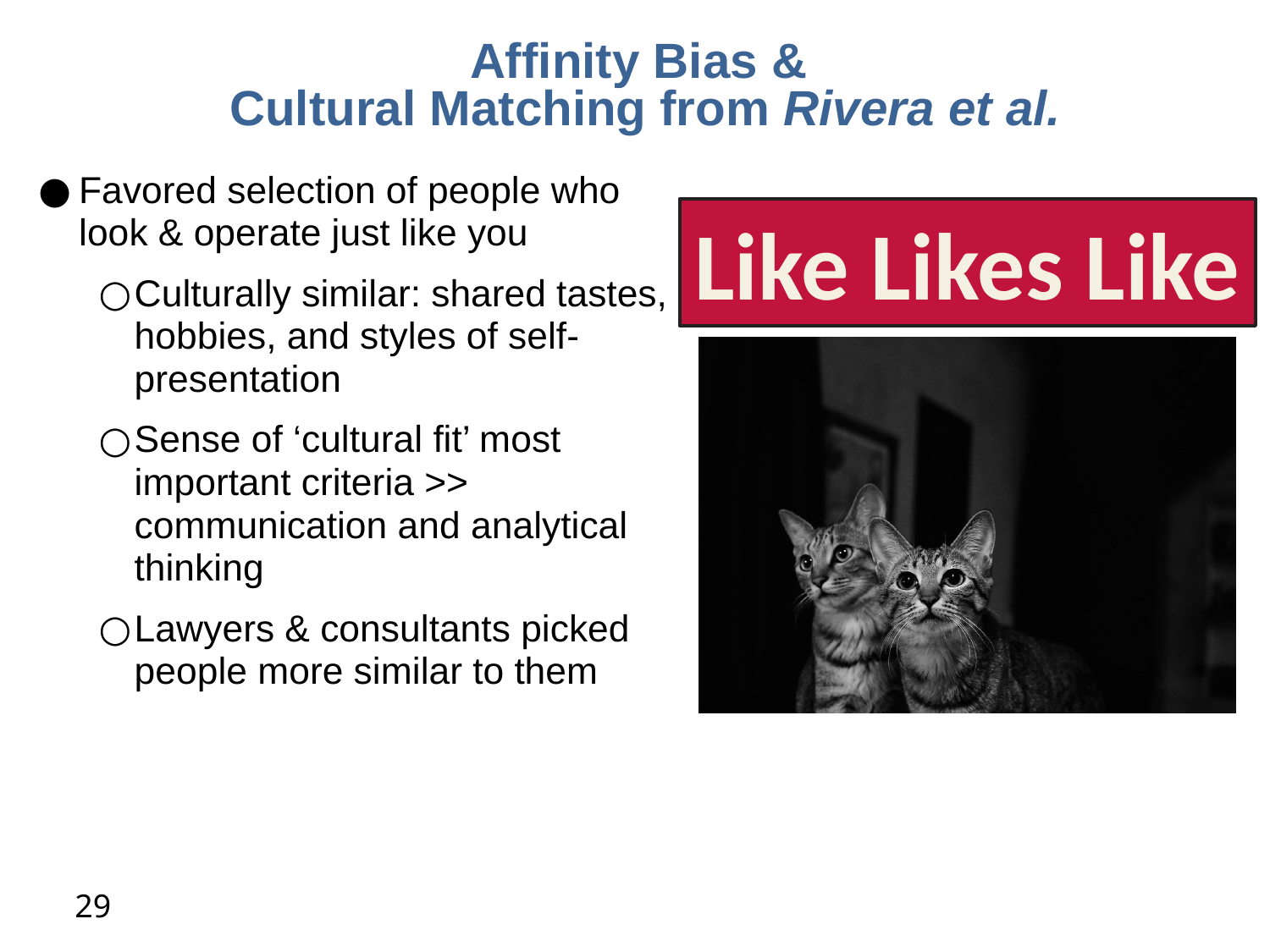

Affinity Bias &
Cultural Matching from Rivera et al.
Favored selection of people who look & operate just like you
Culturally similar: shared tastes, hobbies, and styles of self-presentation
Sense of ‘cultural fit’ most important criteria >> communication and analytical thinking
Lawyers & consultants picked people more similar to them
Like Likes Like
29

## Slide 30
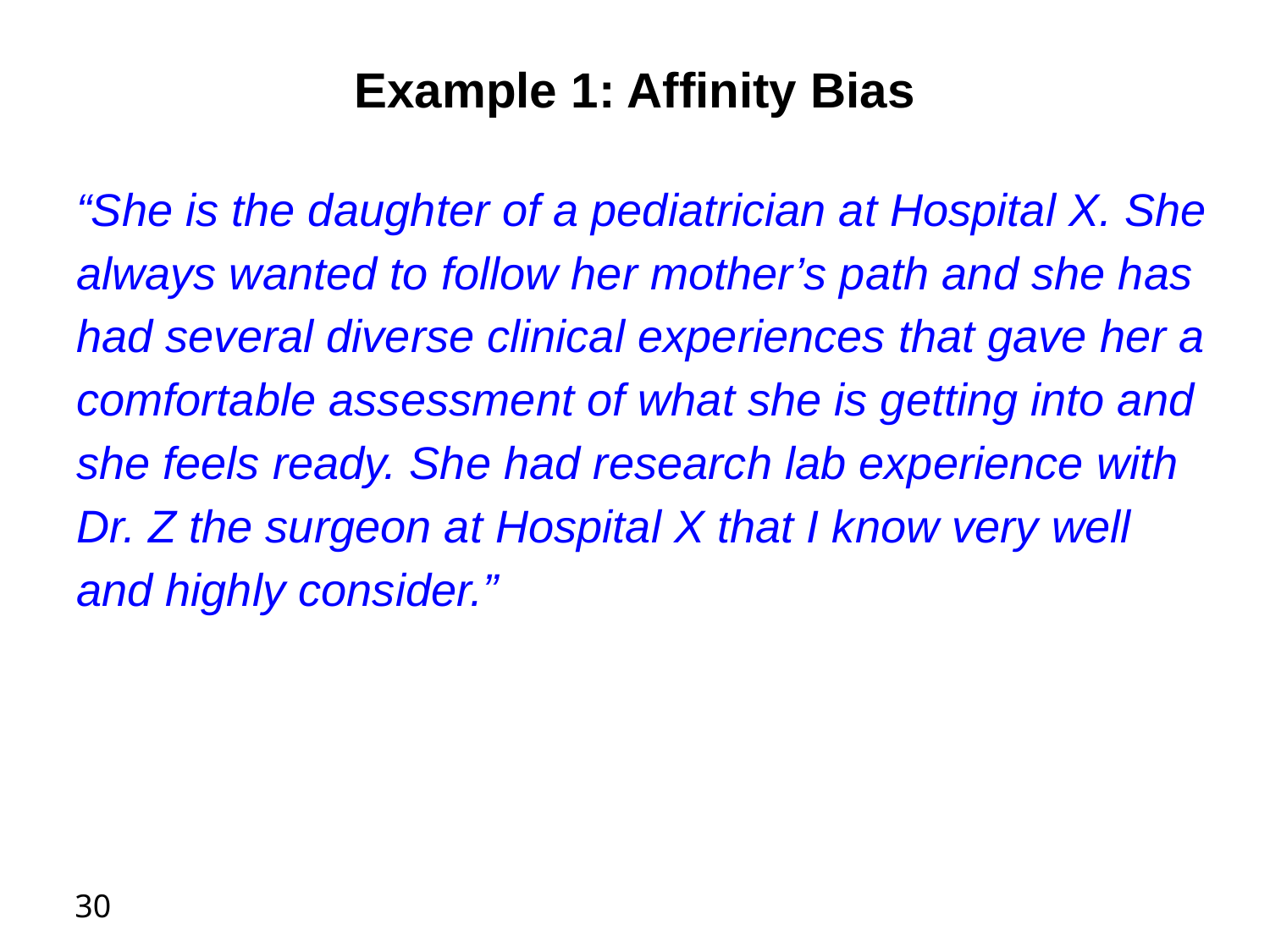

# Example 1: Affinity Bias
“She is the daughter of a pediatrician at Hospital X. She always wanted to follow her mother’s path and she has had several diverse clinical experiences that gave her a comfortable assessment of what she is getting into and she feels ready. She had research lab experience with Dr. Z the surgeon at Hospital X that I know very well and highly consider.”
30

## Slide 31
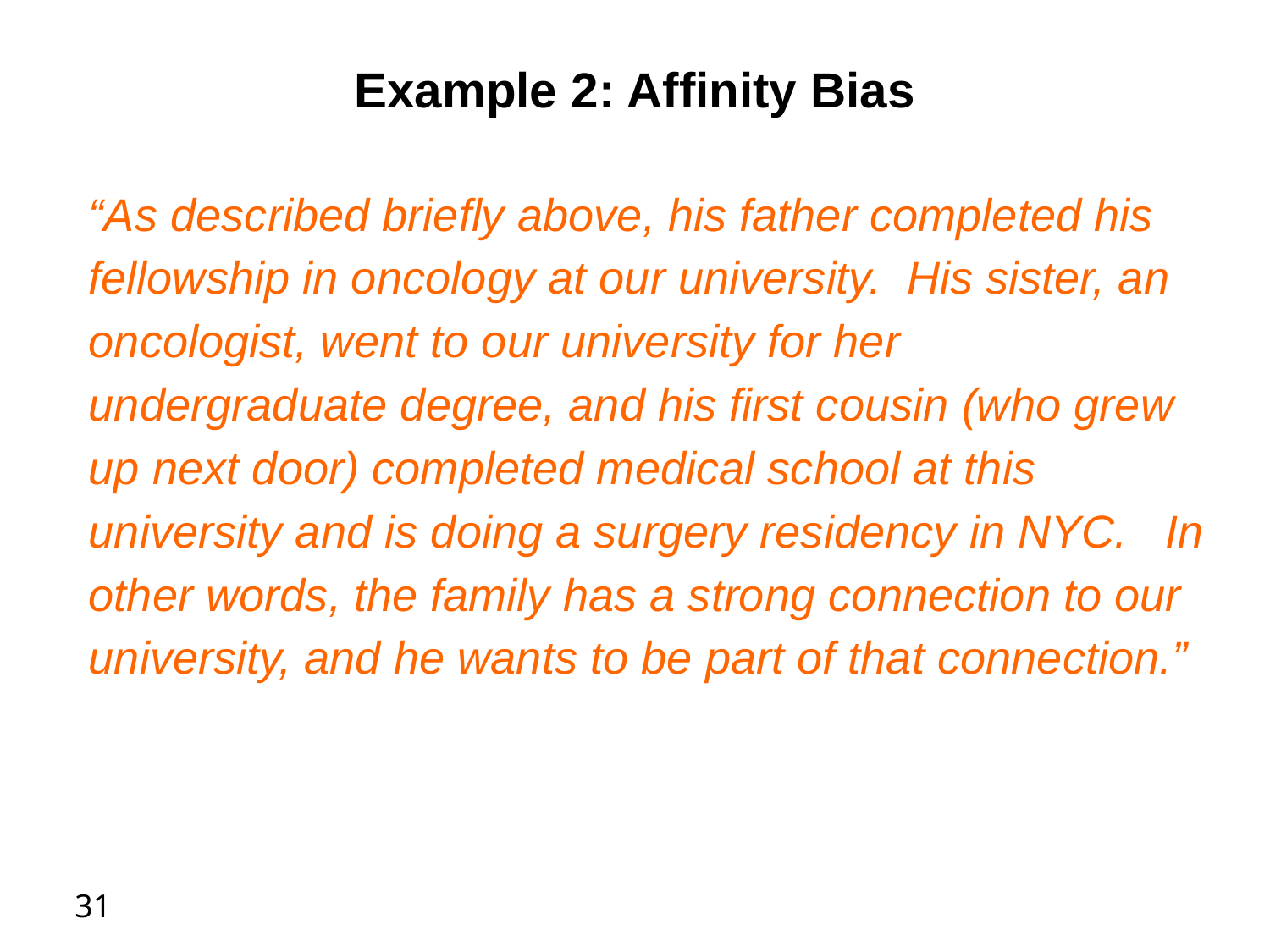

# Example 2: Affinity Bias
“As described briefly above, his father completed his fellowship in oncology at our university.  His sister, an oncologist, went to our university for her undergraduate degree, and his first cousin (who grew up next door) completed medical school at this university and is doing a surgery residency in NYC.   In other words, the family has a strong connection to our university, and he wants to be part of that connection.”
31

## Slide 32
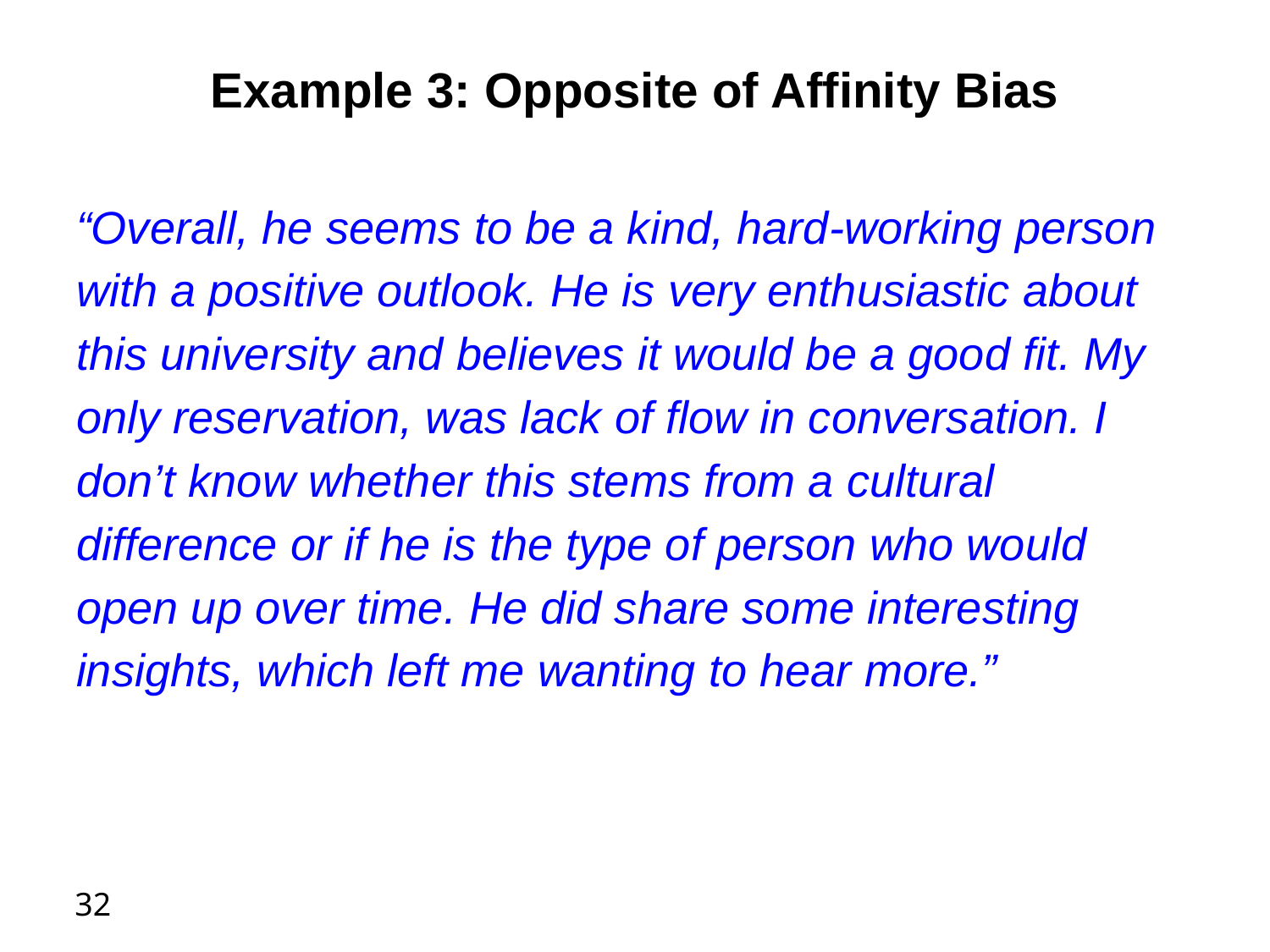

# Example 3: Opposite of Affinity Bias
“Overall, he seems to be a kind, hard-working person with a positive outlook. He is very enthusiastic about this university and believes it would be a good fit. My only reservation, was lack of flow in conversation. I don’t know whether this stems from a cultural difference or if he is the type of person who would open up over time. He did share some interesting insights, which left me wanting to hear more.”
32

## Slide 33
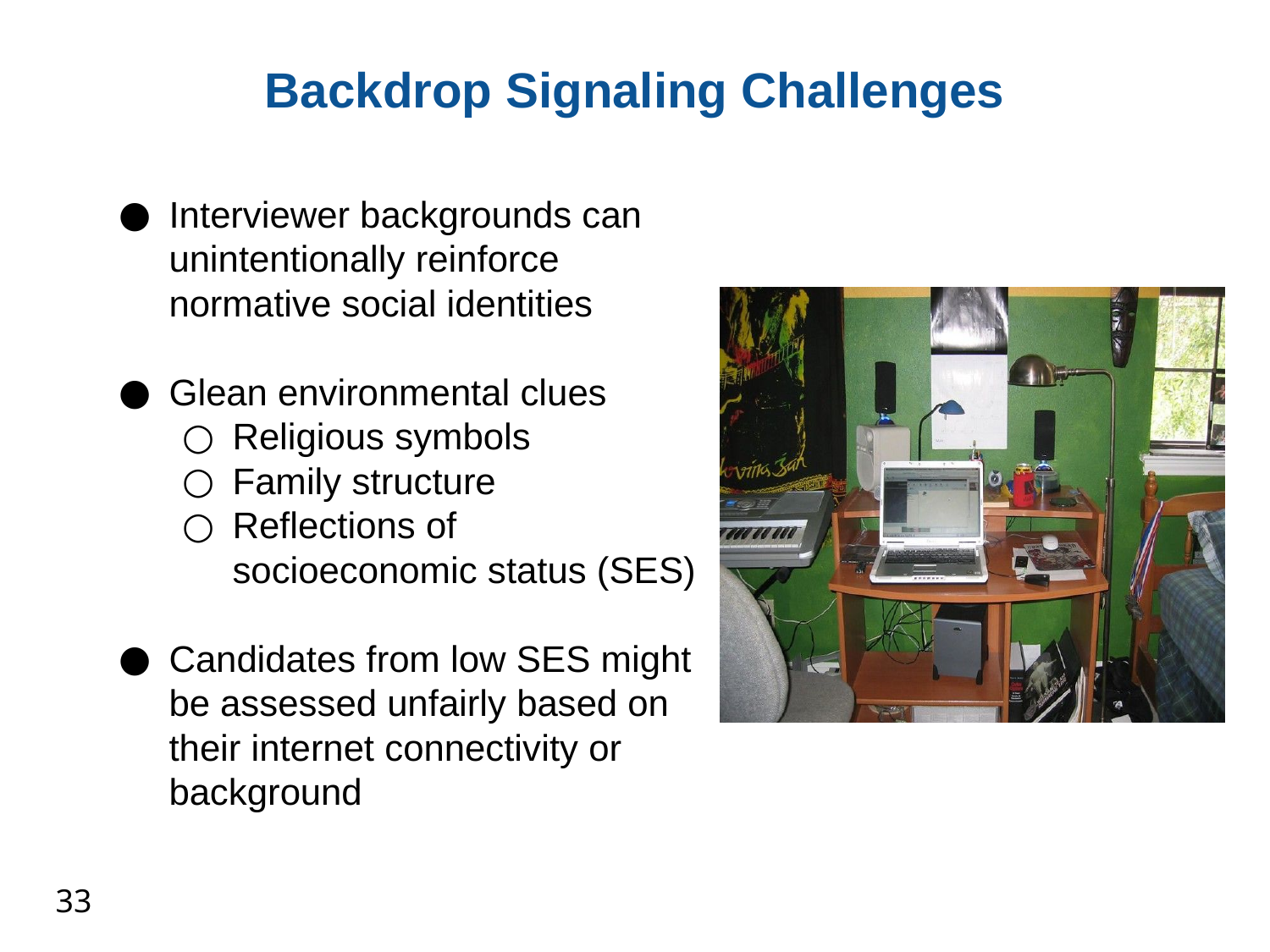

# Backdrop Signaling Challenges
Interviewer backgrounds can unintentionally reinforce normative social identities
Glean environmental clues
Religious symbols
Family structure
Reflections of socioeconomic status (SES)
Candidates from low SES might be assessed unfairly based on their internet connectivity or background
33

## Slide 34
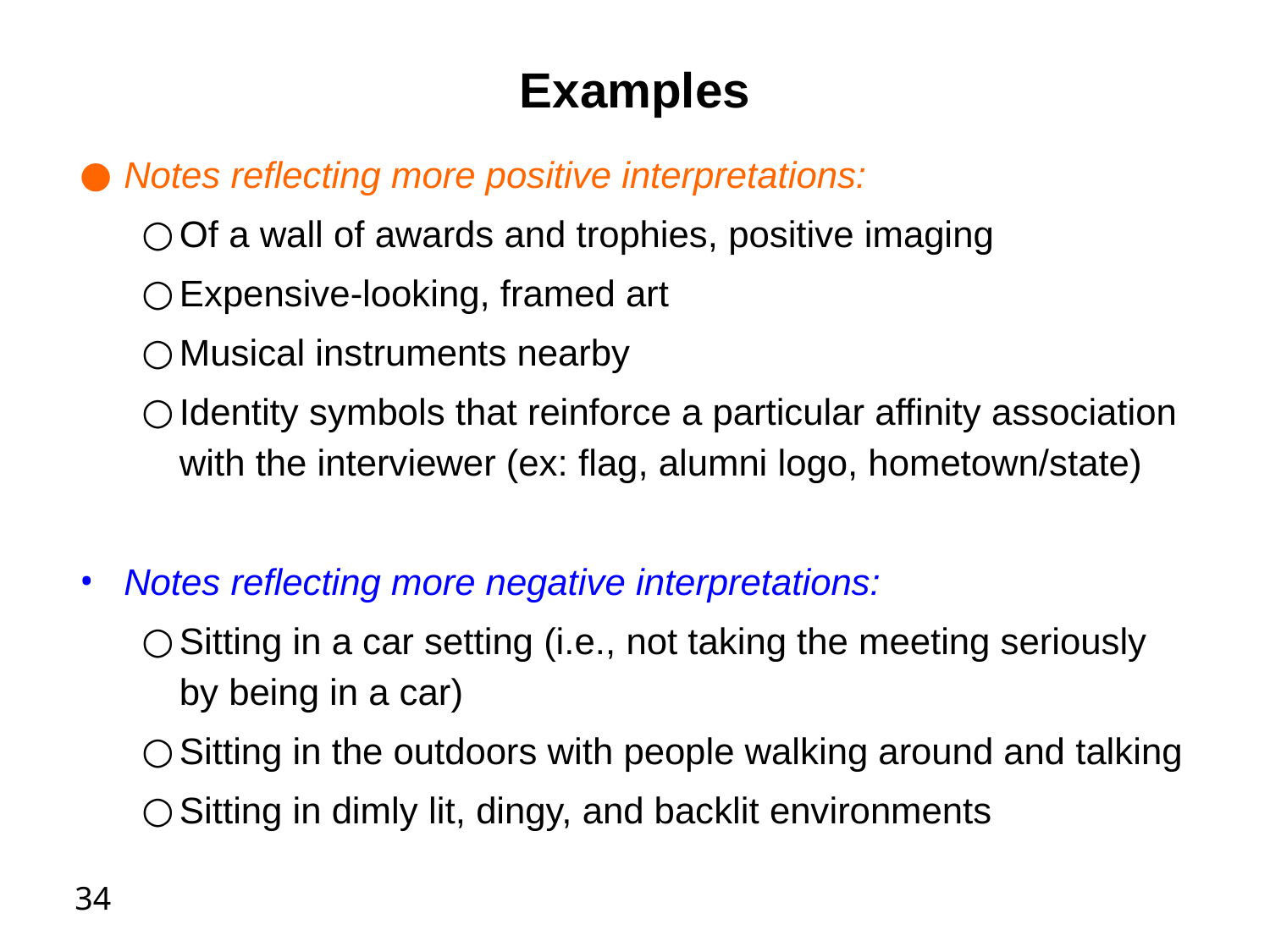

# Examples
Notes reflecting more positive interpretations:
Of a wall of awards and trophies, positive imaging
Expensive-looking, framed art
Musical instruments nearby
Identity symbols that reinforce a particular affinity association with the interviewer (ex: flag, alumni logo, hometown/state)
Notes reflecting more negative interpretations:
Sitting in a car setting (i.e., not taking the meeting seriously by being in a car)
Sitting in the outdoors with people walking around and talking
Sitting in dimly lit, dingy, and backlit environments
34

## Slide 35
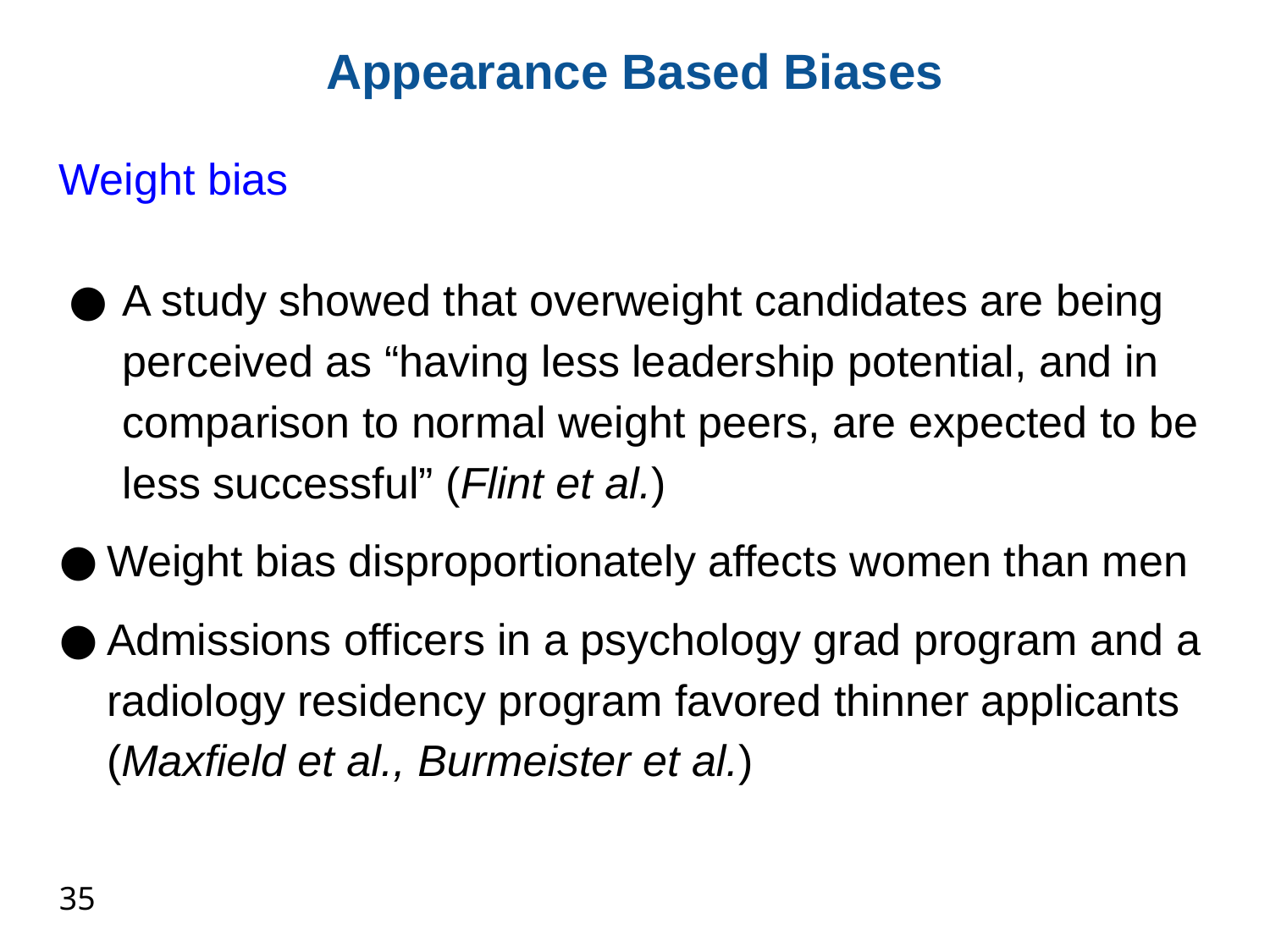

# Appearance Based Biases
Weight bias
A study showed that overweight candidates are being perceived as “having less leadership potential, and in comparison to normal weight peers, are expected to be less successful” (Flint et al.)
Weight bias disproportionately affects women than men
Admissions officers in a psychology grad program and a radiology residency program favored thinner applicants (Maxfield et al., Burmeister et al.)
35

## Slide 36
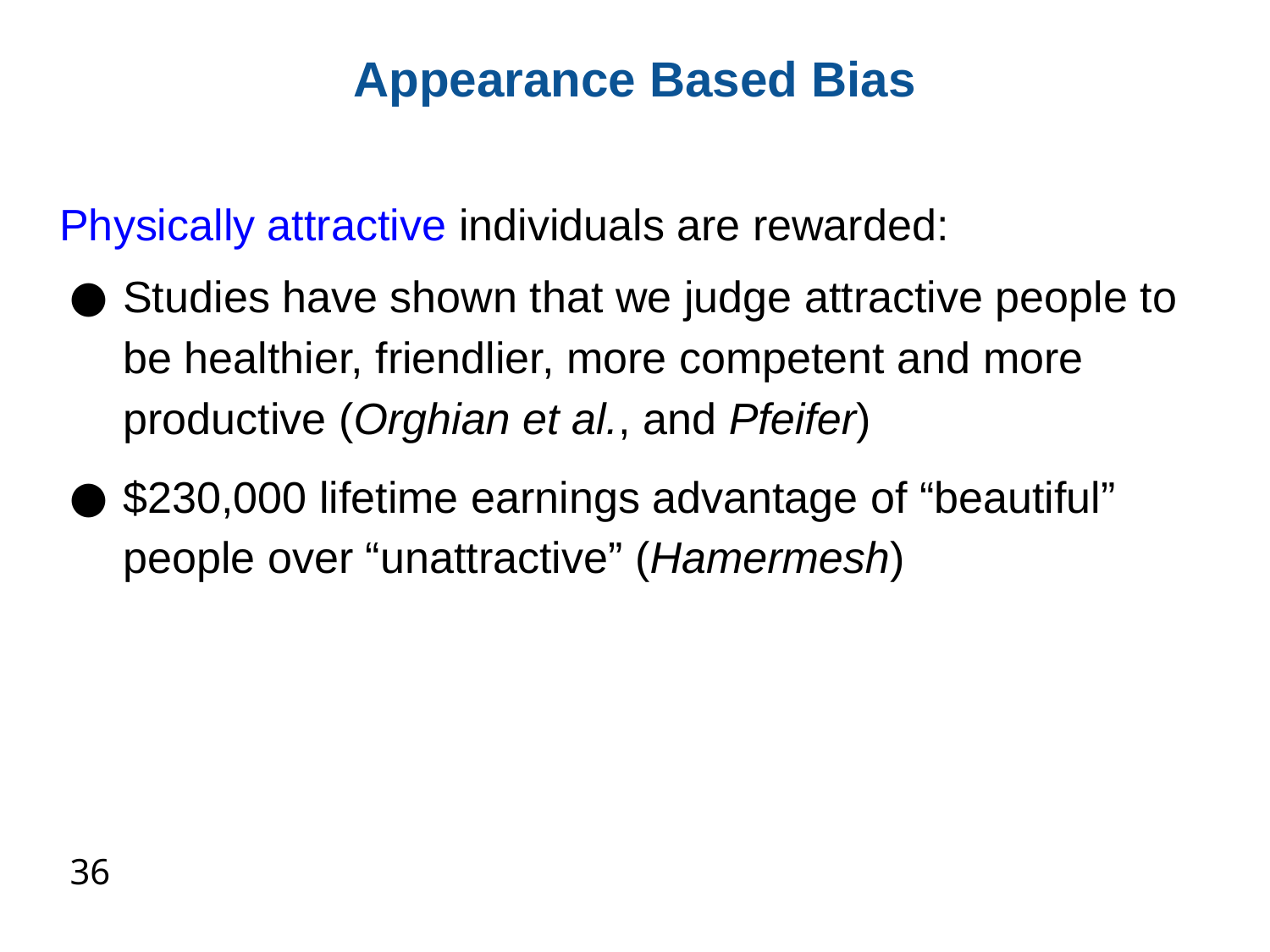

# Appearance Based Bias
Physically attractive individuals are rewarded:
Studies have shown that we judge attractive people to be healthier, friendlier, more competent and more productive (Orghian et al., and Pfeifer)
$230,000 lifetime earnings advantage of “beautiful” people over “unattractive” (Hamermesh)
36

## Slide 37
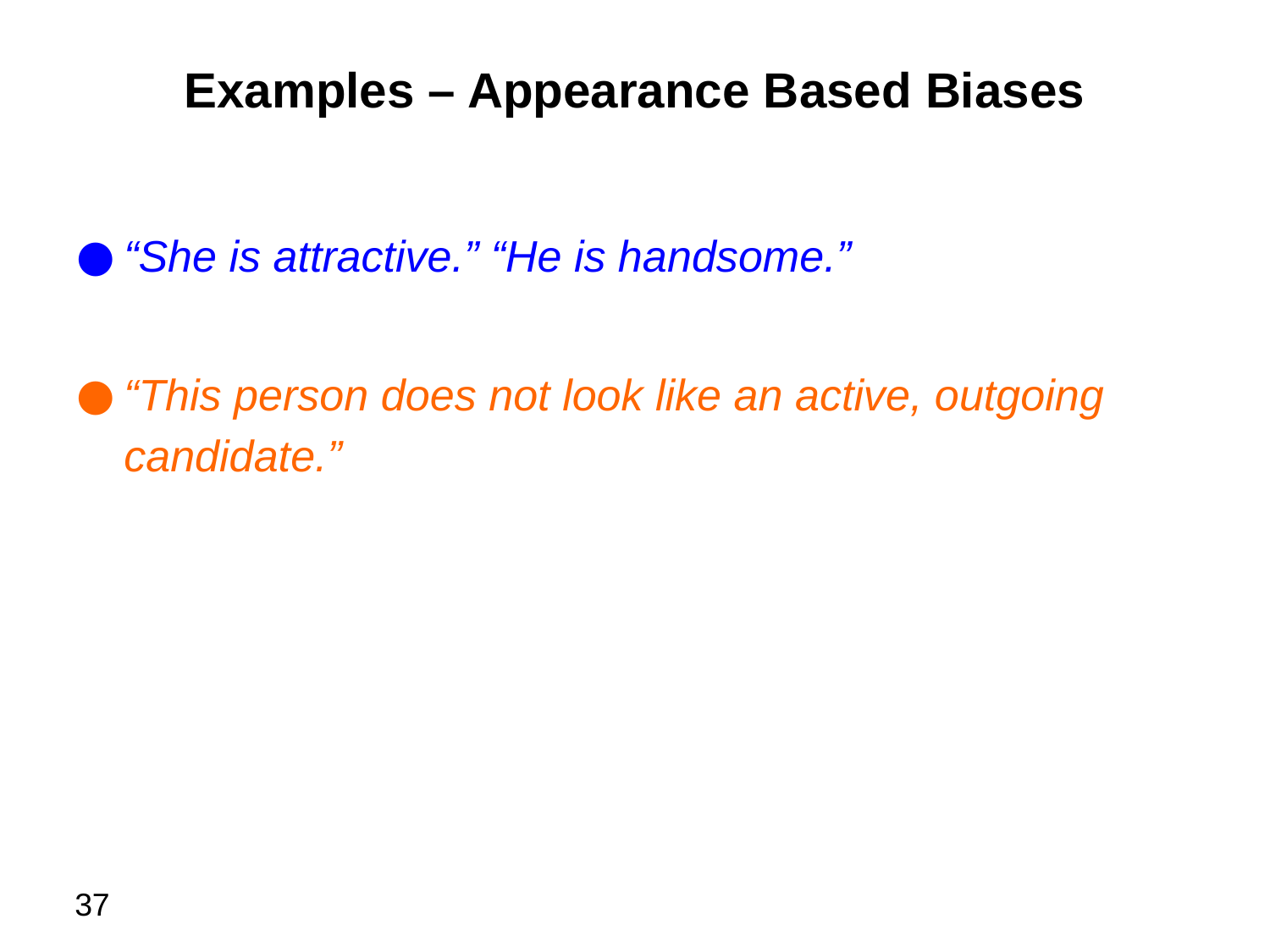

# Examples – Appearance Based Biases
“She is attractive.” “He is handsome.”
“This person does not look like an active, outgoing candidate.”
37

## Slide 38
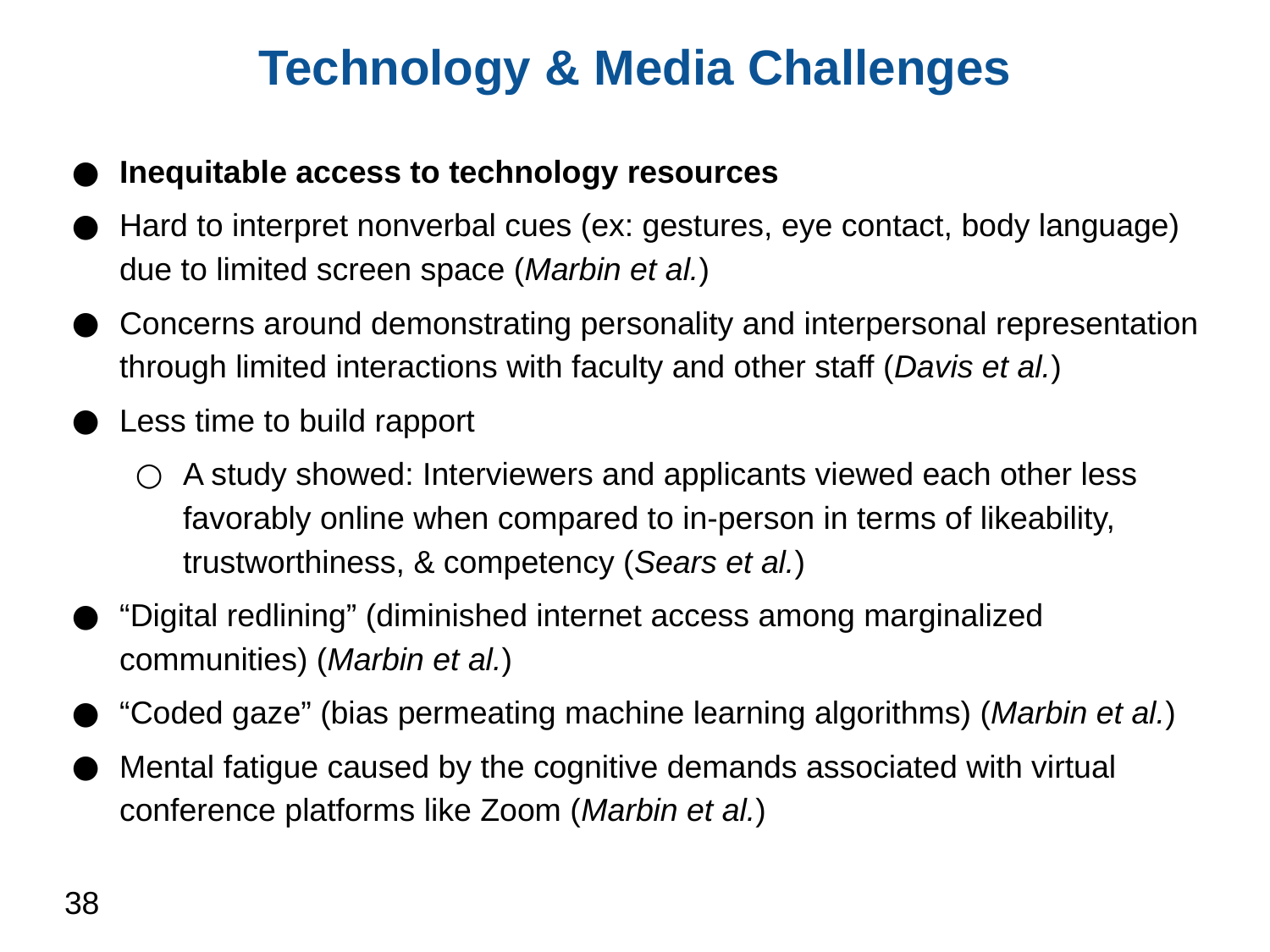

# Technology & Media Challenges
Inequitable access to technology resources
Hard to interpret nonverbal cues (ex: gestures, eye contact, body language) due to limited screen space (Marbin et al.)
Concerns around demonstrating personality and interpersonal representation through limited interactions with faculty and other staff (Davis et al.)
Less time to build rapport
A study showed: Interviewers and applicants viewed each other less favorably online when compared to in-person in terms of likeability, trustworthiness, & competency (Sears et al.)
“Digital redlining” (diminished internet access among marginalized communities) (Marbin et al.)
“Coded gaze” (bias permeating machine learning algorithms) (Marbin et al.)
Mental fatigue caused by the cognitive demands associated with virtual conference platforms like Zoom (Marbin et al.)
38

## Slide 39
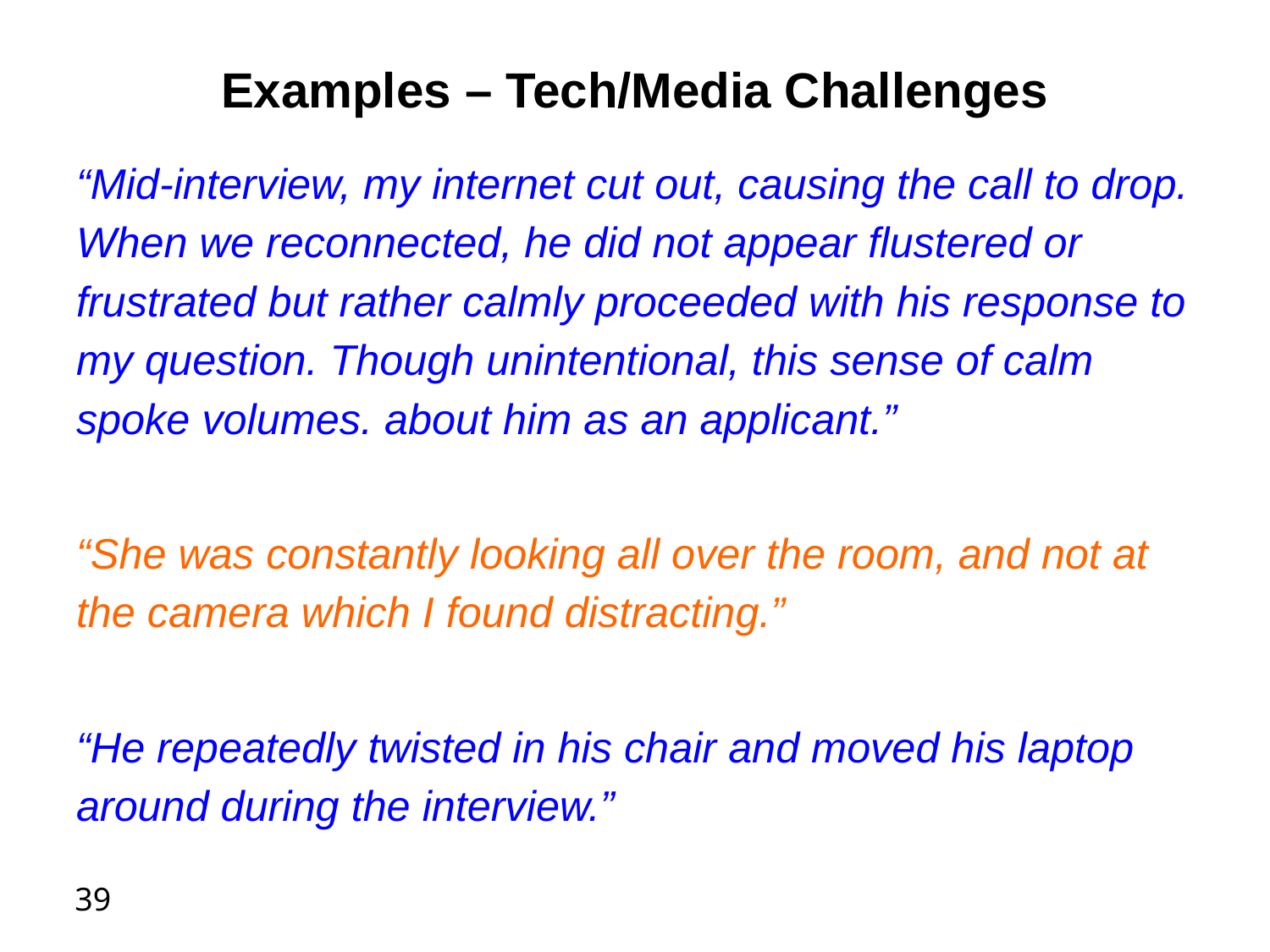

# Examples – Tech/Media Challenges
“Mid-interview, my internet cut out, causing the call to drop. When we reconnected, he did not appear flustered or frustrated but rather calmly proceeded with his response to my question. Though unintentional, this sense of calm spoke volumes. about him as an applicant.”
“She was constantly looking all over the room, and not at the camera which I found distracting.”
“He repeatedly twisted in his chair and moved his laptop around during the interview.”
39

## Slide 40
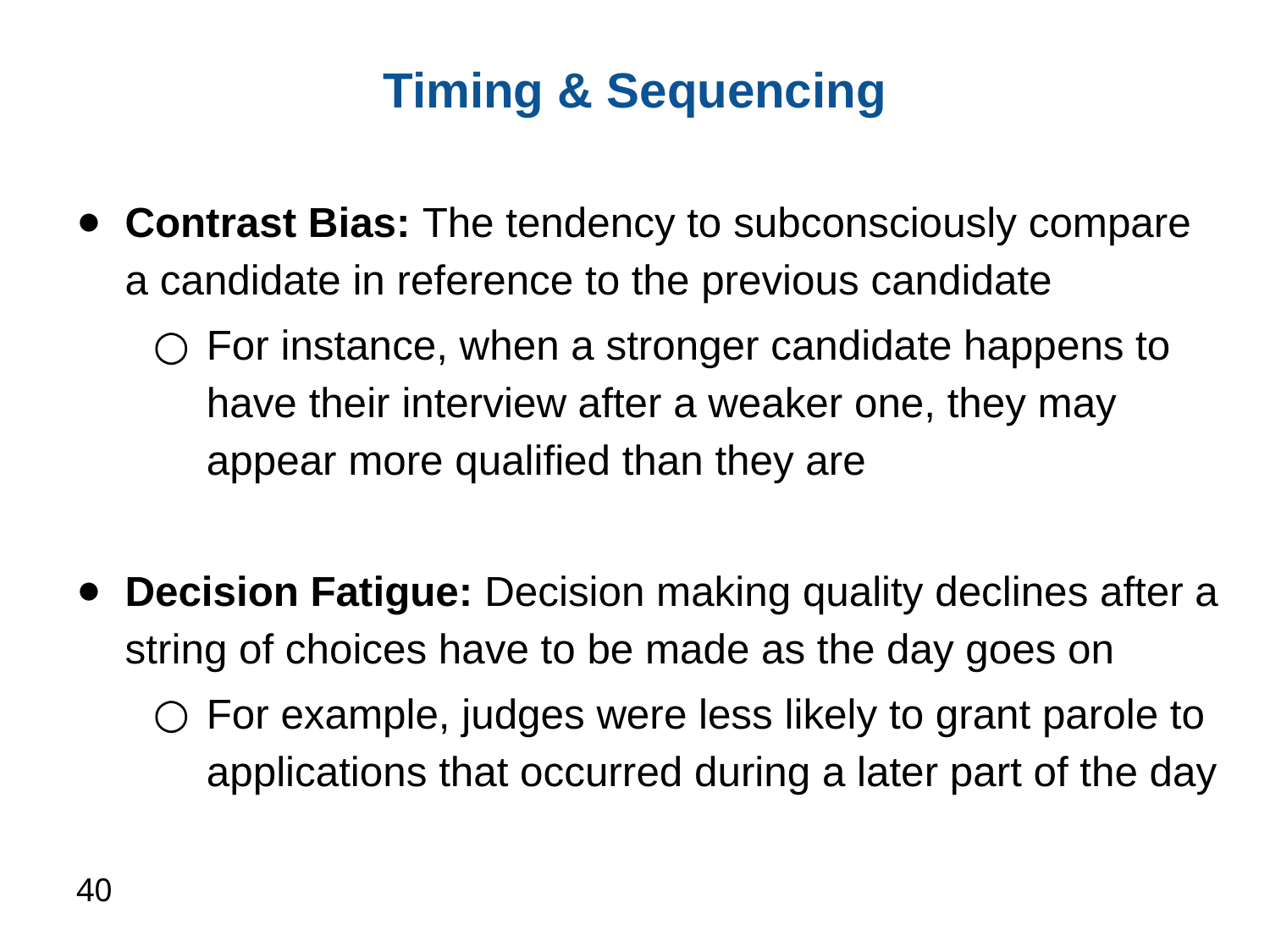

# Timing & Sequencing
Contrast Bias: The tendency to subconsciously compare a candidate in reference to the previous candidate
For instance, when a stronger candidate happens to have their interview after a weaker one, they may appear more qualified than they are
Decision Fatigue: Decision making quality declines after a string of choices have to be made as the day goes on
For example, judges were less likely to grant parole to applications that occurred during a later part of the day
40

## Slide 41
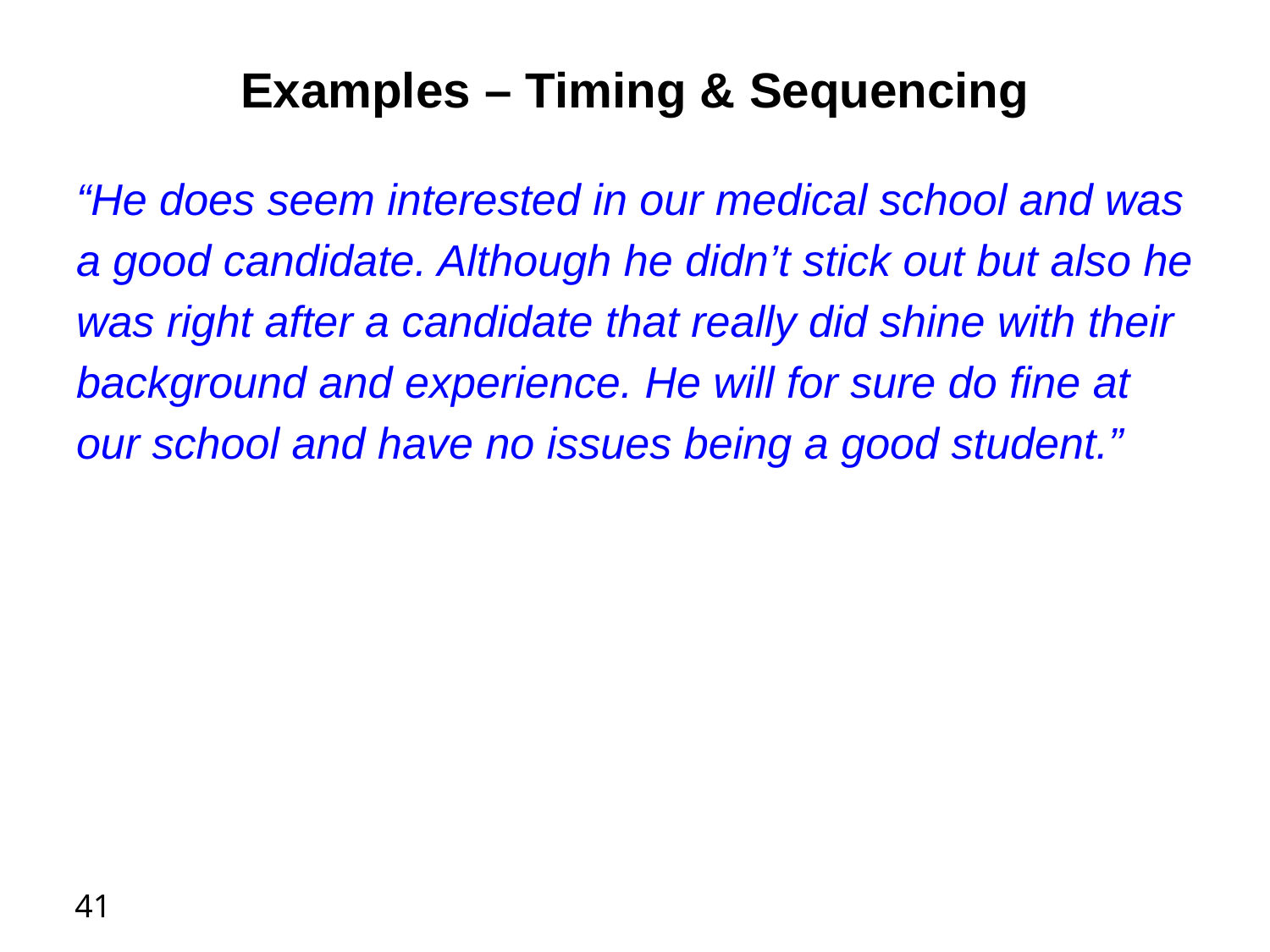

# Examples – Timing & Sequencing
“He does seem interested in our medical school and was a good candidate. Although he didn’t stick out but also he was right after a candidate that really did shine with their background and experience. He will for sure do fine at our school and have no issues being a good student.”
41

## Slide 42
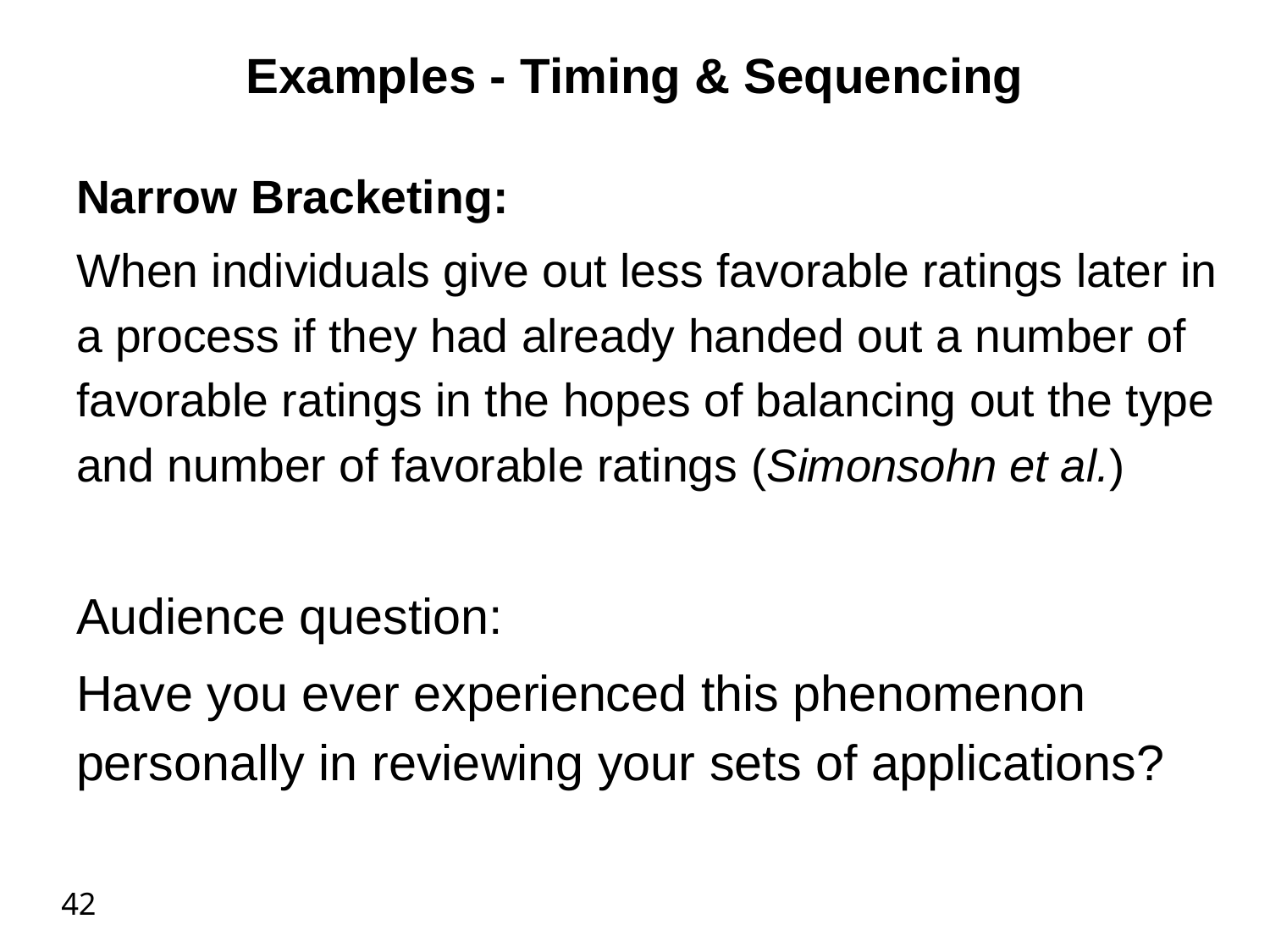

# Examples - Timing & Sequencing
Narrow Bracketing:
When individuals give out less favorable ratings later in a process if they had already handed out a number of favorable ratings in the hopes of balancing out the type and number of favorable ratings (Simonsohn et al.)
Audience question:
Have you ever experienced this phenomenon personally in reviewing your sets of applications?
42

## Slide 43
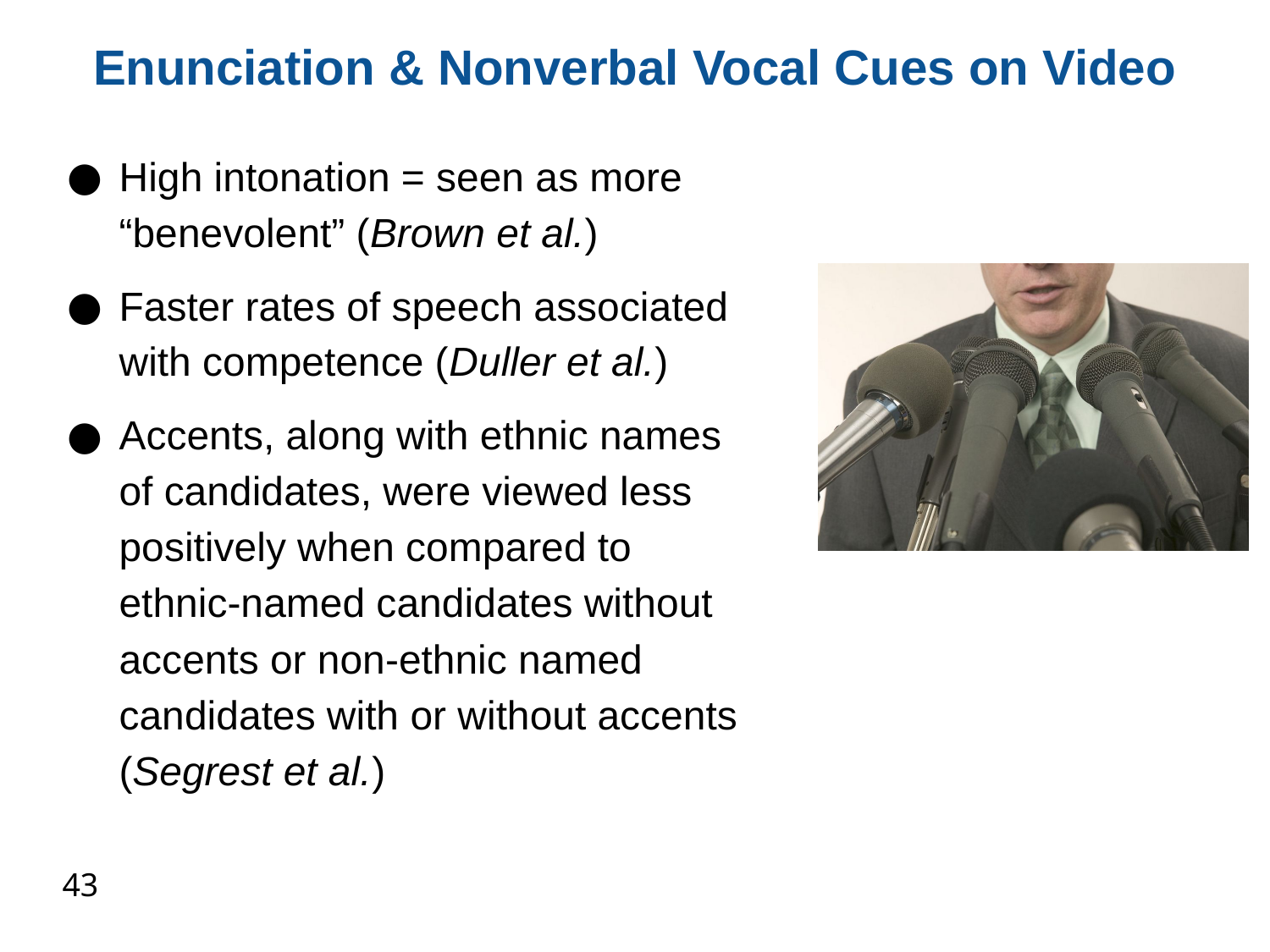

# Enunciation & Nonverbal Vocal Cues on Video
High intonation = seen as more “benevolent” (Brown et al.)
Faster rates of speech associated with competence (Duller et al.)
Accents, along with ethnic names of candidates, were viewed less positively when compared to ethnic-named candidates without accents or non-ethnic named candidates with or without accents (Segrest et al.)
43

## Slide 44
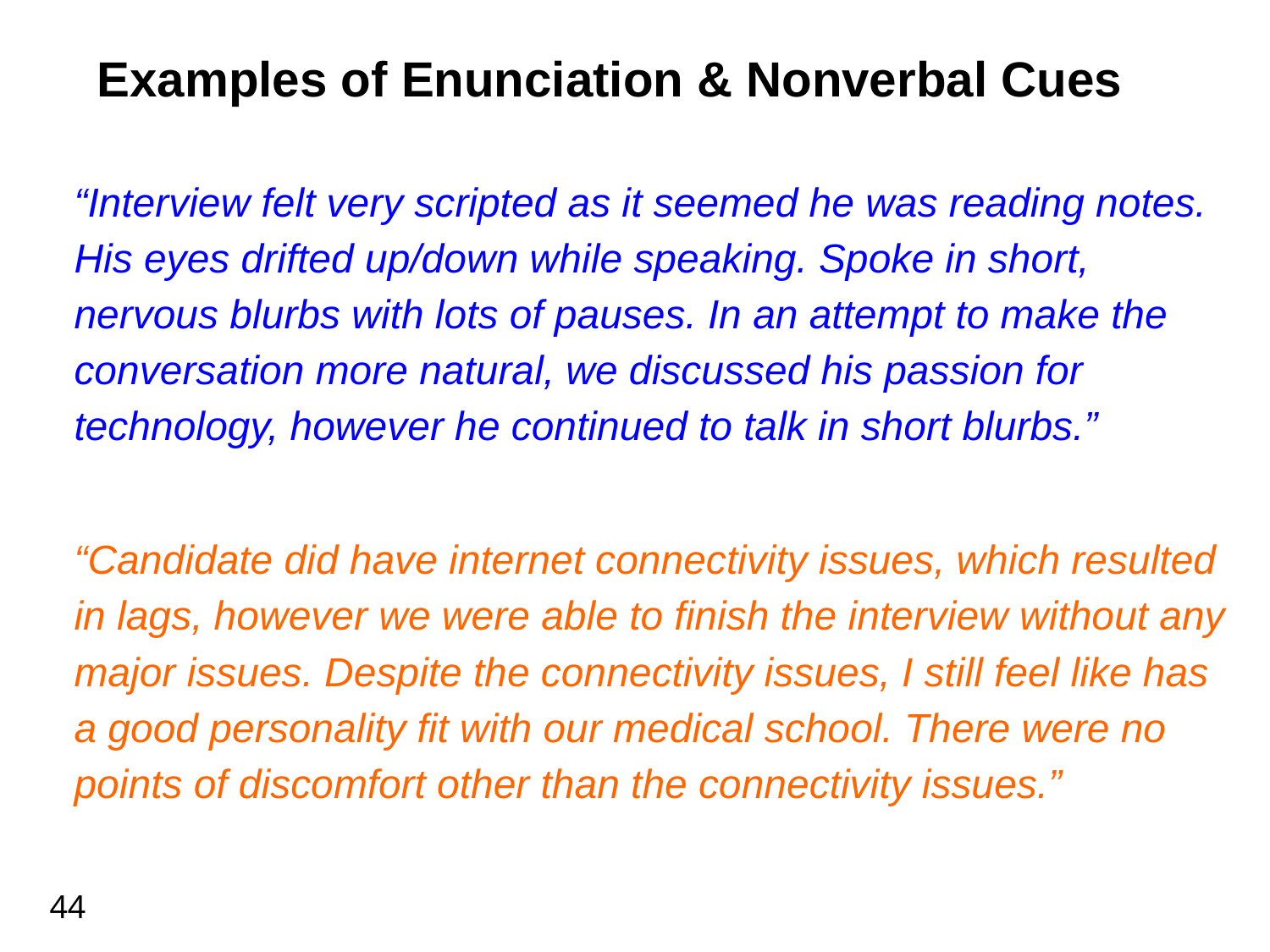

# Examples of Enunciation & Nonverbal Cues
“Interview felt very scripted as it seemed he was reading notes. His eyes drifted up/down while speaking. Spoke in short, nervous blurbs with lots of pauses. In an attempt to make the conversation more natural, we discussed his passion for technology, however he continued to talk in short blurbs.”
“Candidate did have internet connectivity issues, which resulted in lags, however we were able to finish the interview without any major issues. Despite the connectivity issues, I still feel like has a good personality fit with our medical school. There were no points of discomfort other than the connectivity issues.”
44

## Slide 45
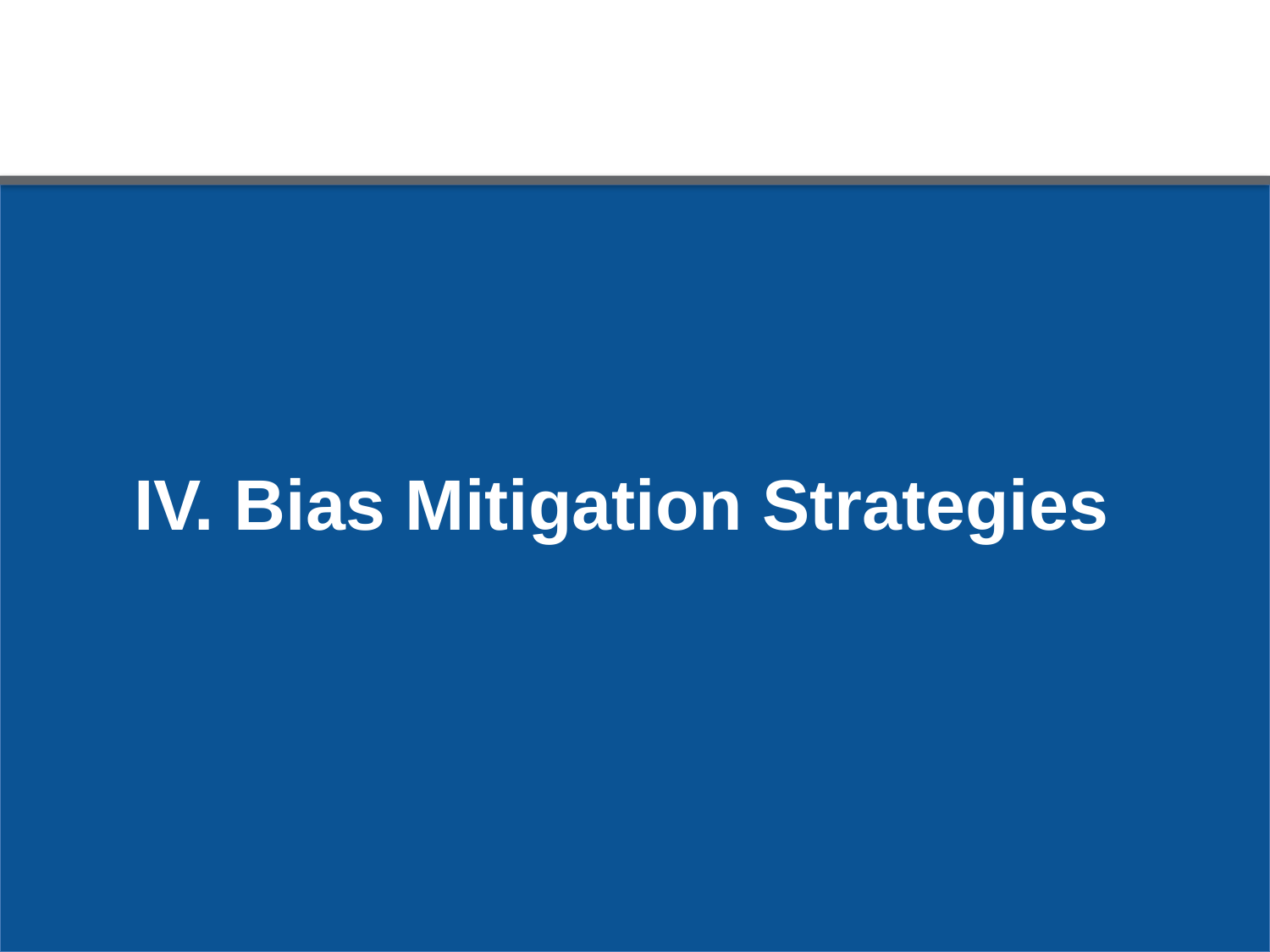

IV. Bias Mitigation Strategies

## Slide 46
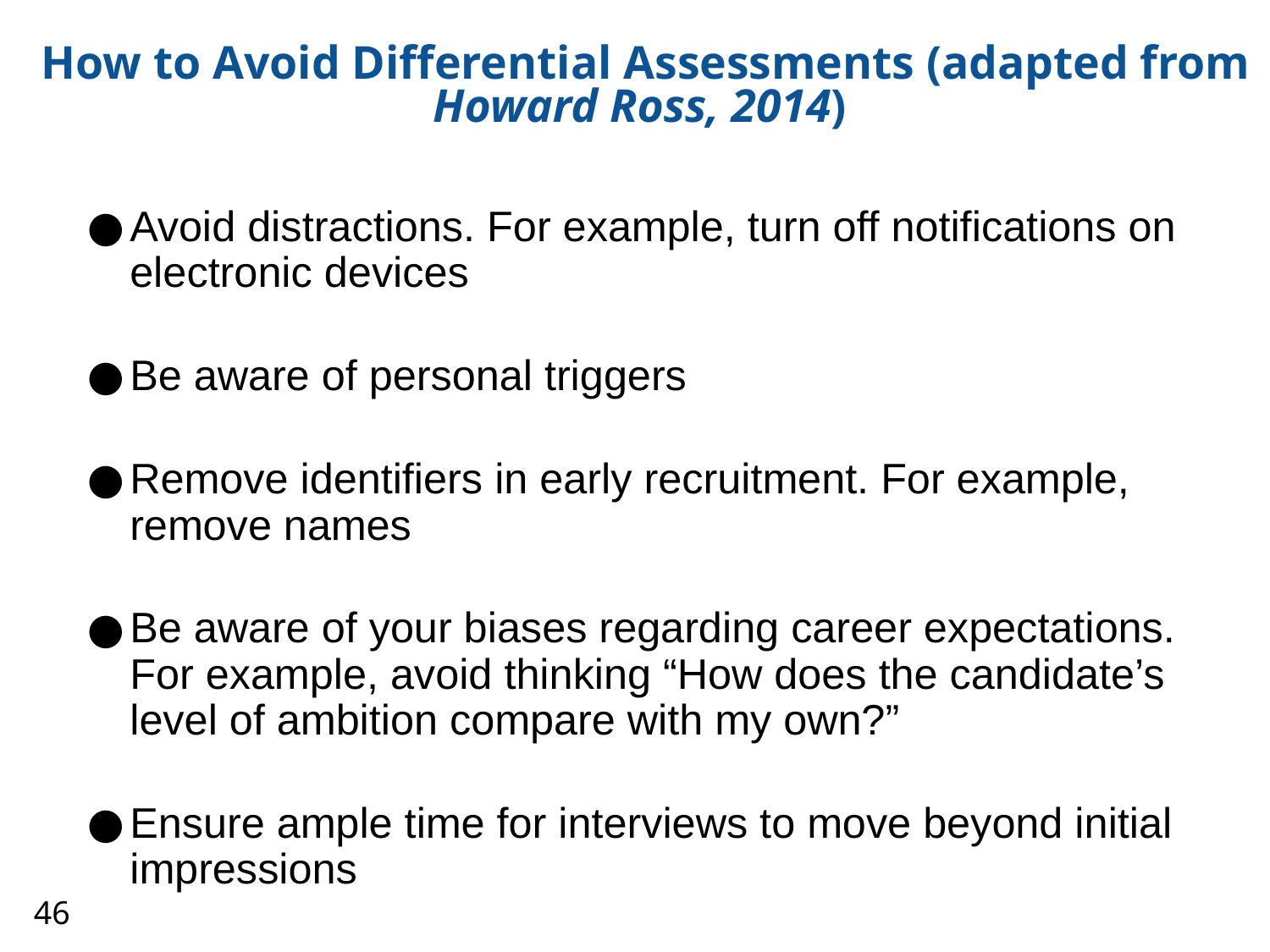

How to Avoid Differential Assessments (adapted from Howard Ross, 2014)
Avoid distractions. For example, turn off notifications on electronic devices
Be aware of personal triggers
Remove identifiers in early recruitment. For example, remove names
Be aware of your biases regarding career expectations. For example, avoid thinking “How does the candidate’s level of ambition compare with my own?”
Ensure ample time for interviews to move beyond initial impressions
46

## Slide 47
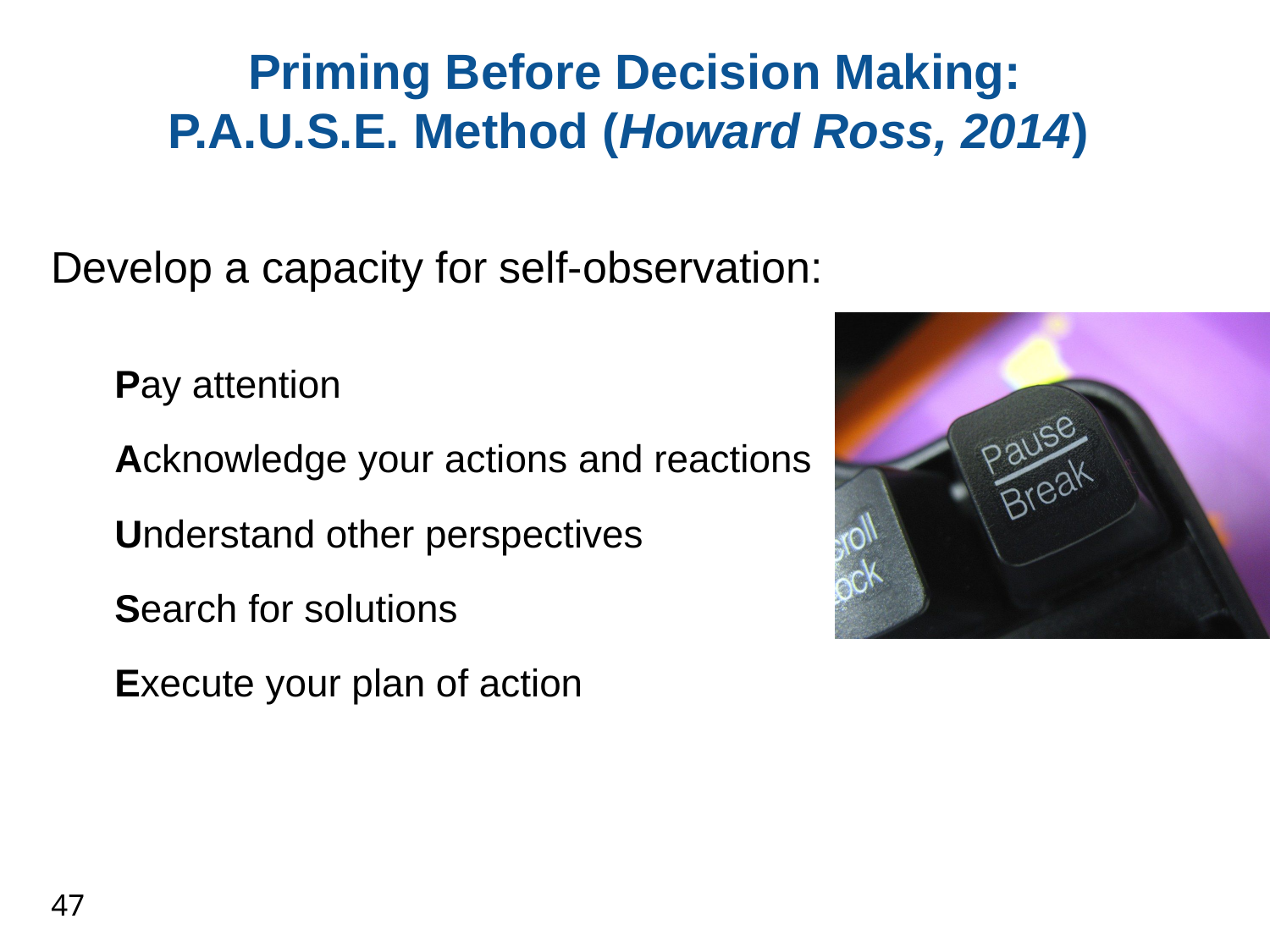

# Priming Before Decision Making: P.A.U.S.E. Method (Howard Ross, 2014)
Develop a capacity for self-observation:
Pay attention
Acknowledge your actions and reactions
Understand other perspectives
Search for solutions
Execute your plan of action
47

## Slide 48
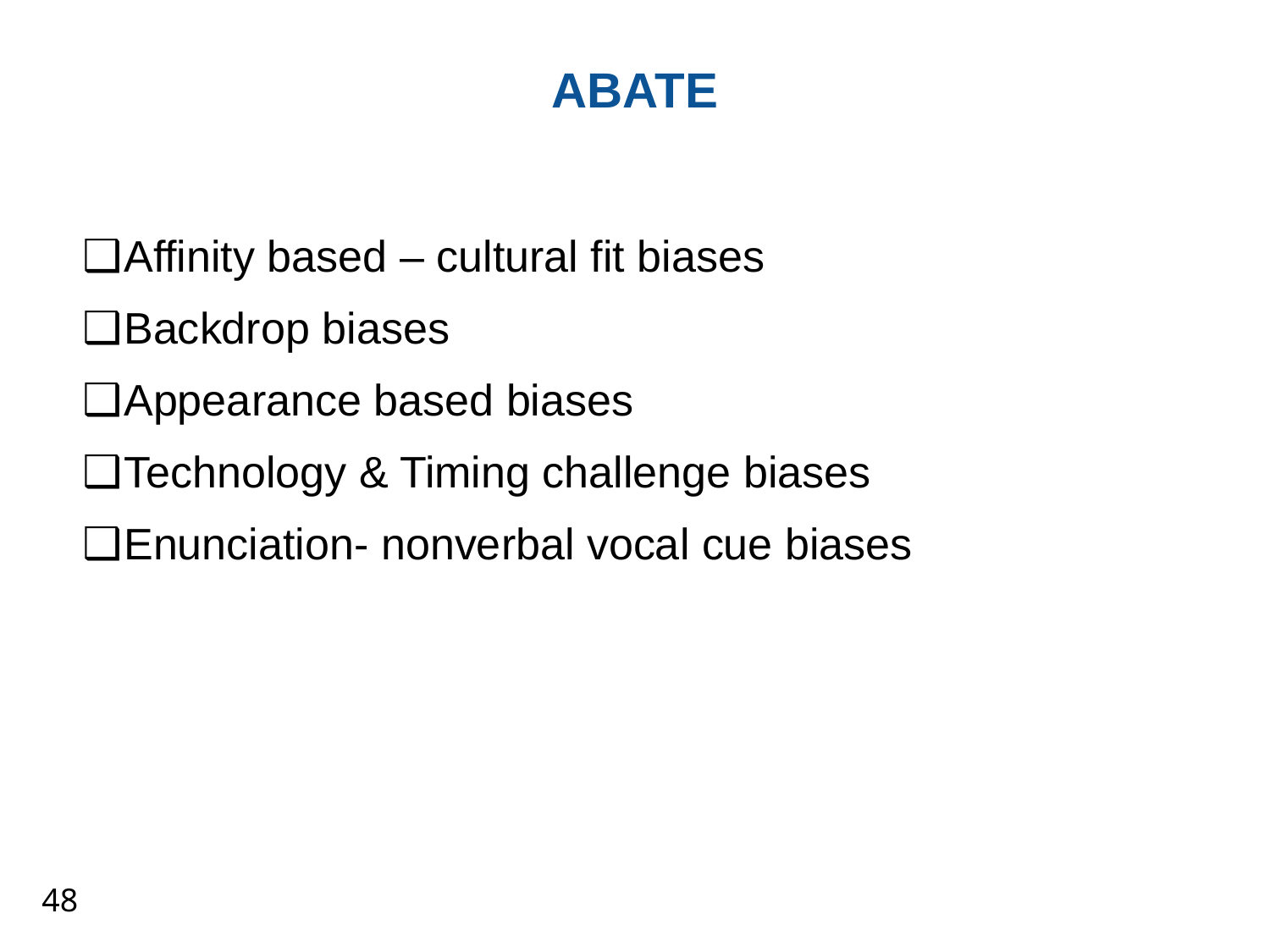

# ABATE
Affinity based – cultural fit biases
Backdrop biases
Appearance based biases
Technology & Timing challenge biases
Enunciation- nonverbal vocal cue biases
48

## Slide 49
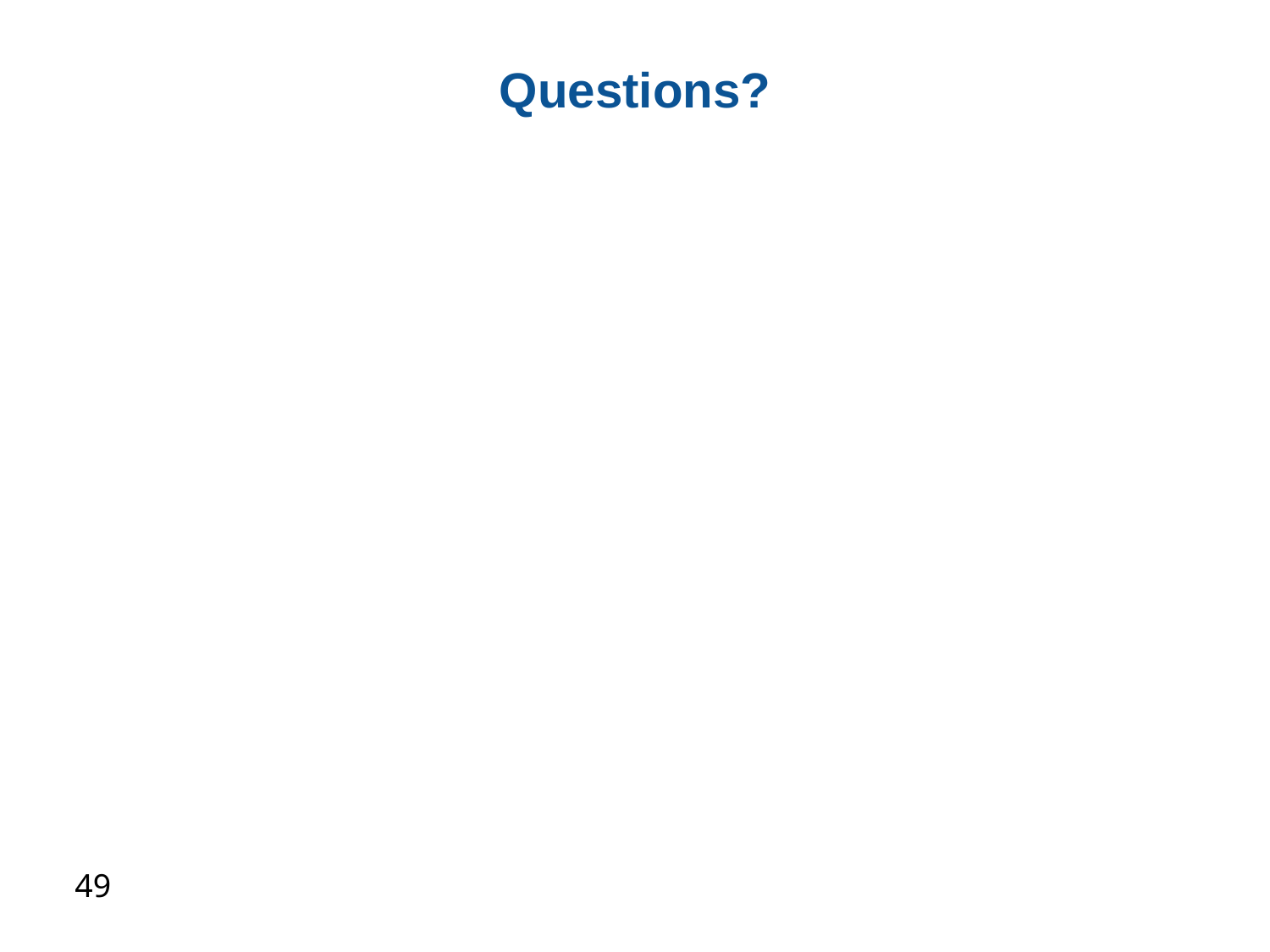

# Questions?
49
