# Supplementary material for: Evaluation and Assessment of the ABATE Framework to Enhance Implicit Bias Training for Virtual Interviews in Medical Schools
Source: MedEdPORTAL. 2024 Jun 28;20:11416. doi: 10.15766/mep_2374-8265.11416 (PMC11219124; doi:10.15766/mep_2374-8265.11416)
Supplement: Supplementary file 1 — ABATE Framework.docxLevels of Implementation.docxPreworkshop Evaluation Questionnaire.docxPostworkshop Evaluation Questionnaire.docxABATE Slide Deck.pptxABATE Speaker Notes.docx [file mep_2374-8265.11416-s001.zip › F. ABATE Speaker Notes.docx]

**ABATE PowerPoint Presentation Speaker Notes**

Slide 1: Title Slide & Introductions

Slide 2: Read a ‘No disclosures’ slide.

Slide 3: Announcement: This presentation is part of the ….

Present Appendix C

Slide 4: In this presentation, I will cover the following 3 learning objectives. Read off the list of objectives from the slide.

Slide 5: The Importance of Diversity. Share the importance of diversity to medical schools’ missions using information from AAMC. Note: Speaker can either read the slide’s text aloud or call on someone in the audience to read the text.

Slide 6: What is unconscious bias? Speaker reads off the definition of biases. Most of the time, bias can operate at the level of the unconscious. Everyone possesses biases. No one is immune from bias - including the presenter!

Slide 7: Cognitive Biases. Most of the time our mind works unconsciously and uses shortcuts (i.e., cognitive bias) to make inferences, which may influence our judgements, perceptions, and behaviors.

Slide 8: Activation Triggers

Bias is more likely to come into play and impact certain situations:

- Stress, exhaustion, and heavy workload.
- Essentially, in cases when we are mentally overloaded.
- Basically, biases come out when we are at our least best.

Slide 9: The Limits of Fairness & Objectivity. We might be overly confident in our assessment of being objective towards others. We might think, “I am fair and accurate in judging others.” “I am objective.” But there are what researchers Banaji and Greenwald, in their book “Blindspot,” have called “Mindbugs.” “Mindbugs” are “ingrained habits of thought that lead to errors in how we perceive, remember, reason, and make decisions” (Banaji and Greenwald, 2013).

Slide 10: Describe the Implicit Association Test with Harvard University Project Implicit. Website: <https://implicit.harvard.edu/implicit/iatdetails.html>.

“The Implicit Association Test (IAT) measures attitudes and beliefs that people may be unwilling or unable to report. The IAT may be especially interesting if it shows that you have an implicit attitude that you did not know about.” (https://implicit.harvard.edu/implicit/education.html)

Here, the speaker is encouraged to share associations they have or the tests they have taken. Describe the multiple tests that the audience can take online.

“The IAT measures the strength of associations between concepts and evaluations or stereotypes….Making a response is easier when closely related items share the same response key. (quote from FAQ section of IAT website, https://implicit.harvard.edu/implicit/user/demo.canada/ca.static/faqs.html#:~:text=The%20Implicit%20Association%20Test%20(IAT,share%20the%20same%20response%20key.)”

The speaker is also encouraged to emphasize that the IAT results are not prescriptive, but a beginning point for introspection and establishing mitigation strategies.

Slide 11: Divider for Section 2 - “Common Interview Biases”

Slide 12: COVID 19 Contexts and changes in AAMC Holistic review considerations. Of the list of reasons for changing contexts, the speaker can spotlight a few of the bullets included on the list.

Slide 13 and Slide 14: Two Most Overrated things in interviewing/selections.

One is when people say “Culture Fit” to approximate their sense of how well the candidate is perceived. This term is often overgeneralized and not backed by specifics and can easily allow for unconscious bias to influence how a candidate is perceived and assessed. A useful example from Forbes can be mentioned where if a candidate shares a favorite sports team with their interviewer, this similarity between the candidate and interviewer can be misconstrued as similarity between the candidate and the institution. (<https://www.forbes.com/sites/paulspiegelman/2021/03/01/is-hiring-for-culture-fit-perpetuating-bias/?sh=65dbadfa55e8>)

Another term that’s overused in interview assessment is when one says “Gut Feeling” to signify their overall impression of a candidate. Again, this term is often used to overgeneralize without backing up by specifics - a kind of “catch-all” term that is used to lean into biases without looking at specific merits and examples of candidates, leading to decreased diversity. A helpful idea paraphrased from LinkedIn can be brought up - intuition does not equal expertise since our subconscious uses overgeneralized cues to create unconscious ideas and patterns. (<https://www.linkedin.com/pulse/gut-feel-unconscious-bias-when-should-we-trust-our-intuition-whelan/>)

Be cautious of these two overrated summary statements.

Slide 15: Some food for thought for the audience. Another way to think about bias is through a question. “Who do you give the benefit of the doubt to and why?” Flipped, the question can also be asked, “Who don’t you give the benefit to and why not?”

Slide 16: Cover factors that can activate bias for or against an applicant featured on the chart on this slide. Start with negative factors and then proceed to positive. Ask the participants if they have examples of any of the factors on the chart from previous selection processes or interviews that they have been a part of. Source for the chart: https://www.washington.edu/diversity/faculty-advancement/handbook/assessment/

Share Appendix A and Appendix B on your computer and briefly review the content with the attendees. Presenters can read off the documents directly or encourage participation by asking volunteers to read.

Slide 17: Common Biases featured here. This is not a complete, exhaustive list of all biases that can impact interviews - but a curated list. Speaker goes through and describes each and then states that we will cover a few examples and have them identify which bias type they think it is. A good reference list that the speaker could share with folks if they want to look up these biases is: Indeed’s Types of Interviewing Bias and How to Minimize Them (Feb 3, 2023) <https://www.indeed.com/career-advice/interviewing/interviewing-bias>.

Slide 18: Name that Bias Exercise! Read the example text below or ask an audience member to read it aloud. Then pose to the audience a question asking what bias they think it is.

*“She came off somewhat cold and aloof; very stoic. Almost robot-like. Didn’t seem very warm and friendly. It is clear that she is a very ambitious and determined individual who has worked hard to get to where she is. Her somewhat cold and stoic demeanor, however, were not super warm and inviting. No doubt she will be a very competent medical student and doctor, but I am not sure that her personality would fit in well with other applicants I have interviewed and recommended to be a part of the incoming class.”*

Typical answers can be:

Choice B (Positive/Negative Stereotypes): Example to discuss with the audience: Gendered expectations about demeanor seem to be activated here. Are male candidates also equally expected to be “warm” and “inviting”?

or Choice D (Likeability bias) Example to discuss with the audience: Women are expected to be warm and likable to be seen as competent.

Once the audience members contribute examples of what they are seeing and specifics in the example text, conclude by acknowledging what they see and that there can be multiple biases at play in the text at hand.

Slide 19: Name that Bias Exercise! Read the example text below or ask an audience member to read it aloud. Then pose to the audience a question asking what bias they think it is.

*“If given the choice between our university and a state school, my gut tells me he would choose his state school. Overall, tough to assess--I would say this was the weaker of the three interviews I had on this day in terms of "interest." At times, the interview felt less conversational (compared to the other two applicants I had interviewed that day), but he was able to speak clearly and eloquently about his experiences especially towards the end of the interview.”*

Typical answers can be:

Choice C (Contrast Bias) Example to discuss with the audience: The phenomena of giving more or less value to an object when comparing it to another object. You can share that most interviewers strive for holistic review for each candidate on their own merits and that this interviewer specifically juxtaposes one candidate against three others, which is not best practice.

or Choice E (Elitism Bias) Example to discuss with the audience: Attitudes and beliefs favoring socially elite and prestigious classifications. The comment about choosing his state school and him being weaker of the candidates could point to this type of bias.

Once the audience members contribute examples of what they are seeing and specifics in the example text, conclude by acknowledging what they see and that there can be multiple biases at play in the text.

Slide 20: Name that Bias Exercise! Read the example text below or ask an audience member to read it aloud. Then pose to the audience a question asking what bias they think it is.

*“I have concerns regarding the candidate’s commitment to medicine. With her expressed desire to have a family and inquiries around a balanced schedule to raise children... She is not realistically appraising the demands of the profession and might not be realistic in the commitment required upfront to succeed.”*

A typical answer can be:

Choice B. (Positive/Negative Stereotypes) Making judgments and taking action based on socialized patterns, narratives etc. Here, there is a gender stereotype bias about women raising children and contributing to the family.

Once the audience members contribute examples of what they are seeing and specifics in the example text, conclude by acknowledging what they see.

Slide 21: Facilitator, please read the bullet points on the slide for discussion. Observed common errors in interview notes. Being over general, writing minimal notes, not being specific enough, or deviating from using the standard rubric.

Slide 22: Facilitator, please read the bullet points on the slide for discussion. Describe any number of doubt raisers that can occur in letters of recommendation (refer to Trix et al. on Slide 25 for a more comprehensive discussion). Any negative, unexplained comments, or irrelevancies can cast doubts when assessing candidates. The doubt raisers can also layer on each other!

Slide 23: Spot the Doubt Raisers Example. Read the example text below or ask an audience member to read it aloud. Then pose to the audience a question asking what doubts they can see in the text.

*“She is a very good applicant and has very strong clinical involvements. She has a strong desire to attend and would likely be a leader in the track. My only concerns were in her communication style, and the way that I perceived her ability to handle a stressful situation. She seemed to be easily rattled, but to her credit would recover and answer questions very articulately when given the time and space to gather her thoughts. I am unsure how strong her support system is and coping mechanisms are but I don’t think that it is necessarily an issue but I feel that they are noteworthy. I think that she could be a good medical student.”*

Typical answers can be:

- “She seemed to be easily rattled”, or the phrase, “I am unsure…”
- You can also mention that there are multiple doubts listed in this interviewer response and that they can layer on top of each other and compound in terms of overall negative impact.

After audience members share what they think the doubt raisers are, move on to Slide 24 which highlights sample ones.

Slide 24: Shows the examples of the doubt raisers highlighted in red, with a focus on the phrases: “My only concerns” “seemed to be easily rattled” “I am unsure how strong her support system is”

Slide 25: Introduce the concept of “Letters of Minimal Assurance.” Letters of minimal assurance is a term introduced in the scholarly research of Trix, F. & Psenka, C. (2003). *Exploring the color of glass: Letters of recommendation for female and male medical faculty.* There, letters of minimal assurance are characterized by three defining traits:

- brevity
- lack specificity/examples of specific accomplishments
- lack of evaluation of candidate’s traits

Notably, the Trix & Penska paper also identified with statistical significance that “fully 15% of the letters for female applicants fit into this category of letters of minimal assurance, whereas only 6% of the letters for male applicants fit into this mold” (Trix et al.)

Slide 26: Example of Minimal Assurance concept applied to interview notes. Read both versions below for the audience members.

Version 1 is from an interviewer capturing candidate notes and is very sparse and lacking details, whereas Version 2 is more detailed and talks about traits of the candidate.

Sample Interview question: Why are you interested in medicine?

Recorded answer as written down by interviewer:

*Version 1 (minimal assurance): Candidate’s father is a doctor, and he has watched him practice in a small town. Mother is a nurse as well.*

*Versus a more detailed, specific write-up:*

*Version 2 (detailed)*

*Candidate notes that he had no "a-ha moment "and came upon the decision after his multiple experiences with the field. He notes that his mother is a nurse; and his first exposure to the field personally was through shadowing his orthopedic surgeons. Through his time in the mid-life organization and shadowing physicians, he notes that he really appreciated the way the physicians he saw interacted with their patients; and how they could act as advocates, not only for individual patients but for populations at large. He discussed his current research interests from sickle cell disease and COVID-19. He knew his research well, and he taught me something new regarding sickle cell disease patients and opioid use. His answers overall demonstrate a deep level of introspection and thoughtfulness - It was evident from my conversation that he has put a great deal of thought into not only his choice of career; but also into the things that he sees every day. He is able to think deeply and critically about the things he sees, and has a solution-oriented mindset as well.*

Point out to the audience that one is longer and contains more specific details highlighting the achievements, interests, and context of the candidate to make for a more concrete assessment versus using literally just 1-2 sentences to assess the candidate. In particular, for admissions committees that rank top candidates as a result of reading interviewer notes, the minimal assurance note, when compared to the fully fleshed out interview note, does not provide enough detail, context, and specificity to give the candidate the best set-up for review.

Slide 27: Divider for Section 3 - “ABATE”

Slide 28: ABATE is a mnemonic to remember common biases that can impact virtual interview settings. Speaker can read through each of the letters.

Slide 29: A= Affinity Bias. Define the bias - you unconsciously favor folks that remind you of yourself. A handy way of remembering this is this phrase: “Like Likes Like”

Slide 30: Example 1 of affinity bias. The speaker reads it or has someone else read it from the audience. Ask the audience to discuss what they see. Key phrase, “She has research lab experience with the surgeon at Hospital X that I know very well and highly consider.”

Slide 31: Example 2 of affinity bias. The speaker reads it or has someone else read it from the audience. Ask the audience to discuss what they see. Key phrase, “the family has a strong connection to our university, and he wants to be part of that connection.”

Slide 32: Example 3 of “opposite of affinity bias.” The speaker reads it or has someone else read it from the audience. Ask the audience to discuss what they see. Key phrase, “I don’t know whether this stems from a cultural difference or if he is the type of person who would open up over time…”

Slide 33: B= Backdrop Signaling: Interviewer backgrounds can unintentionally reinforce normative social identities (ex: “when the virtual background of a Zoom meeting attendee has pictures of his or her wedding, it unintentionally reinforces the idea that marriage is most fitting between opposite sexes.” (Amy Bonomi, MSU)) Also, one can glean environmental clues on a candidate's background/identity that can open up the interview to bias.

Slide 34: Speaker reads a few examples of positive and negative interpretations gleaned from different background settings. Open up to the audience for discussion. An initial discussion prompt could be: In your interview experiences, what background signals have you observed? What reactions did you have in each situational context and why?

Slide 35: A= Appearance based biases. Speaker defines appearance-based biases - weight focused.

Slide 36: A= Appearance based biases. Speaker defines appearance-based biases - beauty focused.

Slide 37: Speaker reads a few examples of appearance-based biases. Open up to the audience for discussion. An initial discussion prompt could be: In your interview experiences, when have you noticed the candidate’s appearance? What did you notice and how did you try to counter what you perceived as a result of what you observed? Or, did you have any reactions to the listed examples of interviewer comments on appearance?

Slide 38: T= Technology & Media Challenges. Speaker reads slide and makes a case for virtual contexts - challenges and opportunities. Speaker could highlight the challenges of expressing oneself - if you like to speak with your hands, the limited screen size of a laptop introduces challenges to reading nonverbal communication. Speaker should also address inequitable access to technology resources in this section.

Slide 39: Speaker reads a few examples of tech/media-based biases. Open up to the audience for discussion. A proven set of questions to stir up discussion: What are your thoughts on establishing eye contact virtually? What if the internet connection is poor - how do you account for that in establishing rapport or the lack thereof?

Slide 40: T= Timing/sequencing. Two types of biases described here - contrast bias and decision fatigue. Encourage the audience to share any personal examples.

Slide 41: Speaker reads a few examples of timing and sequencing. Open up to the audience for discussion. Initial discussion questions could include: When does contrast bias come into play? In your experience, how/when did you compare candidates to each other - how does this affect your evaluations of candidates - timing of day, when you review each of them? What do you find problematic about this example provided in the slides?

Slide 42: Speaker gives another example of timing and sequencing - the issue of narrow bracketing. Ask the audience if they have ever experienced this phenomenon in assessment?

Slide 43: E= Enunciation & Nonverbal Cues on Video. Speaker describes and reads through the list of challenges on the slide.

Slide 44: Speaker reads a few examples of timing and sequencing. Open up to the audience for discussion. Initial discussion questions could include: What do you think of these examples? Do they resonate with you? Do lapses in internet connectivity or pauses in speech impact the way you perceive how dynamic a candidate is and how poised/professional they are? Now that you know this information on bias tendencies, how will you mitigate the tendency to assess candidates differently?

Slide 45: Divider for Section 4 - “Bias Mitigation Strategies”

Slide 46: How to avoid differential assessments - read through the different strategies.

Slide 47: Priming and the PAUSE Method Mnemonic (attributed to Howard Ross)

Slide 48: Review of ABATE Mnemonic

Slide 49: Field questions, encourage reflections, and provide contact information for follow up.

Present Appendix D
