# Supplementary material for: Evaluation and Assessment of the ABATE Framework to Enhance Implicit Bias Training for Virtual Interviews in Medical Schools
Source: MedEdPORTAL. 2024 Jun 28;20:11416. doi: 10.15766/mep_2374-8265.11416 (PMC11219124; doi:10.15766/mep_2374-8265.11416)
Supplement: Supplementary file 1 — ABATE Framework.docxLevels of Implementation.docxPreworkshop Evaluation Questionnaire.docxPostworkshop Evaluation Questionnaire.docxABATE Slide Deck.pptxABATE Speaker Notes.docx [file mep_2374-8265.11416-s001.zip › A. ABATE Framework.docx]

This document is meant to be shared with the attendees during the first part of the presentation to provide a brief introduction to the ABATE model. Presenter can read off this document directly or encourage participation by asking volunteers to read. *(Activity duration < 5 minutes)*

**ABATE: Reduce Bias in Interviews**

| **ABATE Category** | **How It Presents Face-to-Face** | **Implications for Virtual Formats** |
| --- | --- | --- |
| **A**ffinity^1^ | Assessments of likeness & cultural fit can lead to biased perceptions of competency. | In virtual formats, the same considerations around “cultural fit” and likeness are made, with more attention to enhanced cues like shared similarities gleaned from symbols and images in virtual backdrops. |
| **B**ackground^2^ | This was not a typical challenge pre-pandemic during in person interviews where interview rooms were standardized. | With the move to virtual formats, interview backdrops reflect intimate details of personal life, and may ever so subtly influence perceptions of affinity and personal connection between the interviewer and candidate. |
| **A**ppearance-Based^3^ | Appearance-based biases are well documented and pervasive within in-person interviews. These include, but are not limited to, visual markers around race and ethnicity, perceived attractiveness, and weight. | In virtual interviews, attractive backgrounds, lighting, online filters, and the positioning of camera angles may sway our perception of appearances. |
| **T**echnology & Media^4,5^ | Technology and media challenges are not primary concerns in face-to-face interviews. In-person interviews are perceived to be fairer than other mediated interviews. Interviewers and candidates are able to more fully convey information to each other by assessing verbal and nonverbal cues during in-person interactions. | Technical challenges (ex: loss of internet signal, poor audio) can impact the quality of social interactions during the interview and influence overall perceptions of the candidate. These technical challenges may be disproportionately faced by individuals with low socioeconomic status. |
| **E**nunciation & Vocal Cues^6,7,8^ | Varied pitch and faster rate of speaking have been associated with impressions of competence and extraversion during assessments. Similarly, perceptions of accents (e.g., ethnic, regional, social class) may influence interview assessments. | The same in-person considerations apply to virtual interviews. However, in virtual interviews, candidates are interviewing from homes and other personal environments. Here, candidates may have less control over their shared environments and could be contending with background noise or technology issues that can interrupt their audio. |

Sources:

1. Rivera LA. Hiring as Cultural Matching: The Case of Elite Professional Service Firms. *American Sociological Review*. 2012;77(6):999-1022. https://doi.org/10.1177/0003122412463213.
2. Davis MG, Haas MR, Gottlieb M, et al. Zooming In Versus Flying Out: Virtual Residency Interviews in the Era of COVID-19. *AEM Education and Training*. 2020;4(4):443–446. doi:10.1002/aet2.10486.
3. Maxfield CM, Thorpe MP, Desser TS, et al. Bias in Radiology Resident Selection: Do We Discriminate Against the Obese and Unattractive?. *Acad Med*. 2019;94(11):1774-1780. doi:10.1097/ACM.0000000000002813.
4. Sears GJ, Zhang H, Wiesner WH, Hackett RD, Yuan Y. A comparative assessment of videoconference and face-to-face employment interviews. *Management Decision*. 2013;51(8):1733–1752. https://doi.org/10.1108/MD-09-2012-0642.
5. Marbin J, Hutchinson Y, Schaeffer S. Avoiding the Virtual Pitfall: Identifying and Mitigating Biases in Graduate Medical Education Videoconference Interviews. *Academic Medicine*. 2021;96(8):1120-1124. doi:10.1097/ACM.0000000000003914.
6. Brown LB, Strong WJ, Rencher AC. Perceptions of personality from speech: effects of manipulations of acoustical parameters. *The Journal of the Acoustical Society of America*. 1973;54(1):29–35. https://doi.org/10.1121/1.1913571.
7. Segrest Purkiss SL, Perrewé PL, Gillespie TL, et al. Implicit sources of bias in employment interview judgments and decisions. *Organizational Behavior and Human Decision Processes*. 2006;101(2):152-167. https://doi.org/10.1016/j.obhdp.2006.06.005.
8. Duller DB, LePoire BA, Aune RK, Eloy SV. Social Perceptions as Mediators of the Effect of Speech Rate. *Human Communication Research*. 1992;19(2):286-311. https://doi.org/10.1111/j.1468-2958.1992.tb00303.x.
