# Supplementary material for: Evaluation and Assessment of the ABATE Framework to Enhance Implicit Bias Training for Virtual Interviews in Medical Schools
Source: MedEdPORTAL. 2024 Jun 28;20:11416. doi: 10.15766/mep_2374-8265.11416 (PMC11219124; doi:10.15766/mep_2374-8265.11416)
Supplement: Supplementary file 1 — ABATE Framework.docxLevels of Implementation.docxPreworkshop Evaluation Questionnaire.docxPostworkshop Evaluation Questionnaire.docxABATE Slide Deck.pptxABATE Speaker Notes.docx [file mep_2374-8265.11416-s001.zip › B. Levels of Implementation.docx]

This document is meant to be shared with the attendees during the first part of the presentation to provide a brief introduction to applications of the ABATE model. Presenter can read off this document directly or encourage participation by asking volunteers to read. *(Activity duration < 5 minutes)*

**The ABATE framework can be used to design and implement effective interventions at three different levels**

| **Level of Intervention** | **Suggested Uses for the ABATE Framework to Mitigate Bias** |
| --- | --- |
| **Institutional** | Framework incorporated into training for committees responsible for selection: admissions, appointments & promotions, search committees, and residency program interviews. Framework made accessible to committees through multiple means: as slides in presentation decks, as an insert into an interviewer guide, as priming cards or as a checklist. |
| **Individual Interviewer** | As part of structured training, interviewers would receive summary information about the ABATE framework and would be encouraged to use an ABATE framework checklist to review in a just-in-time manner before interviewing. |
| **Interviewee/Candidate Preparation** | Framework distilled into a best-practices guidance document or slides within a webinar to neutralize the impact of implicit bias in the ways that interviewees can control: neutralizing their backgrounds, adjusting their video cameras and lighting to provide a clear image focused on their face, managing technology during the interview, and practicing enunciation. |
