# Supplementary material for: Evaluation and Assessment of the ABATE Framework to Enhance Implicit Bias Training for Virtual Interviews in Medical Schools
Source: MedEdPORTAL. 2024 Jun 28;20:11416. doi: 10.15766/mep_2374-8265.11416 (PMC11219124; doi:10.15766/mep_2374-8265.11416)
Supplement: Supplementary file 1 — ABATE Framework.docxLevels of Implementation.docxPreworkshop Evaluation Questionnaire.docxPostworkshop Evaluation Questionnaire.docxABATE Slide Deck.pptxABATE Speaker Notes.docx [file mep_2374-8265.11416-s001.zip › _Educational Summary Report_11416.pdf]

MedEdPORTAL® The AAMC Journal of Teaching and Learning Resources

Original Publication Open Access

Evaluation and Assessment of the ABATE Framework to Enhance Implicit Bias Training for Virtual Interviews in Medical Schools

Veda Nagubandi, Caleb C. McKinney, PhD, MPS, Nicole M. Houle, EdD, Kristi D. Graves, PhD, Susan M. Cheng, EdLD, MPP*

*Corresponding author: smc307@georgetown.edu

Abstract

Introduction: The influence of implicit biases in virtual interviews must be addressed to ensure equity within the admissions process. ABATE is a mnemonic framework of five specific categories of implicit bias (affinity-based, backdrop-based, appearance-based, technology and media-based, and enunciation-based biases) that should be anticipated and mitigated for faculty, staff, health professionals, and medical students who conduct virtual interviews at medical schools. **Methods:** A 60-minute workshop was developed to educate medical school admissions interviewers about the ABATE model and strategies to mitigate implicit bias during virtual interviews. Four workshops were held over 1 year totaling 217 individual attendees. The workshops were evaluated using a single-group, pre-post questionnaire designed with the Kirkpatrick evaluation model. **Results:** Attendees reported that they found the ABATE workshop useful and relevant to improving their ability to minimize implicit bias during virtual interviews. Significant improvements were found in attendee reactions to the utility of implicit bias training (M pre $= 2.6$, M post $= 3.1$, $p = .002$). Significant changes were also reported in attendees' attitudes about interviewing confidence (M pre $= 3.0$, M post $= 3.2$, $p = .04$), bias awareness (M pre $= 3.0$, M post $= 3.4$, $p = .002$), and identifying and applying bias mitigation solutions (M pre $= 2.5$, M post $= 3.0$, $p = .003$). Knowledge specific to backdrop-based biases also significantly increased (M pre $= 3.2$, M post $= 3.4$, $p = .04$). **Discussion:** The ABATE workshop demonstrates promise in mitigating implicit bias in virtual medical school interviews.

Keywords:
Bias Mitigation, Implicit Bias, Admissions/Selection, Assessment, Bias, Online/Distance Learning, Program Evaluation, Diversity, Equity, Inclusion

Educational Objectives

By the end of this activity, learners will be able to:

1. Identify common interview biases.
2. Apply the ABATE model to interview scenario examples, translating strategies from in-person interviews to virtual contexts.
3. Facilitate equitable and inclusive virtual interview processes, especially for candidates underrepresented in medicine.

Introduction

Implicit biases are the positive and negative attitudes or stereotypes that can unconsciously influence people's perception, actions, and decisions.[1] Consistent evidence demonstrates that implicit bias influences the recruitment, interviewing, assessment, and selection of applicants and candidates for positions.[2] Everyone is susceptible to implicit biases, which can be activated involuntarily, without individuals realizing their presence or influence on decisions.[3,4]

Interviewing is an interpersonal process in which subjective impressions can drive decisions for admissions and job selection.[5,6] Although all individuals hold implicit biases, everyone, including interviewers, can be susceptible to illusions of objectivity. When people believe they are objective, they may act on biases more frequently than individuals who acknowledge the subjective influence of initial impressions.[7,8] Medical education studies have demonstrated that interviewer bias affects how candidates' interviews are scored.[9] In a study examining medical school admissions interviews in Canada, interviewer mood impacted candidate ratings; candidates who interviewed on rainy days tended to receive a 1% lower score than others who interviewed on sunny days, controlling for the

Citation:
Nagubandi V, McKinney CC, Houle NM, Graves KD, Cheng SM. Evaluation and assessment of the ABATE framework to enhance implicit bias training for virtual interviews in medical schools. *MedEdPORTAL*. 2024;20:11416.
https://doi.org/10.15766/mep_2374-8265.11416

Copyright © 2024 Nagubandi et al. This is an open-access publication distributed under the terms of the Creative Commons Attribution license.

applicants' qualifications.[10] Contextual elements such as time pressure, cognitive load, and inattentiveness to tasks contribute to an environment where unconscious bias can be activated.[11] Fortunately, behavioral responses from implicit biases can be attenuated once interviewers gain awareness of their biases.[3,12]

In May 2020, the Association of American Medical Colleges (AAMC) advised medical schools to adopt either phone or online interviews, and the Coalition for Physician Accountability recommended that all interviews be conducted virtually for the upcoming application season.[13,14] The COVID-19 pandemic marked a "landmark change in the way [graduate medical education] selection will occur and may irrevocably change the process going forward."[15] Institutions embraced a sudden change to virtual interviewing formats given the decreased risks and costs for candidates and institutions.[16] A recent study indicated that although both medical students and residents prefer in-person interviews, both groups also point to the merits of virtual interviews as a practical option, given the benefits associated with reduced costs and time.[17]

In May 2023, the AAMC recommended that academic medical programs continue to employ virtual interview formats to improve equity and decrease environmental impact.[18] With virtual interviews remaining highly relevant, the influence of implicit biases during virtual interviews must be addressed to ensure equity in the interviewing process. These implicit biases can disproportionally and negatively affect applicants who are underrepresented in medicine.

Although the pervasiveness and adverse impact of implicit bias during in-person interviews are well documented, less is known about how bias manifests during virtual interviews and how best to mitigate its effects. To address this urgent need, author Susan M. Cheng developed ABATE, a practical framework to guide equitable virtual interview and selection processes.

ABATE is a mnemonic framework for five specific categories of implicit bias that should be anticipated and abated during interview processes, with attention to virtual settings:

- Affinity-based biases: in-group affinity based on likeness and cultural matching.[19]
- Backdrop-based biases: subtle cues based on background decor/screens found in the physical environment.[20]
- Appearance-based biases: attractiveness based on outward assessments of readily visible factors including weight, race and ethnicity, and perceived beauty.[21]
- Technology and media-based biases: technology challenges (i.e., limited access to internet, need for high-quality cameras and speakers, mental fatigue caused by the cognitive demands associated with virtual conference platforms like Zoom) can exacerbate biases and the potential for racial bias as certain aspects of video camera functioning (e.g., clarity of images, automatic adjustment of lighting) have been programmed based on individuals with lighter skin. Additionally, the move from 3D (in-person) to 2D (virtual) makes it more difficult to fully convey information, leading to interviewers and applicants viewing each other less favorably.[22-24]
- Enunciation or vocal cue-based biases: impression management can be swayed by vocal cues including tone, accent, pitch, and frequency.[25,26]

We developed a workshop to educate medical school admissions interviewers about the ABATE model and strategies to mitigate implicit bias during virtual interviews.

Methods

Four workshops were delivered between August 2021 (two workshops) and August 2022 (two workshops). We evaluated the effectiveness of the ABATE model using a single-group, pre-post test design, measuring attendees' levels of knowledge acquisition and changes in attitudes before and after attending the workshop.

We obtained approval from Georgetown University's Institutional Review Board to perform retrospective analysis of workshop evaluation materials (STUDY00004672).

Context of Prior Workshops for Admissions Interviewers

Annual workshops for admissions interviewers have been required and the content of these preparatory workshops has evolved over the years. In prior years (before 2016), the workshop outlined the admissions process and described admissions requirements and guidelines for competitiveness set forth by the selection committee. It also covered interview etiquette and provided requirements to interviewers about what questions to ask the applicants and what questions to avoid. Finally, the workshop delivered instructions on writing a comprehensive evaluation of an applicant's noncognitive skills (e.g., soft skills related to motivation, integrity, and interpersonal interactions).[27] Starting in 2016, the Office of Admissions expanded the interviewer workshop by including implicit bias training. When the COVID-19 pandemic forced the interview process to go virtual, the implicit bias training was adapted to the 60-minute ABATE workshop to instruct interviewers on ways to identify and mitigate implicit biases during virtual interviews.

Copyright © 2024 Nagubandi et al. This is an open-access publication distributed under the terms of the Creative Commons Attribution license.

Preparation

ABATE development was guided, in part, by Kern's six-step approach[28]:

- Step 1. Problem Identification and General Needs Assessment: consulted with admissions personnel and leadership during the start of the COVID-19 pandemic to assess the need for guidance on mitigating potential biases arising from the quick pivot to online interviews.
- Step 2. Targeted Needs Assessment: conducted a literature review to assemble evidence-based bias mitigation strategies into ABATE framework components.
- Step 3. Goals and Objectives: developed the following goals in conjunction with admissions personnel and staff at the Office of Diversity, Equity, Inclusion, & Belonging to update the already-required anti-racism training for the virtual interview context:
  - Evaluate the feasibility of incorporating the ABATE framework in interviewer training,
  - Measure current interviewer knowledge of issues surrounding implicit bias, and
  - Evaluate an ABATE workshop on interviewer reactions to the training, attitudes about implicit bias, and knowledge.
- Steps 4 and 5. Educational Strategies and Implementation: incorporated framework into an interactive and conversational style workshop that was made mandatory for all admissions interviewers by the Office of Admissions in August and September before the admissions cycle.
- Step 6. Evaluation and Feedback: collected feedback from pre- and postworkshop questionnaires and verbally from faculty and staff through follow-up assessments and conversations with the admissions team.[29]

Following development of the ABATE model and its supporting elements (the framework and related ABATE model materials: Appendices A and B), we developed pre- and postworkshop evaluation questionnaires (Appendices C and D).

Equipment

The workshops were delivered virtually through the Zoom audiovisual platform, and the ABATE workshop slide deck (Appendix E: slides, Appendix F: speaker notes) was shared through screen-sharing capabilities. All medical school admissions interviewers who attended the workshop needed a working computer or other device with an audiovisual system to participate and complete the pre- and postworkshop evaluation questionnaires.

Personnel

The 60-minute workshop included a designated question-and-answer period and was delivered by one facilitator with expertise in both bias mitigation and educational session delivery for faculty and staff using online platforms (e.g., Zoom).

ABATE Workshop: Procedures and Content

At the start of the workshop, attendees were given 5 minutes to complete an online anonymous preworkshop questionnaire. It should be noted that responding to the questionnaire was not required to continue to the next stage of the workshop.

In Part 1 (15 minutes) of the ABATE workshop, the facilitator described and defined types of bias, presented facts regarding obstacles to achieving diversity, and set the stage for the importance of mitigating implicit bias within virtual admissions interviews. This introduction also included examples of common interview biases in the context of virtual interviews (Appendix A) and examples of ABATE framework application at the three levels described as follows: (1) institutional (ABATE incorporated into training for selection committees responsible for admissions, appointments and promotions, and recruitment), (2) interviewer (ABATE incorporated as part of structured training and as part of a checklist to review in a just-in-time manner before interviewing), and (3) interviewee (ABATE distilled into a best-practices guidance document or webinar slides to prepare for interviews and neutralize the impact of implicit bias in ways that candidates can control; Appendix B). The facilitator encouraged participation by inviting attendees to identify types of biases evident in sample interviewer notes; attendees shared their ideas and thoughts by using either the chat or the audio functions in the Zoom platform.

In Part 2 (15 minutes) of the ABATE workshop, the facilitator discussed the structure of the ABATE model with example interview scenarios encapsulating each of the five types of implicit bias represented and encouraged participation by inviting attendees to identify types of biases involved in sample interviewer notes.

In Part 3 (15 minutes), the facilitator provided examples of bias mitigation from the ABATE model implementation materials.

Appendix materials were made accessible for attendees by uploading them to the resources tab on the software system (AMP by ZAP Solutions) that interviewers used to complete their assessments of interviewees. For interviewers who lacked access to AMP, files were also uploaded to the university's secure file-sharing server, Box, to ensure accessibility.

Copyright © 2024 Nagubandi et al. This is an open-access publication distributed under the terms of the Creative Commons Attribution license.

Debriefing

After parts 1-3 of the ABATE workshop, the facilitator encouraged interviewers to consider the material presented and share their reflections and questions for 5 minutes. The facilitator fostered an open discussion about attendee experiences while incorporating structured suggestions for individual improvement based on responses provided by the attendees to mitigate instances of implicit bias during virtual interviews. Following the debrief discussion, the facilitator directed attendees to take 5 minutes to complete an anonymous postworkshop questionnaire through a link provided in the Zoom chat. Similar to the preworkshop questionnaire, responses to the postworkshop questionnaire were not required.

Course Evaluation

Our evaluation followed the Kirkpatrick evaluation model, an empirically based approach used in academic curriculum development to evaluate training programs.[30] Level 1 of the Kirkpatrick evaluation model assesses attendee reactions regarding the utility of training programs, while level 2 assesses improvement in attendee expertise, knowledge, attitudes, and/or skill because of the training program.

Preworkshop questionnaire: The attendees completed a nine-item preworkshop questionnaire to assess baseline reactions to the utility of bias mitigation training programs, attitudes, and knowledge levels. All responses to the nine items were made using a 5-point Likert scale (*strongly agree, agree, neutral, disagree,* and *strongly disagree*). For level 1 of the Kirkpatrick evaluation model, one question assessed attendee reactions regarding the value of training programs (question 1 pertaining to Educational Objective 3; see Table 1). For level 2 of the Kirkpatrick evaluation model, three questions assessed self-reported attendee attitudes surrounding confidence in mitigating implicit bias and recognizing sources of implicit bias (questions 2-4 pertaining to Educational Objective 3; see Table 1), and five optional questions gauged baseline knowledge surrounding implicit bias recognition of the five tenets of the ABATE model (i.e., affinity-based, backdrop-based, appearance-based, technology and media-based, and enunciation or vocal cue-based biases; questions 5, 7, and 9 pertaining to Educational Objective 1; questions 6 and 8 pertaining to Educational Objective 2; see Table 1). It should be noted that other interpretations of the Kirkpatrick evaluation model could place attitude questions 2-4 under level 1 given that attitudinal responses about one's knowledge could be considered part of the reaction to training.

Postworkshop questionnaire: The postworkshop questionnaire consisted of the same nine questions as on the preworkshop questionnaire to assess attendees' reactions, attitudes, and knowledge. An open-ended question was also included for attendees to provide additional comments or suggestions for improvements in future ABATE workshops.

Results

The workshop was offered twice in 2021 (130 attendees; 59 completed the preworkshop questionnaire [45% response rate], and 26 completed the postworkshop questionnaire [20% response rate]) and twice again in 2022 (128 attendees; 16 completed the preworkshop questionnaire [12% response rate], and 15 completed the postworkshop questionnaire [12% response rate]). Across all workshops, we had a total of 258 attendees, of whom 41 attended the workshop twice (resulting in 217 unique attendees). For the 2021 admissions cycle, the Georgetown University School of Medicine (GUSOM) had 132 volunteer interviewers, of whom 38% had no prior experience with interviewing for the Office of Admissions. Faculty and staff constituted 71% of the group. Interviewers' self-reported gender identities were 56% female and 44% male, with no individuals selecting other gender identity categories. In the 2022 admissions cycle, GUSOM had 142 volunteer interviewers, of whom 60% were faculty and staff and 32% were new to interviewing. Overall self-identified genders were 43% female and 57% male, with no individuals selecting other categories. Given the medical school's mission to support an equitable admissions process, ABATE workshop attendance was required by the Office of Admissions for both the 2021 and 2022 interview cycles as a prerequisite for conducting interviews.

Results from all workshop questionnaire responses were aggregated. Likert-scale responses ranged from 0 (*strongly disagree*) to 4 (*strongly agree*). A few items were reverse scored (see note for Table 1). In analyses conducted to generate Figures 1 and 2, we combined responses of strongly agreed and agreed into one category and also combined responses of strongly disagreed and disagreed. Responses of neutral remained as their own category. These three categories were not assigned numerical values. Pre- and postworkshop questionnaire responses were not matched among participants. We used *t* tests to calculate differences between the pre- and postworkshop scores and calculated variances to determine use of appropriate *t*-test statistics based on whether the distribution indicated an equal or unequal variance.

Overall, the attendees reported that they found the ABATE workshop useful and relevant to improving their ability

Copyright © 2024 Nagubandi et al. This is an open-access publication distributed under the terms of the Creative Commons Attribution license.

Table 1. Statistical Results for Pre- and Postworkshop Questionnaires

Question[a]	No. Responses		*M*		Variance		*SD*		*SE*		*p*
	Pre	Post	Pre	Post	Pre	Post	Pre	Post	Pre	Post	
Kirkpatrick level 1											
Reaction-measuring question											
1. My prior training has adequately prepared me to minimize implicit bias during virtual interviews.	75	41	2.6	3.1	0.9	0.4	0.9	0.6	0.1	0.1	.002[b]
Kirkpatrick level 2											
Attitude-measuring questions											
2. I am confident in my abilities to conduct an unbiased virtual interview.	75	41	3.0	3.2	0.4	0.4	0.7	0.6	0.1	0.1	.04[b]
3. I am comfortable with saying that I am aware of any potential sources of implicit bias that may occur during virtual interviews.	75	41	3.0	3.4	0.6	0.3	0.7	0.6	0.1	0.1	.002[b]
4. I have experience with applying and identifying solutions that reduce implicit bias during my virtual interviews.	75	41	2.5	3.0	0.9	0.4	1.0	0.6	0.1	0.1	.003[b]
Knowledge-measuring questions											
5. Generally, an interviewer's perception of "likeness," or membership to an "ingroup" puts candidates at an advantage.[c]	73	38	2.8	2.8	1.3	1.7	1.1	1.3	0.1	0.2	.88
6. To address the potential undue influence of interview backgrounds on affinity bias and cultural matching, admissions offices and interview teams can identify strategies to neutralize the ways in which the background contributes to implicit bias.[c]	73	38	3.2	3.4	0.5	0.4	0.7	0.6	0.1	0.1	.04[b]
7. A candidate that is overweight doesn't understand what it means to be a leader and will not be as successful in medical school than other candidates who are not overweight.[c]	74	39	3.8	3.6	0.2	0.9	0.5	1.0	0.1	0.2	.48
8. Since we've all had to adjust virtually during the pandemic, there is no bias surrounding an interviewee's ability to make eye contact during virtual interviews.[c]	74	40	2.8	2.8	0.8	1.3	0.9	1.1	0.1	0.2	.86
9. The way one enunciates and pauses in their speaking has nothing to do with how you assess them.[c]	74	37	2.0	1.9	1.4	1.5	1.2	1.2	0.1	0.2	.62

[a]Eight of the nine questions were on a normal distribution; therefore, we performed a two-sample equal variance *t* test for questions 1-6, 8, and 9. Question number 7 had unequal variance, so the results shown for it are from a two-sample unequal variance *t* test. Specifically, for questions 1-6, values of 0-4 were assigned to strongly disagree–strongly agree, respectively, and questions 7-9 were reverse scored, with 0-4 assigned to strongly agree–strongly disagree, respectively.
[b]Statistically significant.
[c]Questions that began with the question stem "Based on your knowledge of the ABATE model for identifying bias please assess this statement" in the postworkshop questionnaire only; the question stem was absent in the preworkshop questionnaire.

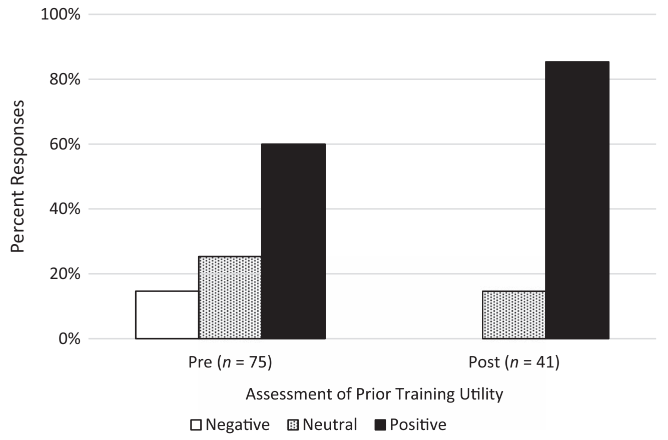

Figure 1. Pre- and postworkshop questionnaire results for level 1 (reaction) of the Kirkpatrick evaluation model. Question read as follows: "My prior training has adequately prepared me to minimize implicit bias during virtual interviews." Responses that marked strongly agree or agree were aggregated and considered positive, while responses that marked strongly disagree or disagree were aggregated and considered negative. Refer to Table 1 for exact means, standard deviations, and significance values of questionnaire responses (*n* values are indicated on the figure).

to minimize implicit bias during virtual interviews. Prior to completing the ABATE workshop, 40% of respondents either disagreed or had a neutral response whereas 60% of respondents agreed with the statement "My prior training has adequately prepared me to minimize implicit bias during virtual interviews" (question 1). After attending the workshop, no respondents disagreed with the statement, 15% had a neutral response, and 85% agreed (Figure 1). These pre- to postworkshop response shifts were statistically significant (M pre = 2.6, M post = 3.1, p = .002).

We also found statistically significant changes in attendees' attitudes about interviewing confidence (question 2: M pre = 3.0, M post = 3.2, p = .04), bias awareness (question 3: M pre = 3.0, M post = 3.4, p = .002), and solution identification and application (question 4: M pre = 2.5, M post = 3.0, p = .003). These results indicate improvements in interviewers' self-reported abilities to confidently conduct an unbiased virtual

Copyright © 2024 Nagubandi et al. This is an open-access publication distributed under the terms of the Creative Commons Attribution license.

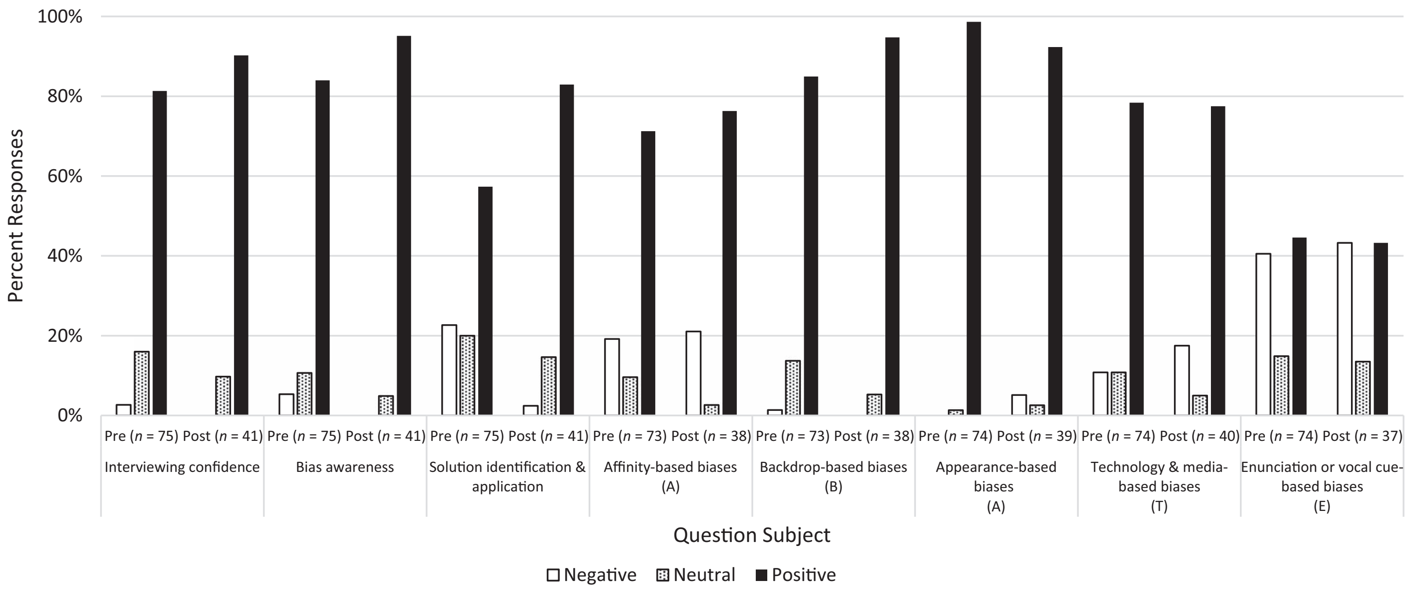

Figure 2. Pre- and postworkshop questionnaire results for level 2 (attitude and knowledge) of the Kirkpatrick evaluation model. Attitude-measuring questions included the following: (1) interviewing confidence statement: “I am confident in my abilities to conduct an unbiased virtual interview”; (2) bias awareness statement: “I am comfortable with saying that I am aware of any potential sources of implicit bias that may occur during virtual interviews”; and (3) solution identification/application statement: “I have experience with applying and identifying solutions that reduce implicit bias during my virtual interviews.” Responses that marked strongly agree or agree were aggregated and considered positive, while responses that marked strongly disagree or disagree were aggregated and considered negative. Knowledge-measuring questions began with the stem “Based on your knowledge of the ABATE model for identifying bias please assess this statement” in the postworkshop questionnaire only; the question stem was absent in the preworkshop questionnaire. The question statements included the following: (4) affinity biases: “Generally, an interviewer’s perception of ‘likeness,’ or membership to an ‘ingroup’ puts candidates at an advantage”; (5) backdrop biases: “To address the potential undue influence of interview backgrounds on affinity bias and cultural matching, admissions offices and interview teams can identify strategies to neutralize the ways in which the background contributes to implicit bias”; (6) appearance biases: “A candidate that is overweight doesn’t understand what it means to be a leader and will not be as successful in medical school than other candidates who are not overweight”; (7) technology and media biases: “Since we’ve all had to adjust virtually during the pandemic, there is no bias surrounding an interviewee’s ability to make eye contact during virtual interviews”; and (8) enunciation or vocal cue biases: “The way one enunciates and pauses in their speaking has nothing to do with how you assess them.” Responses that marked strongly agree or agree were aggregated and considered positive, while responses that marked strongly disagree or disagree were aggregated and considered negative for statements 4 and 5. Responses that marked strongly agree or agree were aggregated and considered negative, while responses that marked strongly disagree or disagree were aggregated and considered positive for statements 6-8. Please refer to Table 1 for exact means, standard deviations, and significance values of questionnaire responses (*n* values are indicated on the figure).

interview, recognize sources of implicit bias during virtual interviews, and apply mitigating solutions (Table 1 and Figure 2).

Notably, changes in responses to the attitude question 4 (“I have experience with applying and identifying solutions that reduce implicit bias during my virtual interviews”) reflected improvement in attendee confidence to mitigate biases that might arise during their virtual interviews after attending the workshop. Before attending the workshop, 23% of respondents disagreed with the statement whereas 57% of respondents agreed. After attending the workshop, only 2% of respondents disagreed with the statement while 83% of attendees agreed (Figure 2).

Of the five knowledge questions, the item assessing backdrop-based biases showed significant improvement from pre- to postworkshop (question 6: M pre = 3.2, M post = 3.4, p = .04; Figure 2). These results reinforce the role of admissions offices and interview teams in mitigating the effects of implicit biases based on candidates’ backgrounds during virtual interviews. We did not find any other statistically significant changes from pre- to postworkshop responses in the other four knowledge questions related to affinity-based (question 5: M pre = 2.8, M post = 2.8, p = .88), appearance-based (question 7: M pre = 3.8, M post = 3.6, p = .48), technology and media-based (question 8: M pre = 2.8, M post = 2.8, p = .86), and enunciation or vocal cue-based biases (question 9: M pre = 2.0, M post = 1.9, p = .62; see Table 1), potentially indicating lack of knowledge acquisition regarding these tenets of the ABATE model.

An open-ended question in the postworkshop questionnaire elicited attendees’ takeaways, feedback, and comments. Attendee responses were indicative of interviewers’ awareness of the pervasiveness of implicit biases in virtual admissions interviews, strong takeaways regarding bias mitigation techniques, and constructive feedback for

Copyright © 2024 Nagubandi et al. This is an open-access publication distributed under the terms of the Creative Commons Attribution license.

Table 2. Thematic Analysis of Open-Ended Responses on the Postworkshop Questionnaire

Theme	Representative Quotes/Feedback
Awareness of pervasiveness of bias	• "We all have biases as humans, but the important thing is to make sure we recognize them and attempt to address them."<br>• "It is important to be aware of how our perceptions inform our thoughts and use this to consciously make decisions of applicants based on their enthusiasm and ability as opposed to our feelings about the applicant."
Recognition of bias mitigation techniques	• "Always take pause and think about bias that may be at play when engaging with interviewees."<br>• "Subtle phrases in evaluations can draw particular scrutiny; I need to be as objective as possible to avoid drawing doubt while presenting a candid assessment of applicants."
Suggestions for the future	• "It would be good to emphasize racial and socio-economic bias more."

improving future workshops. Example quotes are included in Table 2.

Discussion

Bias, if unchecked, threatens the ability to fairly evaluate each candidate. If medical schools aim to uphold an equitable process that assesses the full abilities of all candidates holistically and equally, then virtual interviews require diligent and thoughtful design of new procedures. Despite the easing of pandemic restrictions, several medical schools have announced that interviews for future cycles will be held virtually. The current shift towards virtual interviews increases the need for the design and implementation of empirically supported bias mitigation tools that improve and reimagine what admissions offices can do to create more equitable and bias-free interview processes. These approaches are particularly important as the pool of candidates in medicine and health care continues to diversify.

Fundamentally, the greatest promise of the ABATE framework is in its focus on applying bias reduction techniques and promoting greater equity in the virtual assessment of candidates. The ABATE framework identifies five areas of implicit bias with potentially significant influence. Training to identify and rectify these biases can improve decisions in both in-person and virtual interviews for medical school admissions by mitigating the impact of implicit bias.

Results suggest that the ABATE training workshops for medical school admissions interviewers are relevant and increase interviewers' confidence and knowledge in identifying and rectifying sources of implicit bias in virtual interviews. The positive shift in attitudes seen among interviewers after the ABATE workshop is particularly promising, and future work can examine the effects of ABATE training on long-term behavioral changes. It is important to note that our institution has made a greater effort over recent years to shift institutional culture as we continue to develop and offer extensive training related to diversity, equity, and inclusion. Since personnel are exposed to other thematically similar training sessions before attending ABATE workshops, this could have influenced the positive shifts seen in reactions and attitudes surrounding ABATE training.

Limitations of this study include low questionnaire response rates for all sessions, with notable drop-off for the postworkshop questionnaire for the 2021 sessions. The low response rate can likely be attributed to the nonmandatory nature of the questionnaires. We could not assess changes at the level of individual interviewers given that the pre- and postworkshop evaluation responses were anonymized and thus not paired. Additionally, we could not analyze how attendee diversity influenced the effectiveness of the ABATE model given the lack of self-identified race and ethnicity attendee data for the 2021 and 2022 admissions cycles. In light of this limitation, leadership within the Office of Admissions has recognized the importance of expanding demographic data collection to include consistent data on self-identified race and ethnicity, among other demographic characteristics. This system-level change is being implemented from the 2023 admissions cycle onwards. Among the 2023-2024 interviewer cohort, data are as follows: 29% Asian, 4% Black, 4% Hispanic, 2% Middle Eastern/North African, 2% Native American/Hawaiian, 52% White, and 2% mixed race. As two-thirds of interviewers return each year, these data represent a proxy for the characteristics of interviewers in prior years.

Another limitation is selection bias. Although the attitude questions and the reaction question were required in the pre- and postworkshop questionnaires, responses to the knowledge questions were not mandatory. Therefore, our analysis of the knowledge-question responses may not adequately represent the knowledge of the entire sample of interviewers who attended the workshop. We also saw a lack of statistically significant improvements among most of the knowledge questions. This could have been driven by a ceiling effect of high preworkshop knowledge among attendees. Because workshop attendees experience a scaffolded approach to interviewer training at GUSOM, they may enter the virtual interview-specific ABATE

Copyright © 2024 Nagubandi et al. This is an open-access publication distributed under the terms of the Creative Commons Attribution license.

training with high baseline levels of knowledge. Future efforts could explore new or expanded types of content to further enrich interviewers' knowledge and application of knowledge learned in ABATE workshops.

One final limitation is that the workshop was a onetime session held at the beginning of the admissions interview cycle. While the present results suggest that a onetime ABATE workshop is effective, offering multiple workshops so that attendees could share their experiences during the interview cycle could allow for deeper understanding and reflection regarding one's own biases. This workshop should be viewed not as a one and done event but as a foundation for additional training sessions and processes that medical school admissions offices can undertake to help mitigate the effect of implicit biases in virtual interviews.

The ABATE model includes details regarding implementation at three levels: institutional, individual interviewer, and interviewee/candidate (Appendix B). The present evaluation of the ABATE workshop for admissions interviewers suggests that the model provides an approach that can be implemented in medical education selection processes at other levels, such as the departmental and medical center levels, with parallel committees focused on recruiting, selecting, interviewing, and evaluating candidates for faculty hiring, appointments, and promotions. The ABATE model supports an efficient and practical way to address biases in the virtual interview setting so that institutional and community commitments to diversity, equity, and inclusion can be upheld.

Future iterations of the ABATE workshop will incorporate a range of improvements such as the following:

1. Including a second facilitator to assist with technology management and expertise.
2. Collecting self-identified race and ethnicity data to better measure the impact of demographics on ABATE training reception.
3. Requiring mandatory questionnaire responses, with a "prefer not to answer" option for each question, to boost response rates. Participant pre-post responses can be tracked using a unique participant-generated deidentified code.
4. Rephrasing questionnaires as questions rather than statements to increase clarity.
5. Conducting individual interviews to elicit additional feedback about areas for improvement.
6. Matching pre- and postworkshop questionnaire responses to assess changes at the interviewer level.
7. Establishing interactive exercises that evaluate longitudinal learning and behavioral outcomes following ABATE training to best capture the full range of knowledge, attitudes, and skills over time.

Ultimately, the ABATE workshop model represents a brief intervention into identifying and mitigating sources of implicit bias in virtual interviews. These efforts provide insights into specific faculty training and institutional initiatives that can put values related to supporting diversity, equity, and inclusion into practice.

Appendices

A. ABATE Framework.docx
B. Levels of Implementation.docx
C. Preworkshop Evaluation Questionnaire.docx
D. Postworkshop Evaluation Questionnaire.docx
E. ABATE Slide Deck.pptx
F. ABATE Speaker Notes.docx

All appendices are peer reviewed as integral parts of the Original Publication.

Veda Nagubandi: Research Fellow, Office of Diversity, Equity, Inclusion, & Belonging, Georgetown University School of Medicine

Caleb C. McKinney, PhD, MPS: Associate Professor, Department of Rehabilitation Medicine, Associate Dean of Graduate and Postdoctoral Training & Development, Biomedical Graduate Education, and Assistant Vice President of Master's Program Administration & Development, Georgetown University Medical Center; ORCID: https://orcid.org/0000-0001-7573-3899

Nicole M. Houle, EdD: Associate Dean for Admissions and Financial Aid, Office of Admissions and Financial Aid, Georgetown University School of Medicine; ORCID: https://orcid.org/0009-0002-3868-7875

Kristi D. Graves, PhD: Associate Professor of Oncology, Department of Oncology, and Associate Director of Diversity, Equity and Inclusion, Lombardi Comprehensive Cancer Center, Georgetown University School of Medicine; ORCID: https://orcid.org/0000-0001-8808-1631

Susan M. Cheng, EdLD, MPP: Associate Professor, Department of Family Medicine, and Senior Associate Dean for Diversity, Equity, Inclusion, & Belonging, Office of Diversity, Equity, Inclusion, & Belonging, Georgetown University School of Medicine; ORCID: https://orcid.org/0000-0001-5536-0802

Disclosures:
None to report.

Funding/Support:
None to report.

Copyright © 2024 Nagubandi et al. This is an open-access publication distributed under the terms of the Creative Commons Attribution license.

Ethical Approval:
The Georgetown University Institutional Review Board deemed further review of this project not necessary.

References

1. Staats C, Capatosto K, Wright RA, Jackson VW. *State of the Science: Implicit Bias Review*. 4th ed. Kirwan Institute; 2016.
2. Ross HJ. *Everyday Bias: Identifying and Navigating Unconscious Judgments in Our Daily Lives*. Rowman & Littlefield; 2020.
3. Blair IV. The malleability of automatic stereotypes and prejudice. *Pers Soc Psychol Rev*. 2002;6(3):242-261. https://doi.org/10.1207/S15327957PSPR0603_8
4. Nosek BA, Smyth FL, Hansen JJ, et al. Pervasiveness and correlates of implicit attitudes and stereotypes. *Eur Rev Soc Psychol*. 2007;18(1):36-88. https://doi.org/10.1080/10463280701489053
5. Graves LM, Powell GN. The effect of sex similarity on recruiters' evaluations of actual applicants: a test of the similarity-attraction paradigm. *Pers Psychol*. 1995;48(1):85-98. https://doi.org/10.1111/j.1744-6570.1995.tb01747.x
6. Labor & No Intent, No Foul? Employment. Published May 21, 2007. Accessed August 2, 2020. https://www.law.du.edu/documents/employment-and-labor-law/publications-katz-no-intent-no-foul.pdf
7. Uhlmann EL, Cohen GL. "I think it, therefore it's true": effects of self-perceived objectivity on hiring discrimination. *Organ Behav Hum Decis Process*. 2007;104(2):207-223. https://doi.org/10.1016/j.obhdp.2007.07.001
8. Greenwald AG, McGhee DE, Schwartz JLK. Measuring individual differences in implicit cognition: the implicit association test. *J Pers Soc Psychol*. 1998;74(6):1464-1480. https://doi.org/10.1037//0022-3514.74.6.1464
9. Towaij C, Gawad N, Alibhai K, Doan D, Raîche I. Trust me, I know them: assessing interpersonal bias in surgery residency interviews. *J Grad Med Educ*. 2022;14(3):289-294. https://doi.org/10.4300/JGME-D-21-00882.1
10. Redelmeier DA, Baxter SD. Rainy weather and medical school admission interviews. *CMAJ*. 2009;181(12):933. https://doi.org/10.1503/cmaj.091546
11. Bertrand M, Chugh D, Mullainathan S. Implicit discrimination. *Am Econ Rev*. 2005;95(2):94-98. https://doi.org/10.1257/000282805774670365
12. Blair IV, Ma JE, Lenton AP. Imagining stereotypes away: the moderation of implicit stereotypes through mental imagery. *J Pers Soc Psychol*. 2001;81(5):828-841. https://doi.org/10.1037//0022-3514.81.5.828
13. Conducting interviews during the coronavirus pandemic. Association of American Medical Colleges. May 14, 2020. Accessed July 30, 2020. https://www.aamc.org/what-we-do/mission-areas/medical-education/conducting-interviews-during-coronavirus-pandemic
14. *Final Report and Recommendations for Medical Education Institutions of LCME-Accredited, U.S. Osteopathic, and Non-U.S. Medical School Applicants*. Association of American Medical Colleges. May 11, 2020. Accessed May 14, 2024. https://www.aamc.org/system/files/2020-05/covid19_Final_Recommendations_05112020.pdf
15. Sternberg K, Jordan J, Haas MRC, et al. Reimagining residency selection: part 2—a practical guide to interviewing in the post-COVID-19 era. *J Grad Med Educ*. 2020;12(5):545-549. https://doi.org/10.4300/JGME-D-20-00911.1
16. Weiner S. Applications to medical school are at an all-time high. What does this mean for applicants and schools? Association of American Medical Colleges. October 22, 2020. Accessed May 14, 2024. https://www.aamc.org/news-insights/applications-medical-school-are-all-time-high-what-does-mean-applicants-and-schools
17. Seifi A, Mirahmadizadeh A, Eslami V. Perception of medical students and residents about virtual interviews for residency applications in the United States. *PLoS One*. 2020;15(8):e0238239. https://doi.org/10.1371/journal.pone.0238239
18. Interviews in GME: where do we go from here? Association of American Medical Colleges. May 2023. Accessed May 14, 2024. https://www.aamc.org/about-us/mission-areas/medical-education/interviews-gme-where-do-we-go-here
19. Rivera LA. Hiring as cultural matching: the case of elite professional service firms. *Am Sociol Rev*. 2012;77(6):999-1022. https://doi.org/10.1177/0003122412463213
20. Davis MG, Haas MRC, Gottlieb M, House JB, Huang RD, Hopson LR. Zooming in versus flying out: virtual residency interviews in the era of COVID-19. *AEM Educ Train*. 2020;4(4):443-446. https://doi.org/10.1002/aet2.10486
21. Maxfield CM, Thorpe MP, Desser TS, et al. Bias in radiology resident selection: do we discriminate against the obese and unattractive? *Acad Med*. 2019;94(11):1774-1780. https://doi.org/10.1097/ACM.0000000000002813
22. Sears GJ, Zhang H, Wiesner WH, Hackett RD, Yuan Y. A comparative assessment of videoconference and face-to-face employment interviews. *Manag Decis*. 2013;51(8):1733-1752. https://doi.org/10.1108/MD-09-2012-0642
23. Marbin J, Hutchinson YV, Schaeffer S. Avoiding the virtual pitfall: identifying and mitigating biases in graduate medical education videoconference interviews. *Acad Med*. 2021;96(8):1120-1124. https://doi.org/10.1097/ACM.0000000000003914
24. Lewis S. The racial bias built into photography. *New York Times*. April 25, 2019. Accessed May 14, 2024. https://www.nytimes.com/2019/04/25/lens/sarah-lewis-racial-bias-photography.html
25. Brow BL, Strong WJ, Rencher AC. Perceptions of personality from speech: effects of manipulations of acoustical parameters.

Copyright © 2024 Nagubandi et al. This is an open-access publication distributed under the terms of the Creative Commons Attribution license.

J Acoust Soc Am. 1973;54(1):29-35. https://doi.org/10.1121/1.1913571

26. Segrest Purkiss SL, Perrewé PL, Gillespie TL, Mayes BT, Ferris GR. Implicit sources of bias in employment interview judgments and decisions. *Organ Behav Hum Decis Process*. 2006;101(2): 152-167. https://doi.org/10.1016/j.obhdp.2006.06.005
27. Premed Competencies resources. Association of American Medical Colleges. Accessed May 14, 2024. https://www.aamc.org/services/admissions-lifecycle/competencies-entering-medical-students
28. Robertson AC, Fowler LC, Niconchuk J, et al. Application of Kern's 6-step approach in the development of a novel anesthesiology curriculum for perioperative code status and goals of care discussions. *J Educ Perioper Med*. 2019;21(1):E634.
29. The Kirkpatrick model. Kirkpatrick Partners. Accessed May 14, 2024. https://www.kirkpatrickpartners.com/the-kirkpatrick-model/
30. Rouse DN. Employing Kirkpatrick's evaluation framework to determine the effectiveness of health information management courses and programs. *Perspect Health Inf Manag*. 2011;8(Spring):1c.

Received: July 31, 2023
Accepted: March 4, 2024
Published: June 28, 2024

Copyright © 2024 Nagubandi et al. This is an open-access publication distributed under the terms of the Creative Commons Attribution license.
